# ^13^C and ^15^N NMR identification of product compound classes from aqueous and solid phase photodegradation of 2,4,6-trinitrotoluene

**DOI:** 10.1371/journal.pone.0224112

**Published:** 2019-10-22

**Authors:** Kevin A. Thorn

**Affiliations:** U.S. Geological Survey, Denver Federal Center, Denver, Colorado, United States of America; University of Iowa, UNITED STATES

## Abstract

Photolysis is one of the main transformation pathways for 2,4,6-trinitrotoluene (TNT) released into the environment. Upon exposure to sunlight, TNT is known to undergo both oxidation and reduction reactions with release of nitrite, nitrate, and ammonium ions, followed by condensation reactions of the oxidation and reduction products. In this study, compound classes of transformation products from the aqueous and solid phase photodegradation of 2,4,6-trinitrotoluene (TNT) have been identified by liquid and solid state ^13^C and ^15^N NMR. Aqueous phase experiments were performed on saturated solutions of T^15^NT in deionized water, natural pond water (pH = 8.3, DOC = 3.0 mg/L), pH 8.0 buffer solution, and in the presence of Suwannee River Natural Organic Matter (SRNOM; pH = 3.7), using a Pyrex-filtered medium pressure mercury lamp. Natural sunlight irradiations were performed on TNT in the solid phase and dissolved in the pond water. In deionized water, carboxylic acid, aldehyde, aromatic amine, primary amide, azoxy, nitrosophenol, and azo compounds were formed. ^15^N NMR spectra exhibited major peaks centered at 128 to 138 ppm, which are in the range of phenylhydroxylamine and secondary amide nitrogens. The secondary amides are proposed to represent benzanilides, which would arise from photochemical rearrangement of nitrones formed from the condensation of benzaldehyde and phenylhydroxylamine derivatives of TNT. The same compound classes were formed from sunlight irradiation of TNT in the solid phase. Whereas carboxylic acids, aldehydes, aromatic amines, phenylhydroxylamines, and amides were also formed from irradiation of TNT in pond water and in pH 8 buffer solution, azoxy and azo compound formation was inhibited. Solid state ^15^N NMR spectra of photolysates from the lamp irradiation of unlabeled 2,6-dinitrotoluene in deionized water also demonstrated the formation of aromatic amine, phenylhydroxylamine/ 2° amide, azoxy, and azo nitrogens.

## Introduction

### Background

The major transformation pathways of 2,4,6-trinitrotoluene (TNT) released into the environment include microbial reduction, metal catalyzed abiotic reduction, and photolysis, depending on the individual soil, sediment, or aquatic matrix [1–15]. TNT is also susceptible to alkaline hydrolysis, but the conditions of pH ≥ 9 required for significant hydrolysis to occur are not relevant to most environments. Because of the electron-withdrawing properties of the nitro groups, the aromatic ring of TNT is resistant to attack by electrophiles such as O_2_, and therefore complete mineralization of TNT by microbes is not generally considered a significant fate [16]. However, recent work has recorded rates of bacterial incorporation and mineralization in coastal marine sediments, where nitrogen limitation of the environment may be a factor [17]. Aromatic amines resulting from microbial or abiotic reduction of TNT may undergo covalent binding to the naturally occurring organic matter in soils and sediments to form bound residues [18–20]. TNT is known to undergo intense coloration in both the solution and solid phase when exposed to sunlight [4, 21–23], in the latter case to an orange brown color, by itself and as a component of Composition B (60:39:1 mixture of RDX, TNT, and wax). In the presence of sunlight, aqueous solutions of TNT initially turn pink and then gradually darken over a period of 4 to 6 hours to a cloudy, rusty-orange colored solution referred to as “pink water” [21]. (The term “red water” or “sellite water” refers to a separate type of wastewater produced in the manufacturing process during purification of crude TNT with sodium sulfite.)

Formation of degradation products via photolysis is of concern in several types of environments where TNT contamination occurs. Prior to the establishment of environmental regulations in the 1970’s, wastewater streams saturated with TNT and RDX generated during the manufacture, the load, assembly and packing (LAP) operations, and the decommissioning of outdated munitions were directed to lagoons for primary settling of solid munitions material before the water was released to streams and rivers [24]. Evaporation left the washout lagoon soils heavily contaminated with the parent compounds, degradation products from dissolved or solid phase photolysis, and the microbial and abiotic reduction products formed in the soils and sediments. Leaching of these contaminants into groundwater has visibly discolored the water red at a number of these sites.

On military ranges, unexploded ordnance, low-order detonations, and high explosive residues from munitions that detonate properly are sources of contamination to soils as a result of live-fire training [25]. Low order detonations, which scatter centimeter and millimeter size particles of TNT and Composition B, are considered to be the main source of contamination on ranges at present [25]. Products of photodecomposition of TNT have been observed as reddish brown to orange coatings on TNT and Composition B (CompB) particles and as fine powdered residues surrounding TNT and CompB particles on ranges receiving limited rainfall and wind dispersion [22]. Photographs of such discolored chunks and particles have been reported [4, 23]. Photodegradation products of TNT, specifically 1,3,5-trinitrobenzene, 1,3-dinitrobenzene, and 3,5-dinitroaniline, have often been detected in range soils during the execution of range characterization studies [25]. The photodegradation products 1,3,5-trinitrobenzene, 3,5-dinitroaniline, and 3,5-dinitrophenol have also been detected in groundwater contaminated from a former ammunition destruction site [26–28].

TNT may also enter the marine environment through corrosion of unexploded ordnance at dumping grounds and run-off from coastal artillery ranges. Where TNT reaches the photic zone in surface waters or where light intensity is adequate, it may be assumed that photolysis takes place. Studies have detected TNT in surrounding sediments or in the water column derived from underwater unexploded ordnance [1, 12, 29–32].

### Aqueous phase photodegradation

The most extensive studies undertaken to analyze the constituents and their formation pathways in pink water were conducted by Burlinson and Kaplan [21, 33–37]. From solutions of approximately 120 mg/kg TNT in distilled water irradiated with a Pyrex- filtered (cutoff of irradiation below 280 nm) medium pressure mercury lamp, they determined that 45–50% of the photodecomposition products of TNT were recovered in solution as 16 identifiable structures, listed in Table 1 and S1 Fig, including the direct alcohol, aldehyde, and carboxylic acid oxidation products of TNT, as well as nitroaniline, nitrophenol, benzisoxazole, benzaldoxime, benzonitrile, azobenzene, azoxybenzene and benzanilide compounds, the latter three being dimerization products. The major products were 2,4,6-trinitrobenzaldehyde, 4,6-dinitro-1,2-benzisoxazole, 2,2’-dicarboxylic-2,3’,5,5’-tetranitro-azobenzene and 2-amino-4,6-dinitrobenzoic acid. After separation from the product mixture through solvent extraction followed by thin layer chromatography, these degradation products were identified by a combination of gas chromatography-mass spectrometry (GC/MS), infrared spectrometry, and ^1^H NMR spectrometry. The remainder of the decomposition products, consisting of an insoluble reddish brown residue, were not identified but postulated to consist of oligomers of azo or azoxy compounds. The effects of pH, concentration, and wavelength range of the irradiation were also examined. The rate of photochemical degradation in water was inversely proportional to acidity over the pH range from 1.1 to 11.1. Whereas aqueous solutions of TNT are stable in the dark at neutral and acidic pH, hydrolysis becomes a limited factor at pH above neutrality. Thus the photodegradation rates at higher pH values may have included a contribution from alkaline hydrolysis [34]. The distribution of photodecomposition products from irradiation of pH 7 buffered TNT solutions at concentrations of 113 mg/L and 4.5 mg/L showed no significant differences. Examining the effect of the Pyrex filter, the rate of TNT degradation was significantly higher with the unfiltered lamp, and the chromatographable products did not match those from the Pyrex-filtered irradiation. Although 2,4,6-trinitrobenzaldehyde was the major photodegradation product in distilled water, 1,3,5-trinitrobenzene, which proceeds from the former via oxidation and decarboxylation steps, was found to be the major product in river water at pH 8.2 [33].

**Table 1:** Reported photodegradation products of TNT.

Compound	Matrix	Source	Ref
1,3,5-Trinitrobenzene	Distilled water	UV Lamp	34
(1,3,5-TNB)	Milli-Q water	Sunlight	26
	Seawater	Solar Simulator	43
4,6-Dinitro-1,2-benzisoxazole	Distilled water	UV Lamp	34
(4,6-dinitroanthranil)	Solid	Sunlight	49
2,4,6-Trinitrobenzaldehyde	Distilled water	UV Lamp	34
(2,4,6-TNBzald)	Seawater	Solar Simulator	43
	Solid	Sunlight	49
2,4,6-Trinitrobenzyl alcohol	Distilled water	UV Lamp	34
(2,4,6-TNBOH)	Solid	Sunlight	49
3,5-Dinitrophenol	Distilled water	UV Lamp	34
(3,5-DNP)	Milli-Q water	Sunlight	26
4,6-Dinitro-2,1-benzisoxazole	Distilled water	UV Lamp	34
(4,6-dinitroisoanthril)			
2,4,6-Trinitrobenzaldoxime	Distilled water	UV Lamp	34
2,4,6-Trinitrobenzonitrile	Distilled water	UV Lamp	34
1,3,7,9-Tetranitroindazolo-	Distilled water	UV Lamp	34
2,1-a-indazol-6-ol-12-one			
2,2'-Dicarboxy-3,3',5,5'-	Distilled water	UV Lamp	34
tetranitroazoxybenzene	Milli-Q water	Sunlight	26
	Solid	Sunlight	49
2,2'-Dicarboxy-3,3',5,5'-	Distilled water	UV Lamp	34
tetranitroazobenzene	Solid	Sunlight	49
2-Carboxy-3,3',5,5'-	Distilled water	UV Lamp	34
tetranitro-NNO-azoxy benzene	Solid	Sunlight	49
2,4,6-Trinitrobenzoic acid	Distilled water	UV Lamp	34
(2,4,6-TNBA)	Milli-Q water	Sunlight	26
	Seawater	Solar Simulator	43
N-(2-carboxy-3,5-dinitrophenyl)	Distilled water	UV Lamp	34
-2,4,6-trinitrobenzamide			
2-Amino-4,6-dinitrobenzoic acid	Distilled water	UV Lamp	34
(2-A-4,6-DNBA)	Seawater	Solar Simulator	43
	Weathered TNT Chunks	Sunlight	23
2-Hydroxy-4,6-dinitrobenzoic acid	Milli-Q water	Sunlight	26
(2-OH-4,6-DNBA)			
2,4-Dinitrobenzoic acid	Detected in lagoon water	Sunlight	53
(2,4-DNBA)			
1,3-Dinitrobenzene	Detected in lagoon water	Sunlight	53
(1,3-DNB)			
3,5-Dinitroaniline	Detected in lagoon water	Sunlight	53
(3,5-DNA)	Solid	Sunlight	49
2-Amino-4,6-dinitrotoluene	Weathered TNT Chunks	Sunlight	23
(2ADNT)			
4-Amino-2,6-dinitrotoluene	Weathered TNT Chunks	Sunlight	23
(4ADNT)			

The effects of dissolved organic matter (DOM; alternatively referred to as natural organic matter (NOM) or aquatic humic substances), salinity and nitrate on the rates of TNT photolysis have been further elaborated [34]. In general, NOM can affect photolysis of aquatic organic contaminants by acting as a photosensitizer through production of excited organic matter itself and reactive oxygen species such as singlet oxygen and hydroxyl radical, and by attenuating the light reaching the contaminant. NOM can serve as both a source and sink of hydroxyl radical [38]. Spanggord et al. and Mabey et al. observed that photolysis of TNT proceeds more rapidly in river and pond water than in distilled water, both attributing the accelerated rates to the presence of NOM [39, 40]. Mabey provided evidence that during aqueous photolysis of TNT in natural waters the humic substances act as triplet sensitizers, and speculated that complexes (e.g. charge transfer) form between TNT and the humic substances [39]. For nitroaromatic compounds in general, humic substances were found to enhance the sunlight-induced photodegradation rates compared to rates measured in distilled water, the largest increases occurring in the case of methyl group substitution ortho to the nitro group [41]. O’Sullivan reported that the rate of TNT photolysis decreased in the order seawater > estuarine water > fresh water > pure water, the rate of disappearance following first order kinetics in each case [42]. Transformation was driven mainly by wavelengths between 295 and 395 nm. Although addition of nitrate up to 4mM in pure water increased the rate of photolysis, addition up to 600μM in seawater did not change the photolysis half-life. Luning-Prak examined the effects of pH, temperature, salinity and DOM on photolysis rates of TNT in seawater [43]. Photolysis rates were not significantly affected in reducing pH from 8 to 6.4, increased slightly with increases in temperature from 10 to 32°C, increased with increasing salinity, attributed to a secondary kinetic salt effect, and increased with addition of NOM to seawater up to 5mg/L in the form of Suwannee River fulvic acid. The authors concurred with Mabey that the NOM acted as a triplet sensitizer in TNT photolysis. Addition of NOM to ultrapure water increased photolysis rates more markedly than to seawater. Effects of these factors on photolysis of 2,4-dinitrotoluene (2,4DNT) and 2,6-dinitrotoluene (2,6DNT) were also examined [44]. Reports on the effects of dissolved oxygen on TNT photolysis rates are inconsistent. Mabey et al. reported 3-fold greater rate constants in nitrogen purged over oxygen-purged solutions of 1 ppm TNT in pure water from both sunlight and lamp irradiations [39]. Wang and Kutal reported faster degradation rates in oxygen- over nitrogen purged 10 ppm solutions of TNT in deionized water, with colored products formed under both aerobic and anaerobic conditions [45]. The different concentrations and light sources employed in the two studies may possibly account for the varying results. Prolonged lamp UV irradiation is required for ring opening of TNT by direct photolysis. In experiments with ^14^C ring-labeled TNT, Andrews and Osmon reported 9 to 15% of initial activity recovered as ^14^CO_2_ after 24 to 72 hours irradiation [46].

### Solid phase photodegradation

A more limited number of studies have addressed photodegradation of TNT in the solid state [22, 23, 47–49]. 2-Amino-4,6-dinitrobenzoic acid, 2-amino-4,6-dinitrotoluene (2ADNT), and 4-amino-2,6-dinitrotoluene (4ADNT) were detected by LC/MS in leachates from TNT chunks exposed to outside weathering including sunlight, rain, and snow [23]. An isoxazole compound designated RP2 (red product #2: 4,6-dinitro-5-(2,4,6-trinitrobenzyl)-4,5-dihydrobenzo[c]isoxazole) was proposed to be responsible for the red color of the leachate [23]. Isomers of aminodinitrobenzoic acid, dinitrotoluene, aminodinitrotoluene, trinitrobenzene, and dinitroaniline were among compounds identified by LC/MS from lamp irradiations of solid TNT [22]. Although substitution patterns were not confirmed, these were likely the 2-amino-4,6-dinitrobenzoic acid, 2,4- and 2,6-DNT, 2- and 4-ADNT, 1,3,5-trinitrobenzene, and 3,5-dinitroaniline compounds reported in the literature. Products from lamp irradiations of TNT and CompB in soil matrices were also analyzed [22]. In examining the effects of water, iron oxide, and K^+^ contents on the degradation of TNT in whole soils by UV photolysis, Im et al. found water content the most important factor, its concentration positively correlated with disappearance rate [48].

Kunz et al. examined the degradation of solid TNT exposed to sunlight under meteorological regimes of desert summer and temperate spring, summer and winter [49]. Twenty one degradation products were identified using DART-TOF/MS (direct analysis in real time-time of flight), including four benzisoxazole, four azo, and four azoxy compounds. Several of the compounds were among those also identified as aqueous phase degradation products: 2,4,6-trinitrobenzaldehyde; 2,4,6-trinitrobenzyl alcohol; 4,6-dinitro-1,2-benzisoxazole; 3,5-dinitroaniline; 2-carboxy-3,3',5,5'-tetranitro-NNO-azoxy benzene; 2,2'-dicarboxy-3,3',5,5'-tetranitroazoxybenzene; 2,2'-dicarboxy-3,3',5,5'-tetranitroazobenzene. The authors determined that the fractional rates for both sublimation and photodegradation are proportional to the surface-area to volume ratio, and that most TNT particles are only photochemically altered at their surface because of the shallow penetration depth of UV solar irradiation. The azo and azoxy compounds have lower vapor pressures than the parent TNT. Their formation at the surface of TNT particles contributed to a depression of the sublimation rate of the TNT over time. Extensive irradiation lead to the formation of insoluble oligomers that exceeded molecular weights of 1500.

### Advanced oxidation processes

Advanced oxidation processes for the destruction of TNT in contaminated wastewaters have received considerable attention [50], and several of these exploit UV irradiation, for instance UV irradiation in conjunction with H_2_O_2_, TiO_2_, O_3_, and Fenton reagent. As a number of these studies have examined direct photolysis of TNT as a control reaction, they also provide insight into the photochemical transformation of TNT.

### Transformation pathways

**Fig 1:**
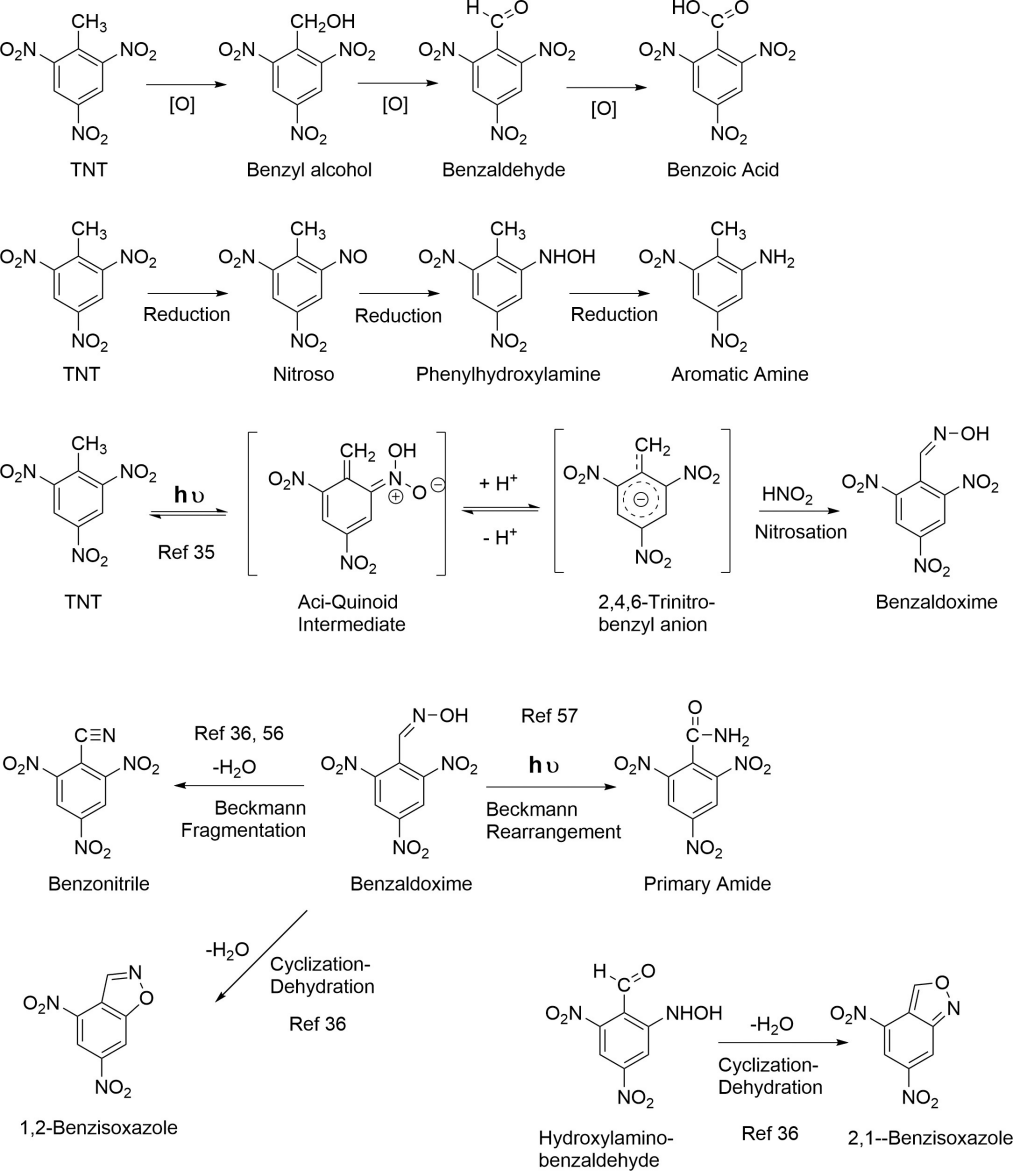
Photochemical transformation reactions of TNT.

**Fig 2:**
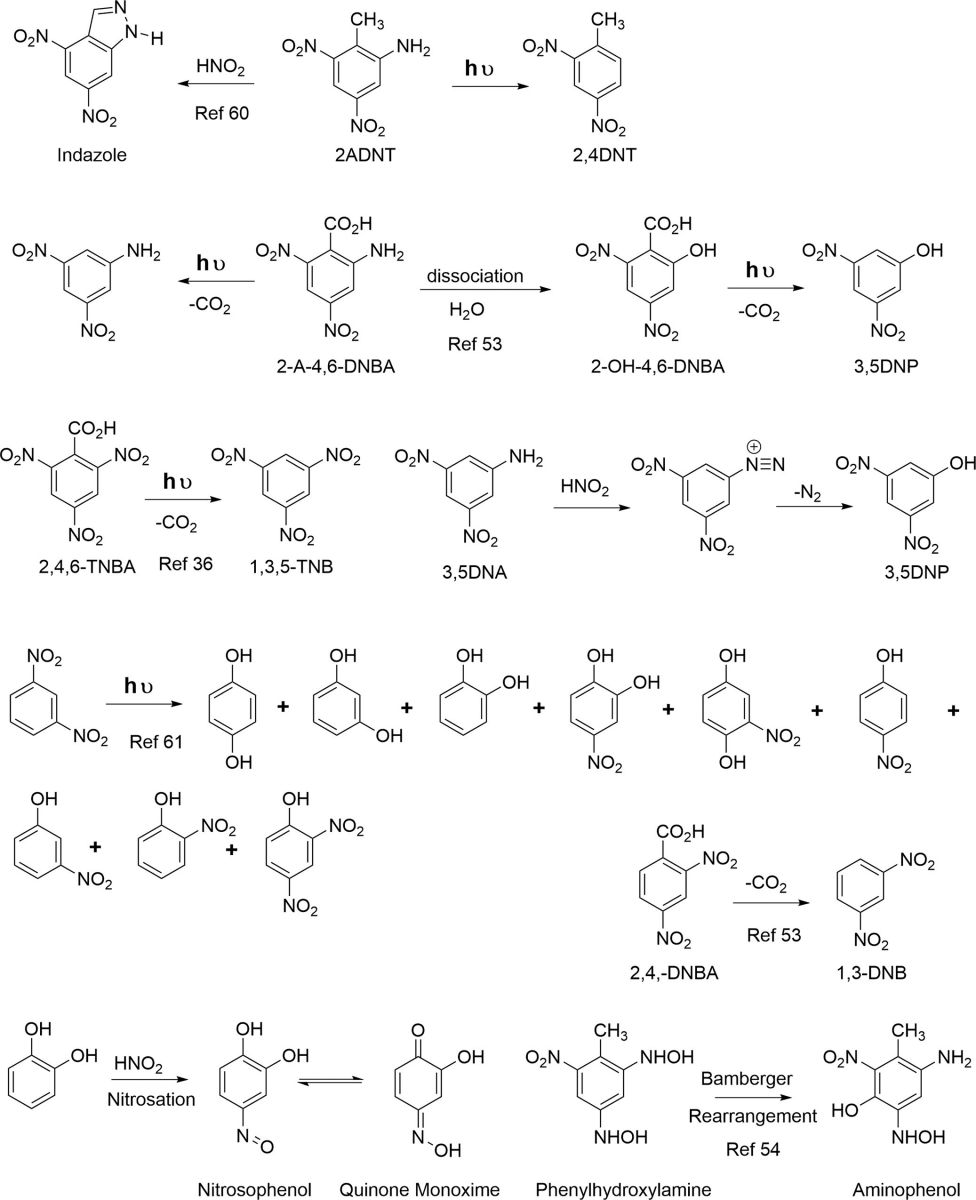
Photochemical transformation reactions of TNT.

**Fig 3:**
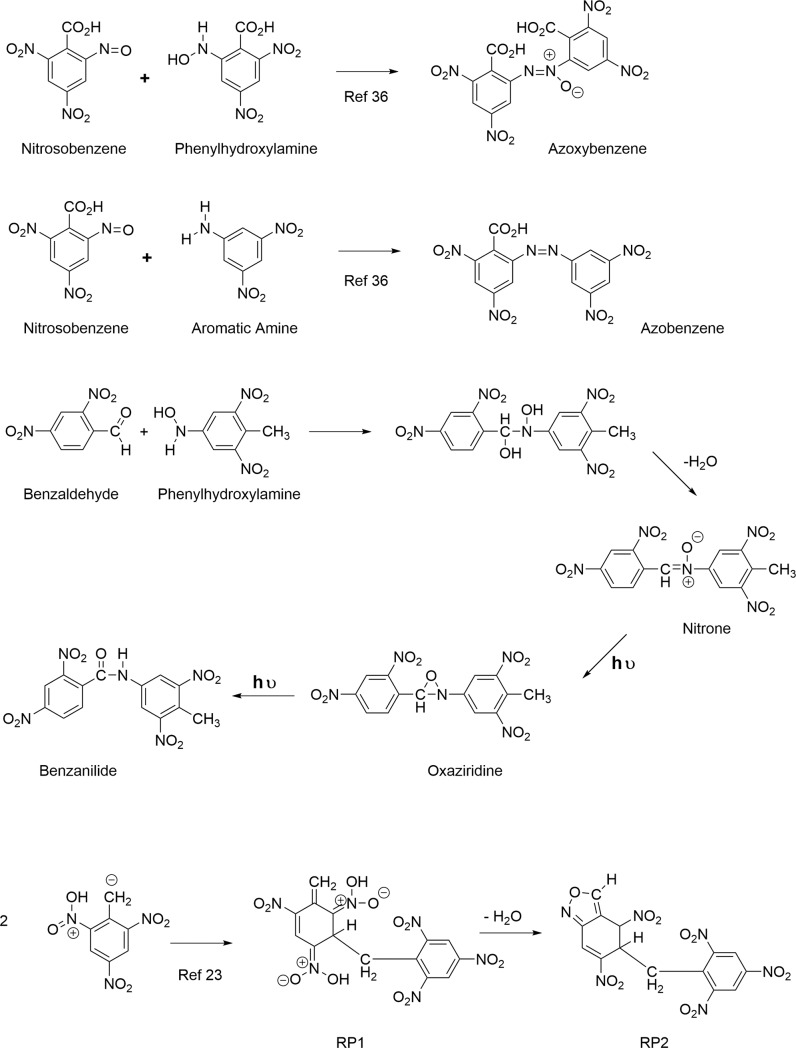
Photochemical transformation reactions of TNT.

**Fig 4:**
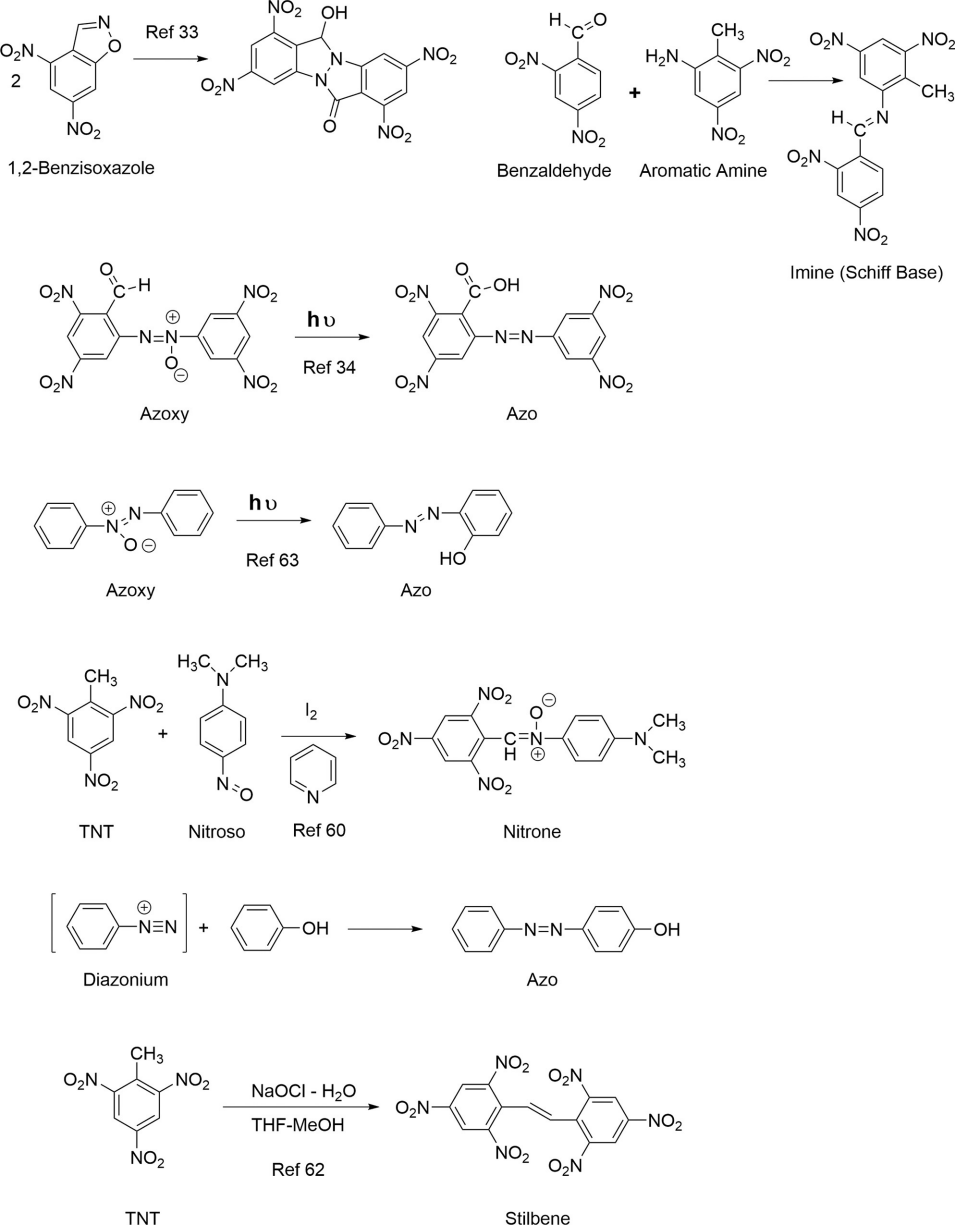
Photochemical transformation reactions of TNT.

**Fig 5:**
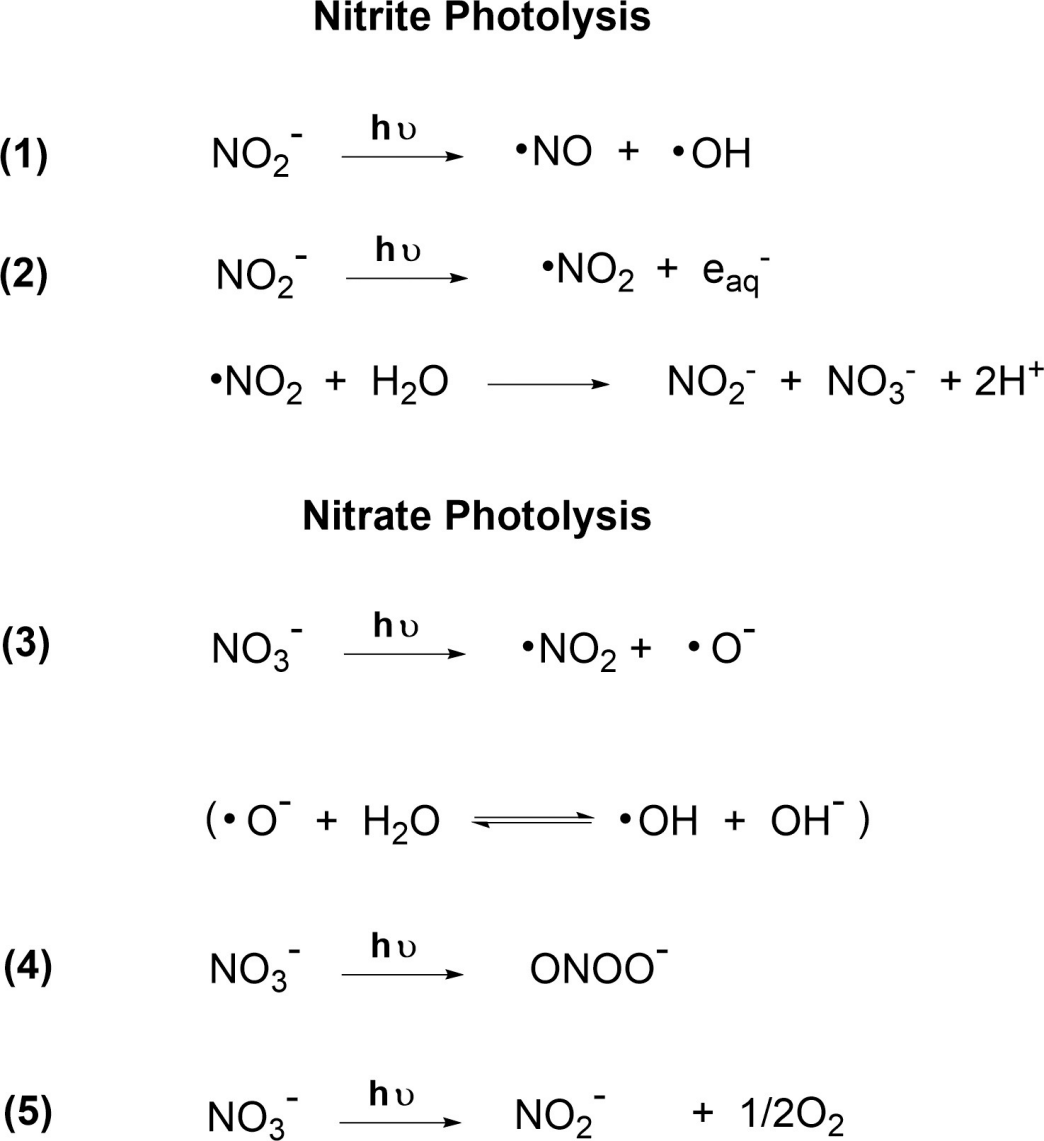
Nitrite and nitrate photolysis reactions.

TNT absorbs radiation between 200 and 280 nm strongly and up to 400 nm less so, with an absorption peak at 230 nm and a low intensity peak near 340 nm [51–53]. Pathways leading to functional group transformations and condensation reactions upon irradiation of TNT are illustrated in Figs 1–5. Redox reactions are of primary importance. Oxidation at the methyl group of TNT leads successively to the benzylic alcohol, aldehyde, and carboxylic acid, all of which have been reported in photodegradation studies (Fig 1; Table 1). Reduction at the nitro group leads successively to nitroso, hydroxylamino, and aromatic amine derivatives (Fig 1). The monoamine reduction products 2ADNT and 4ADNT, but not their reactive nitroso and hydroxylamino precursors, have been reported in photodegradation studies (Table 1). Phenylhydroxylamines are known to undergo the Bamberger rearrangement to aminophenols; the rearrangement has been documented in studies on the microbial reduction of TNT, but not 2,4- or 2,6-DNT (Fig 2)[54].

Nitrosation reactions have been implicated in formation of several of the photodegradation products identified by Burlinson and Kaplan [21, 34–36]. As an initial photoproduct of TNT in aqueous solution, the excited 1,2,6-trinitrobenzyl anion [55] intermediate can undergo nitrosation to 1,2,6-trinitrobenzaldoxime [35], which in turn can undergo a Beckmann fragmentation to 1,2,6-trinitrobenzonitrile or a cyclization-dehydration reaction to 4,6-dinitro-1,2-benzisoxazole (Fig 1). Benzaldoximes are susceptible to both photochemical and nonphotochemical Beckmann rearrangements to primary amides (Fig 1) [56–58]. 4,6-Dinitro-2,1-benzisoxazole results from a cyclization and dehydration reaction from 2-hydroxylamino-4,6-dinitrobenzaldehyde (Fig 1). As 4,6-dinitro-1,2-benzisoxazole, 2,4,6-trinitrobenzaldoxime, and 2,4,6-trinitrobenzonitrile originated from an initial photonitrosation reaction between TNT and nitrous acid at pH < 5, these compounds were not expected to form under environmental conditions [34]. However, more recent studies have demonstrated that photonitrosation reactions can occur up to pH 7.5 [59]. 4,6-Dinitroindazole is another example of a heterocyclic nitrogen compound formed as the result of a nitrosation reaction, in this case from 2ADNT (Fig 2) [60]. Phenolic compounds are also susceptible to nitrosation (Fig 2). Nitrosation of primary aromatic amines can lead to deamination reactions via diazonium ion intermediatates, as in the conversion of 3,5-dinitroaniline to 3,5-dinitrophenol (Fig 2).

There may be more than one pathway leading to particular transformation products. For example, 3,5-dinitrophenol also was considered to arise from photochemical decarboxylation of 2-hydroxy-4,6-dinitrobenzenecarboxylic acid [53]. In some cases, identified photolysis products of TNT have in turn been the subject of further photodegradation studies. Direct photolysis of 1,3-dinitrobenzene in aqueous solution produced hydroquinone, resorcinol, catechol, 4-nitrocatechol, nitrohydroquinone, 4-nitrophenol, 3-nitrophenol, 2-nitrophenol, and 2,4-dinitrophenol (Fig 2) [61]. Photodegradation of 2ADNT produced 4,6-dinitroindazole [37]. Limited information is available on the order of transformation reactions as a function of experimental conditions. The scheme for photochemical transformation of TNT to 1,3,5-trinitrobenzene, 2-amino-4,6-dinitrobenzoic acid, 3,5-dinitroaniline, 3,5-dinitrophenol, and 2,4-dinitrobenzoic acid proposed by Spanggord et al. is shown in S2 Fig [53].

Dimerization and polymerization reactions proceed through several mechanisms. Condensation between nitroso and hydroxylamino intermediates leads to azoxy compounds, and condensation between nitroso and aromatic amine intermediates to azo compounds (Fig 3). Considering the additional reduction step required from phenylhydroxylamine to aromatic amine, azoxy compounds presumably form ahead of azo compounds. Burlinson reported the photochemical conversion of 2-carboxy-3,3',5,5'-tetranitro-NNO-azoxy benzene to its corresponding azo compound (Fig 4) [34]. Coupling between diazonium ions and phenols is another possible route to azo compounds (Fig 4). Condensation between aromatic amines and benzaldehydes leads to imines (Schiff bases) (Fig 4). As catechols and hydroquinones are potential photolysis products that can oxidize to quinones, the 1,2- addition of aromatic amines with quinones to form imines, and the 1,4-addition of aromatic amines with quinones to form anilinohydroquinone and anilinoquinone adducts, may be considered plausible condensation reactions as well. Spanggord proposed that the initial development of color in solid TNT exposed to sunlight is due to formation of structures RP1 and RP2 (Fig 3) [23]. RP1 was postulated to result from condensation of two molecules of the 2,4,6-trinitrobenzyl anion via nucleophilic addition of one anion to the C_3_ carbon of a second anion; cyclization and dehydration to the isoxazole ring yields RP2. TNT is employed as a reagent in the synthesis of stilbene derivatives [60]. TNT condenses with benzaldehydes in the presence of secondary amines to form stilbene derivatives, via nucleophilic addition of the trinitrobenzyl anion to the aldehyde [60]. Treatment of TNT with bleach (NaOCl) results in the formation of 2,2’,4,4’,6,6’-hexanitrostilbene (Fig 4) [62]. Stilbene derivatives have not been considered as photochemical transformation products thus far.

Burlinson and Kaplan reported one amide photodegradation product, the secondary amide N-(2-carboxy-3,5-dinitrophenyl)- 2,4,6-trinitrobenzamide. Although a pathway was not specified, nitrones, which would arise from condensation of the phenylhydroxylamino and bezaldehyde derivatives of TNT, are known to undergo photochemical rearrangements to secondary amides via oxaziridine intermediates (Fig 3) [57, 63]. In the case of the α,N-diphenylnitrones originating from TNT, the secondary amides would be in the form of benzanilides. Thus, from the older literature according to Spence [63], benzanilide was formed along with other products from the sunlight exposure of α,N-diphenylnitrone [64], and benzanilide was formed from irradiation of a mixture of benzaldehyde and nitrobenzene [65]. The photochemical rearrangement of nitrones to amides has in fact been exploited for peptide synthesis [66]. Arylhydroxylamines are generally about an order of magnitude more nucleophilic than their corresponding arylamine analogs because of the alpha-effect [67]. Brighente concluded that the rate-determining step in nitrone formation between phenylhydroxylamine and various benzaldehydes was dehydration of the dihydroxy intermediate over the pH range of 1–11 [68]. Nitrone formation from TNT derived phenylhydroxylamines and benzaldehydes specifically has not been documented in the literature. For reasons of steric hindrance, the 4-hydroxylamino derivatives of TNT and 2,4-DNT presumably would be favored over the 2-hydroxylamino derivatives of TNT and the DNTs as nucleophiles. Also, due to steric hindrance around the aldehyde electrophile, condensation between the hydroxylamino nucleophiles and 2,4-dinitrobenzaldehyde or 2,6-dinitrobenzaldehyde would presumably be more probable than with 2,4,6-trinitrobenzaldehyde. Rate constants have been reported for nitrone formation between phenylhydroxylamine and 2,6-dichlorobenzaldehyde [69]. TNT itself is known to react with p-nitrosodialkylanilines to generate nitrones (Fig 4) [60].

Along with benzanilide formation, the reactions leading to 1° amide, nitrosophenol, aldimine, anilinohydroquinone, and anilinoquinone nitrogens have not previously been considered during TNT photolysis, but are implicated in the NMR analyses that follow.

Photolysis of the nitrite and nitrate released from TNT produces reactive species that have the potential to effect further transformation reactions of the TNT and its degradation products, but their roles have not been completely defined. Photolysis of nitrite yields nitric oxide, nitrogen dioxide, and hydroxyl radical (Fig 5), and occurs under sunlight irradiation and under both unfiltered and Pyrex-filtered irradiation from the medium pressure mercury lamp [70, 71]. Photolysis of nitrate (Fig 5) yields nitrogen dioxide, nitrite, peroxynitrite, and hydroxyl radical, and occurs under unfiltered irradiation from the medium pressure mercury lamp [71, 72]. Nitrite (absorption maximum near 354 nm) undergoes photolysis primarily to nitric oxide and hydroxyl radical after absorption of light in the 300- to 400-nm wavelength region; photolysis of nitrite to nitrogen dioxide and hydrated electron is a minor parallel reaction [70]. Nitrate has a weak absorption band at ~302 nm and a strong absorption band at ~200 nm. Excitation in the weak band leads to formation of nitrite with a low quantum yield (ɸ = 0.08; Fig 5, reaction 5) [72]. Excitation in the strong band proceeds through reactions 3 and 4 (Fig 5) with formation of nitrogen dioxide, hydroxyl radical, and peroxynitrite, the latter with a significantly higher quantum yield of ɸ = 0.48 [72]. The main contribution to nitrite formation at ʎ< 280 nm is through decomposition of peroxynitrite [72]. Thus nitrite and nitrate photolysis may effect nitrosation, nitration, hydroxylation, and redox reactions.

**Fig 6:**
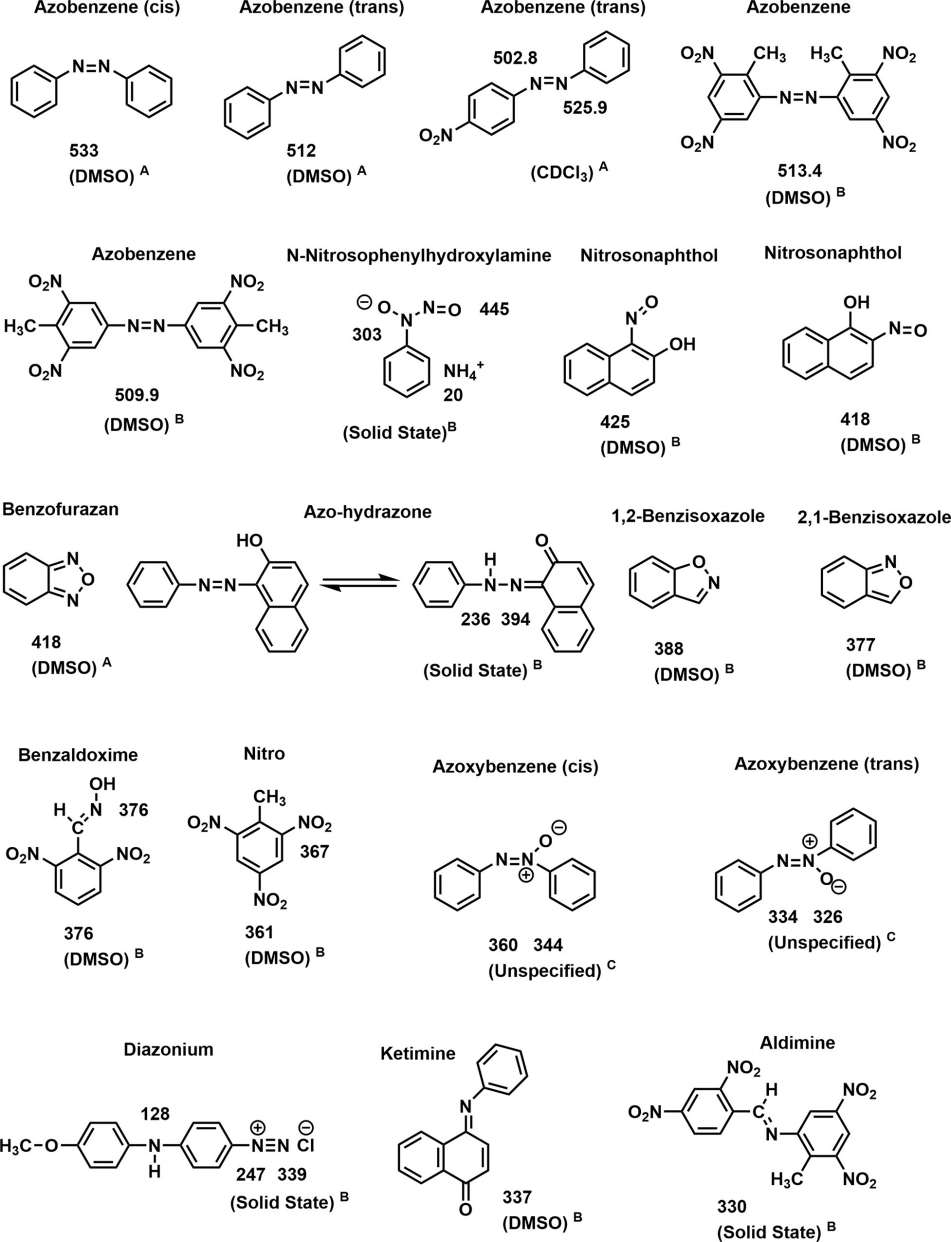
N-15 NMR chemical shifts of nitrogen compounds. A: Ref 73 & 76; B: Determined in this laboratory; C: Ref 75; D: Ref 74.

**Fig 7:**
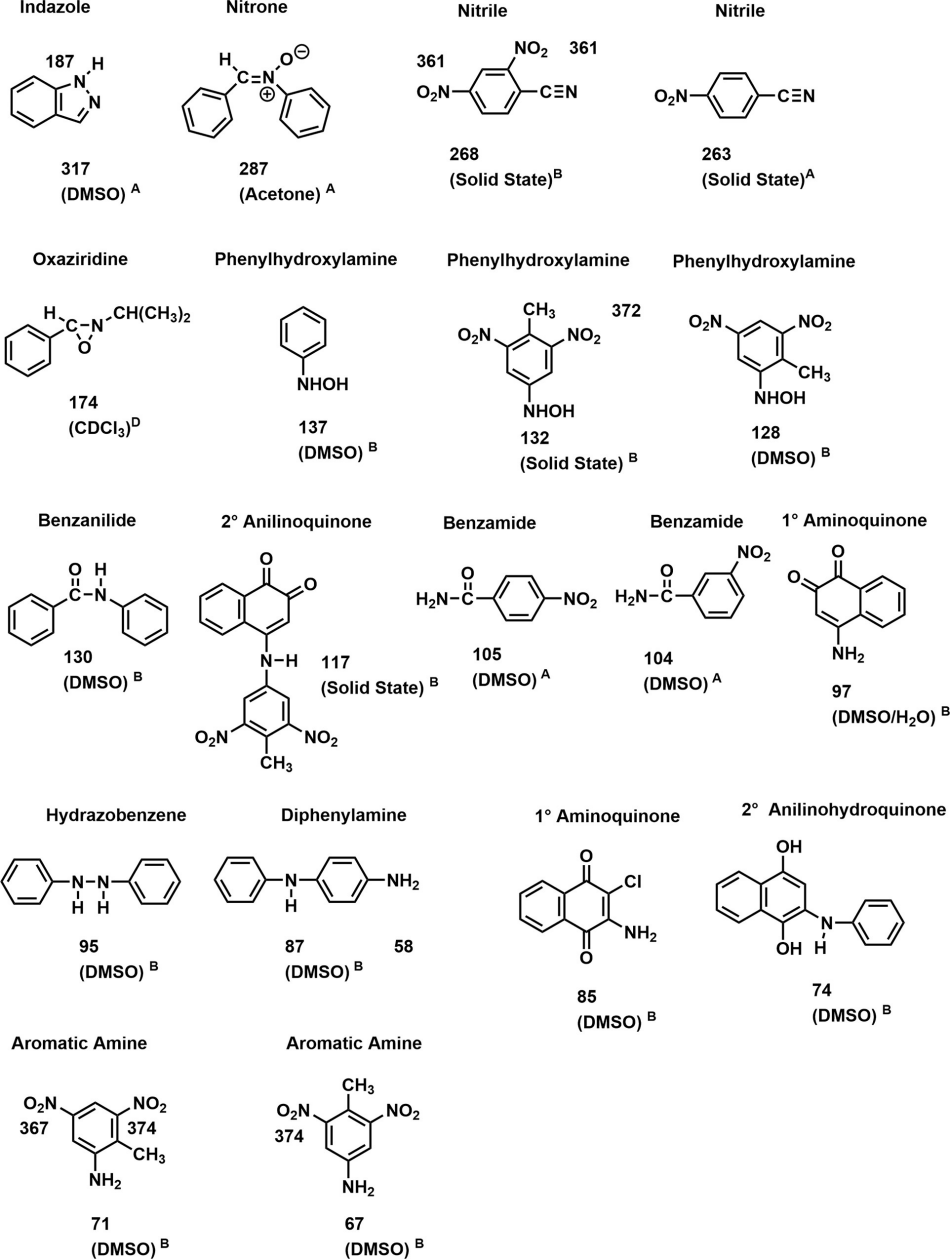
N-15 NMR chemical shifts of nitrogen compounds. A: Ref 73 & 76; B: Determined in this laboratory; C: Ref 75; D: Ref 74.

**Fig 8:**
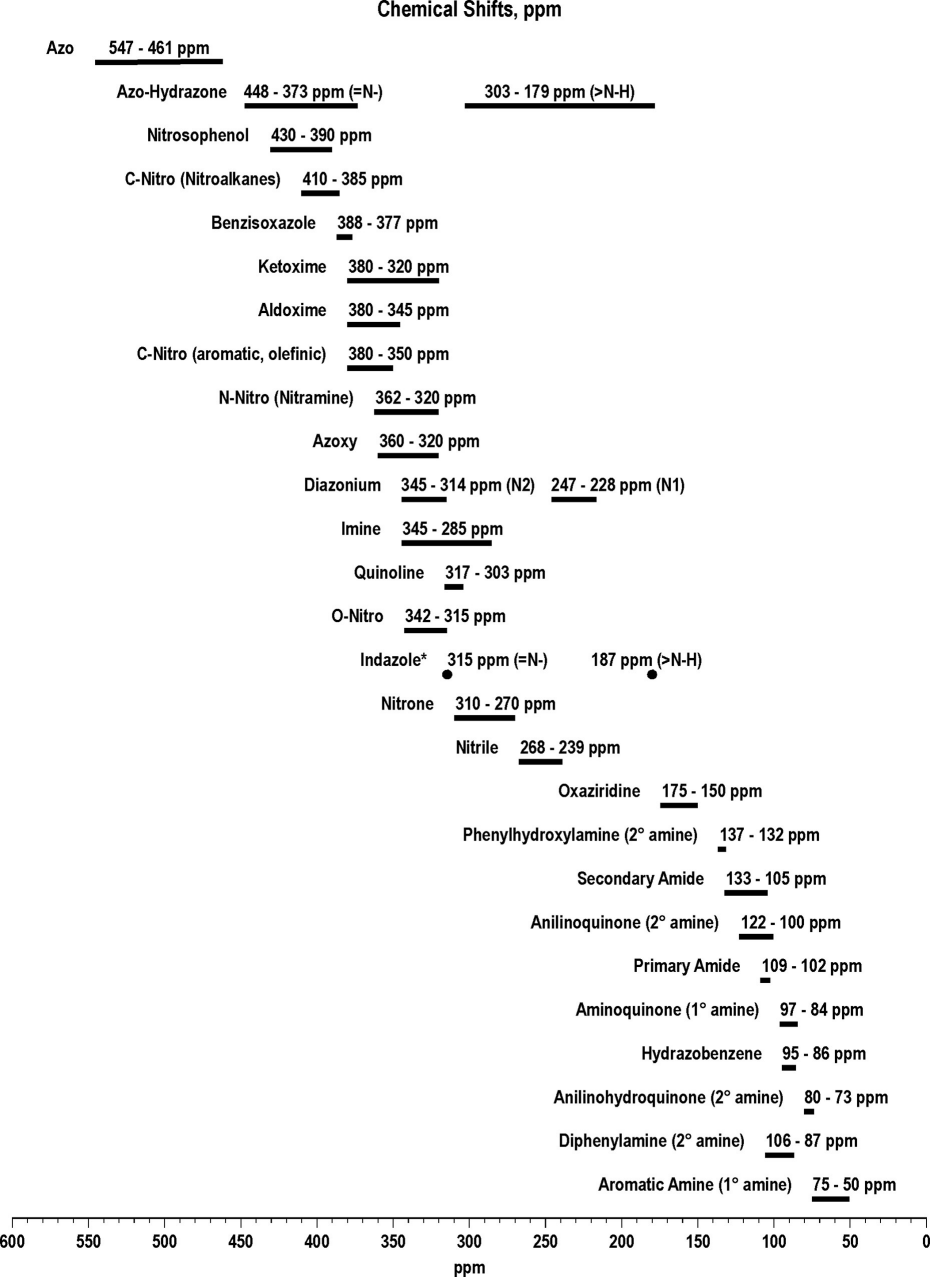
N-15 NMR chemical shift ranges of nitrogen functional groups. * Based on shifts for a single indazole structure.

Nitrogen-15 NMR chemical shifts of functional groups representing potential transformation products, determined in this laboratory or taken from the literature [73–76], are illustrated in Figs 6–8.

### Objectives

We previously reported liquid and solid state ^13^C and ^15^N NMR spectra of the photolysate from a saturated solution of ^15^N-labeled TNT in deionized water subjected to irradiation from an unfiltered medium pressure mercury lamp [22]. Here we extend the analyses to photolysates of the labeled TNT from Pyrex-filtered lamp irradiation in deionized water, pond water (pH = 8.3), pH 8 buffer solution, and in aqueous solution in the presence of Suwannee River NOM, with the deionized water and pond water experiments compared to natural sunlight irradiation. These are compared to the photolysate of solid TNT exposed to natural sunlight. Pyrex-filtered lamp irradiations of 2-amino-^15^N-4,6-dinitrotoluene (2ADNT) and 4-amino-^15^N-2,6-dinitrotoluene (4ADNT) in deionized water, and both Pyrex-filtered and unfiltered irradiations of unlabeled 2,6-dinitrotoluene in deionized water are also examined. The objective is to determine the classes and relative quantities of functional groups in the total photodegradation product mixtures, and to identify future research needs, in terms of what compounds may be targeted for specific structural confirmation through chromatographic separation in conjunction with further NMR and mass spectrometric analyses, and which reaction pathways need further elaboration. The comparison of liquid and solid state analyses illustrates the advantages and limitations of each technique in addressing these future research goals. NMR in general offers the advantage of direct analysis of the whole product mixture, while ^15^N NMR in particular is ideally suited to following transformation of the nitro groups of TNT during photolysis.

## Materials and methods

### Materials

2,4,6-Trinitrotoluene-^15^N_3_, 99 atom percent ^15^N, was purchased from ISOTEC (St. Louis, MO). The labeled compounds 2-amino-^15^N-4,6-dinitrotoluene (2ADNT) and 4-amino ^15^N-2,6-dinitrotoluene (4ADNT), 99 atom percent ^15^N, were prepared by Dr. Ronald Spanggord, SRI International, Menlo Park, CA. Unlabeled 2,4,6-trinitrotoluene, 99% purity, was purchased from Chem Service, Inc. (West Chester, PA). 2,6-Dinitrotoluene was purchased from Sigma-Aldrich. 2,4,6-Trinitrobenzaldehyde was purchased from Creative Dynamics Inc., Shirley, NY. 2-Amino-4,6-dinitrobenzoic acid and 4-amino-2,6-dinitrobenzoic acid were purchased from Ryan Scientific, Mount Pleasant, South Carolina. 2,4-Dinitrobenzyl alcohol was purchased from Aldlab Chemicals, West Newton, MA. Other compounds employed for NMR chemical shift determination were purchased from Sigma-Aldrich or Chem Service, Inc. 2,6-Dinitrobenzaldehyde oxime was prepared from reaction of 2,6-dinitrobenzaldehyde with ^15^NH_2_OH.HCl (Isotec) in methanol. 2-Amino-^15^N-4,6-dinitrotoluene was reacted with 2,4-dinitrobenzaldehyde in methanol to form the aldimine (N-(2,4-dinitrobenzilidene)-2-methyl-3,5-dinitroaniline). 2,2’-Dimethyl-3,3’,5,5’-tetranitroazobenzene and 4,4’-dimethyl-3,3’,5,5’-tetranitroazobenzene were obtained as side products in the Caro acid (peroxymonosulfuric acid) oxidation of 2-amino-^15^N-4,6-dinitrotoluene and 4-amino ^15^N-2,6-dinitrotoluene, respectively. 2-Hydroxylamino-4,6-dinitrotoluene was also obtained from the Caro acid oxidation of 2-amino-^15^N-4,6-dinitrotoluene. Suwannee River NOM (SRNOM;1R101N) was purchased from the International Humic Substances Society.

Pond water was collected on June 20, 2007 from the Gravel Pond on the south side of Chatfield Reservoir, located south of Littleton, Colorado. Water was passed sequentially through PALL Glass Fiber Type A/E 1μm and 0.45μm silver filters. The filtered water was refrigerated prior to irradiation experiments. Conductivity, pH, and DOC were 630 μS, 8.3, and 3.0 mg C/L, respectively. Trace metal analyses were performed by Huffman Laboratories, Golden, CO. Iron was less than 0.05mg/L and Cr, Co, Cu, and Mn all less than 0.01 mg/L.

### Irradiations

#### Aqueous-phase irradiations

Aqueous-phase irradiations were performed on approximately saturated solutions of TNT, 2ADNT, 4ADNT, and 2,6-DNT. The photochemical reactor (Ace Glass Inc., Vineland, NJ; Part No. 7840–185) consisted of a 450 Watt medium pressure mercury-vapor lamp, housed in a quartz immersion well with a Pyrex filter, and equipped with a 1.0 liter reaction vessel. The three ports to the reaction vessel were kept open to the atmosphere to maintain oxic conditions.

#### Lamp irradiation of T^15^NT in deionized water

Solutions of 100 mg of T^15^NT dissolved in 0.9 L deionized water (4.90 X 10^-4^M) were irradiated for periods of 1 and 4 hours, with the initial pH of 5.5 in the former case dropping to 3.5 after the 1 hour irradiation. A solution of unlabeled TNT at the same concentration was also subjected to the 1 hour irradiation and then centrifuged to collect a precipitate fraction. Irradiations were also performed for 1 and 16 hour periods without the Pyrex filter. Solutions for all experiments were freeze dried after irradiation.

In a replicate experiment of the unlabeled TNT in deionized water (4.90 X 10^-4^M) irradiated for 1 hour with the Pyrex-filtered lamp, the mass recovery was 93%.

#### Sunlight irradiation of T^15^NT in deionized water

A solution of 100 mg of T^15^NT dissolved in deionized water (4.35 x 10^−4^ M) was transferred to a 20 cm x 20 cm 1.8 L Pyrex baking dish, fitted with a quartz lid, and exposed to sunlight between approximately 9:30 AM and 7:30 PM on 6/10/2006 and 6/11/2006 in Denver, Colorado.

#### Lamp irradiation of 2ADNT and 4ADNT in deionized water

Individual solutions of 41 mg of 2-amino-^15^N-4,6-dinitrotoluene and 45 mg of 4-amino-^15^N-2,6-dinitrotoluene in 0.9 L deionized water were irradiated with the Pyrex–filtered lamp for 1 and 2 hours, respectively. The initial pH of 7.5 for the 2ADNT solution dropped to 7.4 after irradiation.

#### Sunlight and lamp irradiation of T^15^NT in pond water

A solution of 100 mg of T^15^NT dissolved in 1 L of pond water (4.35 x 10^−4^ M) was exposed to sunlight in the quartz covered baking dish between approximately 11:00 AM and 7:30 PM on 6/23/2007 in Denver, Colorado. The solution was prepared the evening prior and kept refrigerated in a brown glass bottle. At the time of transfer to the vessel, the solution was completely clear, showing no evidence of visible color as a result of hydrolysis. The solution turned color instantaneously upon exposure to the sunlight, becoming cherry red within one half hour. The solution was refrigerated overnight in the glass brown bottle and re-exposed for a second day between 11:00 AM and 7:30 PM on 6/24/2007. After solid state NMR analysis, the freeze dried powder was slurried in dimethysulfoxide then filtered to remove pond water dissolved solids that concentrated during freeze drying, in preparation for liquid state NMR analysis. A solution of 100 mg of T^15^NT dissolved in in 0.9 L pond water was also irradiated with the Pyrex–filtered lamp for 1 hour, with the initial pH of 7.9 dropping to 6.3.

#### Lamp irradiation of T^15^NT in presence of SRNOM

A solution of 100 mg T^15^NT dissolved in 0.9 L deionized water was amended with 51.3 mg of SRNOM (SRNOM DOC = 28 mg C/L) and irradiated with the Pyrex–filtered lamp for 1 hour. The initial pH of 3.7 dropped to 3.2.

#### Lamp irradiation of T^15^NT in pH 8 buffer solution

Individual solutions of 100 mg T^15^NT dissolved in 0.9 L pH 8 phosphate buffer were irradiated for periods of 1 and 4 hours with the Pyrex–filtered lamp. The buffer maintained the solutions at pH 8 for the duration of the irradiations. The solutions were freeze dried for solid state NMR analysis without separation of the transformation products from the buffer salts.

#### Lamp irradiation of 2,6-dinitrotoluene in deionized water

A solution of 212 mg of 2,6-DNT in 0.9 L deionized water was irradiated with the unfiltered lamp for 1 hour, and a solution of 162 mg of 2,6-DNT in 0.9 L deionized water was irradiated with the Pyrex-filtered lamp for 4 hours. In the latter experiment, the initial pH of 4.4 dropped to 3.2 after irradiation.

#### Solid-phase irradiation: Sunlight irradiation of solid T^15^NT

For the solid phase irradiation, 150 mg of T^15^NT, finely ground in a mortar and pestle, was placed in a 6.5 inch diameter Kimax glass petri dish, set in the baking dish fitted with a quartz cover, and exposed to sunlight over a period of 10 days from 4/20/2012 to 4/30/2012 in Denver, Colorado, where the average relative humidity over the 10 day period was 32%. The T^15^NT became discolored upon initial exposure and had turned an orange brown color by the end of 10 days. The sample was packed into a rotor for solid state NMR analysis. A film of sublimed T^15^NT formed on the quartz cover. This material was recovered with dimethylsulfoxide. Liquid state NMR analysis was performed on the combined bulk sample and sublimated material dissolved in dmso-d_6_.

### Weak alkaline hydrolysis dark control

For the dark control of weak alkaline hydrolysis, 100 ml of T^15^NT was dissolved in 1 L deionized water and titrated to pH 8.2 with NaHCO_3_, adjusted to pH 9.0 with NaOH, and allowed to stir in a vessel covered with aluminum foil for 24 hours, at which point the pH had dropped to 7.6. The pH was adjusted to 8.3 with NaOH and allowed to stir covered an additional 6 days. The solution with final pH of 7.7 was freeze dried.

### NMR spectroscopy

Solid state constant amplitude CP/MAS (cross polarization/magic angle spinning) ^13^C and ^15^N NMR spectra were recorded on a Chemagnetics CMX-200 NMR spectrometer at carbon and nitrogen resonant frequencies of 50.3 and 20.3 MHz, respectively, using a 7.5 mm ceramic probe (zirconium pencil rotors). Acquisition parameters for ^13^C included a 30,030.0 Hz (597 ppm) spectral window, 17.051-ms acquisition time, 2.0-ms contact time, 0.5-s pulse delay, and spinning rate of 6 KHz. Acquisition parameters for ^15^N included a 26,666.7 Hz (1314 ppm) spectral window, 19.201-ms acquisition time, 1.0-ms or 2.0-ms contact time, 0.5-s pulse delay, and spinning rate of 5 or 6 KHz. Nitrogen-15 chemical shifts were referenced to glycine, taken as 32.6 ppm, and reported on the ammonia scale.

Liquid phase NMR spectra were recorded on a Varian GEMINI 2300 NMR spectrometer at carbon and nitrogen resonant frequencies of 75.4 and 30.4 MHz respectively using a 10 mm broadband probe. The NMR solvent was dimethylsulfoxide-d_6_ (dmso-d_6_) in all cases. Acquisition parameters for ^13^C included a 30,000 Hz (398 ppm) spectral window. 45° pulse width, 0.5-s acquisition time, 1.0-s pulse delay, and continuous decoupling. ACOUSTIC ^15^N NMR spectra (alternating compound one eighties used to suppress transients in the coil) [77] were recorded using a 35,111.7 Hz (1154 ppm) spectral window, 0.5-s acquisition time, 1.0-s pulse delay, and tau delay of 0.1-ms. Continuous decoupled ^15^N NMR spectra were recorded using a 35,111.7 Hz spectral window, 45° pulse angle, 0.5-s acquisition time, and 1.0-s pulse delay. DEPT ^15^N NMR spectra (distortionless enhancement by polarization transfer) were recorded with a 26,000 Hz (855 ppm) spectral window, 0.5-s acquisition time, 1.0-s delay for proton relaxation, and ^1^J_NH_ of 90.0 Hz. Inverse gated decoupled (IGD) ^15^N NMR spectra were acquired using a 35,111.7 Hz (1,154.3 ppm) spectral window, 0.5-s acquisition time, and 2.0-s pulse delay, with the addition of 0.09 M Cr(Acac)_3_ (chromium acetylacetonate) as paramagnetic relaxation agent. Neat formamide (112.4 ppm) was used as an external reference standard. The ^15^N NMR chemical shifts are reported in ppm downfield of ammonia, taken as 0.0 ppm.

Three sets of pulse sequences were typically recorded on samples during liquid state ^15^N NMR analysis: IGD, DEPT, and ACOUSTIC or continuous decoupling. The ACOUSTIC pulse sequence alleviates baseline role from acoustic ringing in the probe. Quantitative distributions of nitrogens can be obtained by employing inverse gated decoupling to eliminate NOE (nuclear Overhauser enhancement) and pulse delays sufficient to allow for complete relaxation of ^15^N spin lattice relaxation times, T_1_’s, achieved here with a 2.0-sec pulse delay in conjunction with 0.09 M Cr(Acac)_3_. The NOE is retained in the ACOUSTIC and continuous decoupling pulse sequences. No one pulse sequence provides all the information available from ^15^N NMR analysis. As will be shown further on, for example, some sp^3^ hybridized nitrogens bonded to protons are not observed in polarization transfer spectra due to exchange, and are most readily detected through retention of the NOE in the ACOUSTIC or continuous decoupling pulse sequences. In comparing the aromatic amine regions of the spectra, the signal enhancement from NOE proved advantageous. The IGD spectrum of the photolysate from sunlight irradiation in the solid state was hampered by acoustic ringing and is not shown.

## Results

### Irradiation of T^15^NT in deionized water

#### Liquid state ^13^C NMR

**Fig 9:**
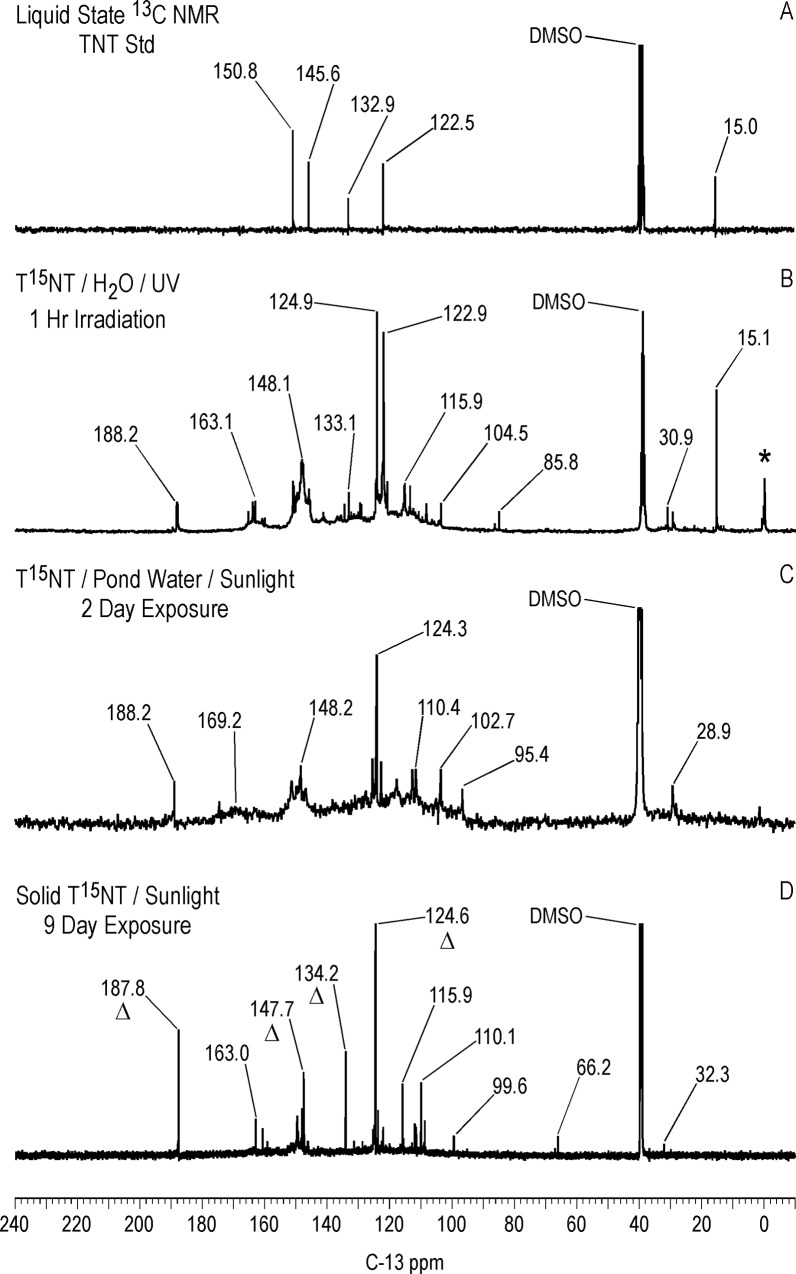
Liquid state continuous decoupled C-13 NMR spectra of T^15^NT. A. Standard. B. Lamp irradiation in deionized water. C. Sunlight irradiation in pond water. D. Sunlight irradiation in the solid state. Line broadening = 1.0 Hz. Triangles in spectrum D denote peaks corresponding to 2,4,6-trinitrobenzaldehyde.

The ^13^C NMR assignments for the TNT standard in dmso-d_6_ are: C_1_ = 132.9 ppm; C_2,6_ = 150.8 ppm; C_4_ = 145.6 ppm; C_3,5_ = 122.5 ppm; methyl = 15.0 ppm (Fig 9A). The liquid state ^13^C NMR spectrum of T^15^NT irradiated in deionized water for one hour is shown in Fig 9B. The large number of peaks in the spectrum of the photolysate indicates a multicomponent product mixture. The methyl and C_3,5_ peaks corresponding to the parent T^15^NT are still visible while the C_1_, C_2,6_, and C_4_ carbons overlap with the degradation product peaks. Notable features are the carboxylic acid and amide carbons from approximately 158 to 168 ppm, and the protonated aromatic carbons from approximately 100 to 118 ppm, upfield of the C_3,5_ peak at 122.5 ppm. The peak at 188.2 ppm corresponds to the aldehyde carbon of 2,4,6-trinitrobenzaldehyde (Table 2), an early stage photo-oxidation product of the TNT. Within the carboxylic acid and amide carbon region, from an expanded plot (S3 Fig), peaks visible at 160.9, 162.2, 163.9, and 165.3 ppm are possible matches for bicarbonate, 2,4,6-trinitrobenzoic acid, 4-amino-2,6-dinitrobenzoic acid, and 2-amino-4,6-dinitrobenzoic acid, respectively (Table 2). Interestingly, there is no evidence for the 2,4,6-trinitrobenzyl alcohol carbon, which occurs at about 55.5 ppm (Table 2). Its absence suggests that the alcohol undergoes a facile oxidation to the more stable aldehyde and carboxylic acid derivatives. Alternatively, it is present in the photolysate mixture, but below the detection limit of ^13^C NMR under the acquisition condition employed. Which compound or compounds are responsible for the initial color formation of TNT exposed to sunlight is one of the long running questions in TNT photolysis. The reddish-pink color of the 2,4,6-trinitrobenzaldehyde standard should place it under consideration as one of the initial colored products. A few low intensity peaks occur in the aliphatic region at 29.1, 30.9, 85.8 and 87.1 ppm. The assignments have not been confirmed, but the peaks at 29.1–30.9 ppm would be consistent with the predicted chemical shift for the methylene carbon to structure RP2 (Fig 3).

**Table 2:** C-13 NMR chemical shifts of carbonyl and alcohol carbons^a^.

Compound	^13^C Chemical Shift of Carbonyl or Alcohol	Solvent
2,6-dinitrobenzaldehyde	188.9	DMSO-d_6_
2,4,6-trinitrobenzaldehyde	187.9	DMSO-d_6_
2,4-dinitrobenzaldehyde	187.3	Solid State
4-nitroanthranilic acid (2-amino-4-nitrobenzoic acid)	168.3	DMSO-d_6_
2-amino-4,6-dinitrobenzoic acid	165.3	DMSO-d_6_
2,4-dinitrobenzoic acid	164.9	DMSO-d_6_
N-(2-carboxy-3,5-dinitrophenyl)-2,4,6-trinitrobenzamide	164.7	Predicted ^b^
4-amino-2,6-dinitrobenzoic acid	163.9	DMSO-d_6_
2,6-dinitrobenzoic acid	163.4	DMSO-d_6_
2,4,6-trinitrobenzoic acid	162.2	DMSO-d_6_
Sodium bicarbonate	161.1	DMSO-d_6_
2,4-dinitrobenzylic alcohol	61.8	Solid State
2,4,6-trinitrobenzylic alcohol	55.5	Predicted ^b^
2,6-dinitrobenzylic alcohol	55.5	Predicted ^b^

^a^ Determined in this laboratory.

^b^ Predicted from ChemDraw.

DEPT ^13^C NMR spectra (not shown) confirmed these as methylene carbons. In support of this assignment is the observation of peaks at ~ 31 ppm in the spectrum of the weak alkaline hydrolysate of TNT discussed further on, which would be consistent with the adduct from nucleophilic addition of the 2,4,6-trinitrobenzyl anion to the C_3_ carbon of a second molecule of TNT or derivative. An alternative assignment for the peaks at 29.1–30.9 ppm in Fig 9B would be methylene carbons in bibenzyl (1,2-diphenylethane) structures resulting from reduction of stilbene derivatives of TNT. Complete assignments for the peaks from 100 to 118 ppm are beyond the scope of this study. However, a number of the transformation products listed in S1 Table contain resonances in this chemical shift range. For example, the chemical shifts to the C_1_, C_3_, and C_5_ carbons of 2-amino-4,6-dinitrobenzoic acid in dmso-d_6_ are 114.2, 111.3, and 104.2 ppm, respectively. The peak at 104.5 ppm in Fig 9B is a possible match to C_5_ of 2-amino-4,6-dinitrotoluene or 2-amino-4,6-dinitrobenzoic acid (S1 Table).

#### Solid state ^13^C NMR

**Fig 10:**
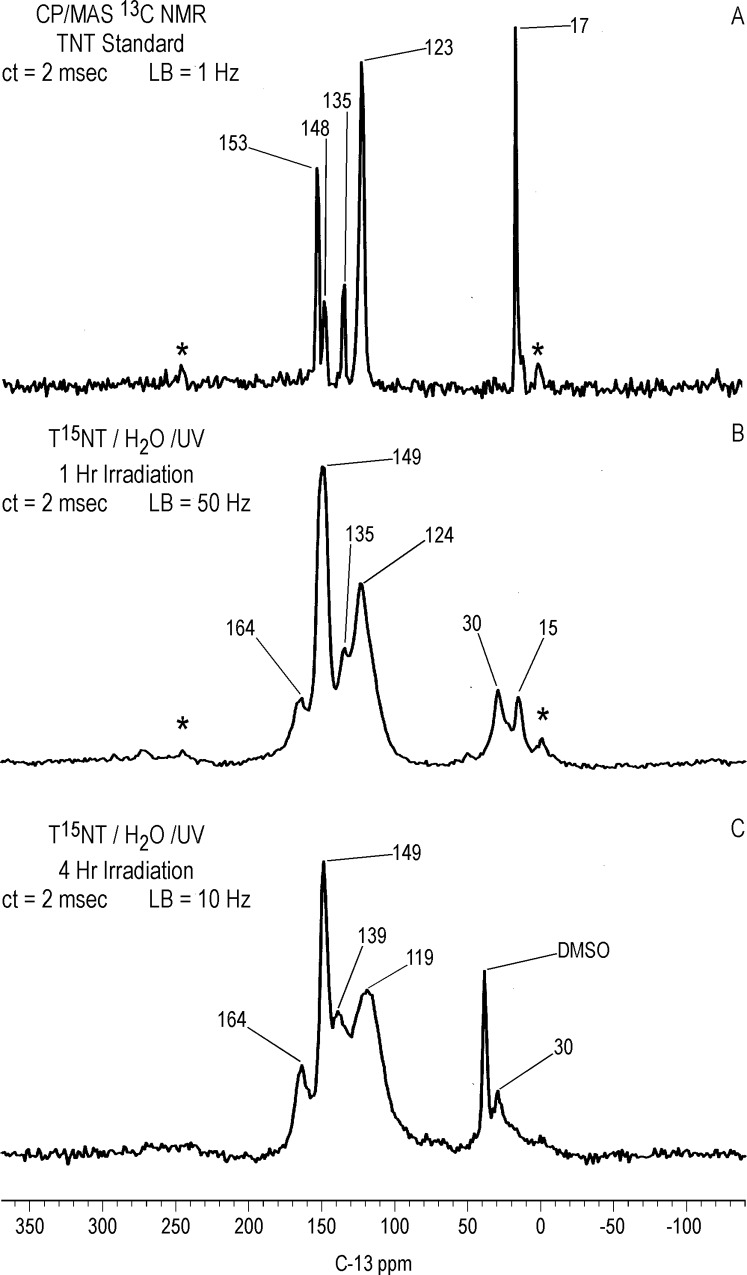
Solid state CP/MAS C-13 NMR spectra of T^15^NT. A. Standard. B. 1 hour lamp irradiation in deionized water. C. 4 hour lamp irradiation in deionized water. ct = contact time. LB = line broadening. Spinning Speed = 6 KHz. Asterisk = spinning side band.

Differing slightly from the liquid state, the ^13^C chemical shifts of the T^15^NT standard in the solid state are: C_1_ = 135 ppm; C_2,6_ = 153 ppm; C_4_ = 148 ppm; C_3,5_ = 123 ppm; methyl = 17.0 ppm (Fig 10A). On comparison to Fig 9B, the CP/MAS ^13^C NMR spectrum of the 1 hour photolysate (Fig 10B) illustrates the difference in resolution attainable between the liquid and solid state. No further resolution became apparent in the solid state spectrum when a narrower line broadening (1.0 Hz) was applied to the FID. The spectrum of the 1 hour photolysate exhibits broad bands at 15, 124, 135, and 149 ppm originating from the methyl and aromatic carbons of the parent TNT. The carboxyl and amide carbons are visible at 164 ppm. The discreet benzaldehyde peak at 188.2 ppm in the liquid spectrum is not detected in the solid spectrum. A notable feature of the solid state spectrum is the peak at 30 ppm, significantly enhanced over the discreet peaks at 30.9 and 29.1 ppm in the liquid state spectrum. This signal enhancement of the peak at 30 ppm in the CP experiment is replicated throughout the solid state ^13^C analyses.

After 4 hours of irradiation, the residual methyl carbons (15–17 ppm) have disappeared, concomitant with a relative increase in the intensity of the carboxyl/amide carbons at 164 ppm (Fig 10C). The methylene carbons at 30 ppm persist. These observations are consistent with the methyl groups having undergone further oxidation reactions or participation in nucleophilic addition reactions.

The methylene carbons at approximately 30 ppm are also present in significant concentrations in CP/MAS ^13^C NMR spectra of the photolysate from 1 hour unfiltered irradiation and the precipitate fraction from 1 hour of Pyrex-filtered irradiation of TNT in deionized water (S4 Fig). Their presence in the latter case is consistent with the assignment as bridging carbons in dimeric or oligomeric transformation products. Initial attempts at further investigation of these peaks through liquid state ^1^H-^13^C gHSQC (gradient heteronuclear single quantum coherence) analyses on a 600 MHz NMR spectrometer were unsuccessful.

#### Liquid state ^15^N NMR

**Fig 11:**
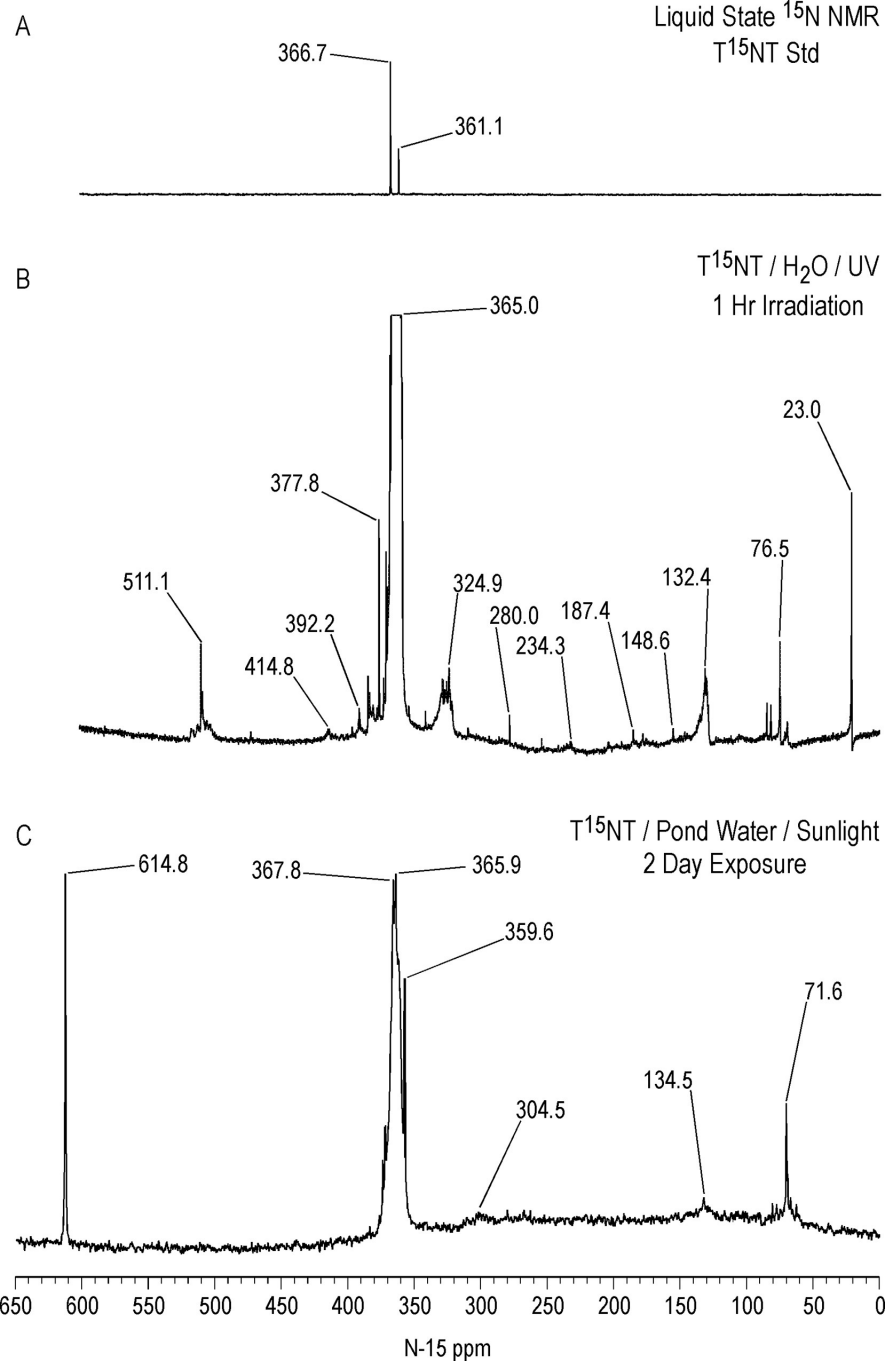
Liquid state IGD N-15 NMR spectra of T^15^NT. A. Standard. B. Lamp irradiation in deionized water. C. Sunlight irradiation in pond water. Line broadening = 3.0 Hz.

**Fig 12:**
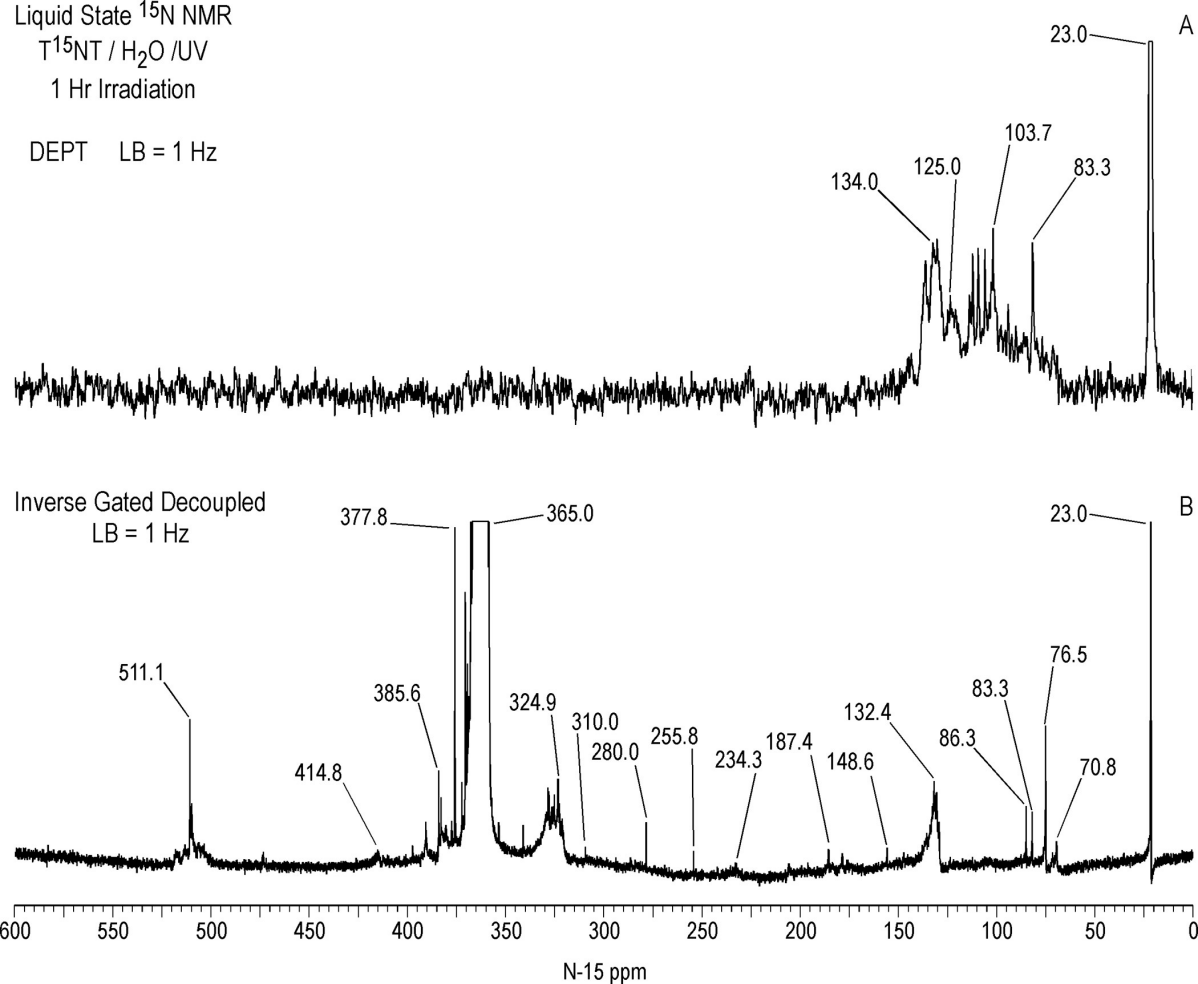
Liquid state N-15 NMR spectra of T^15^NT subjected to lamp irradiation in deionized water. A. DEPT spectrum showing all nitrogens bonded to protons in positive phase. B. IGD spectrum. LB = line broadening.

**Fig 13:**
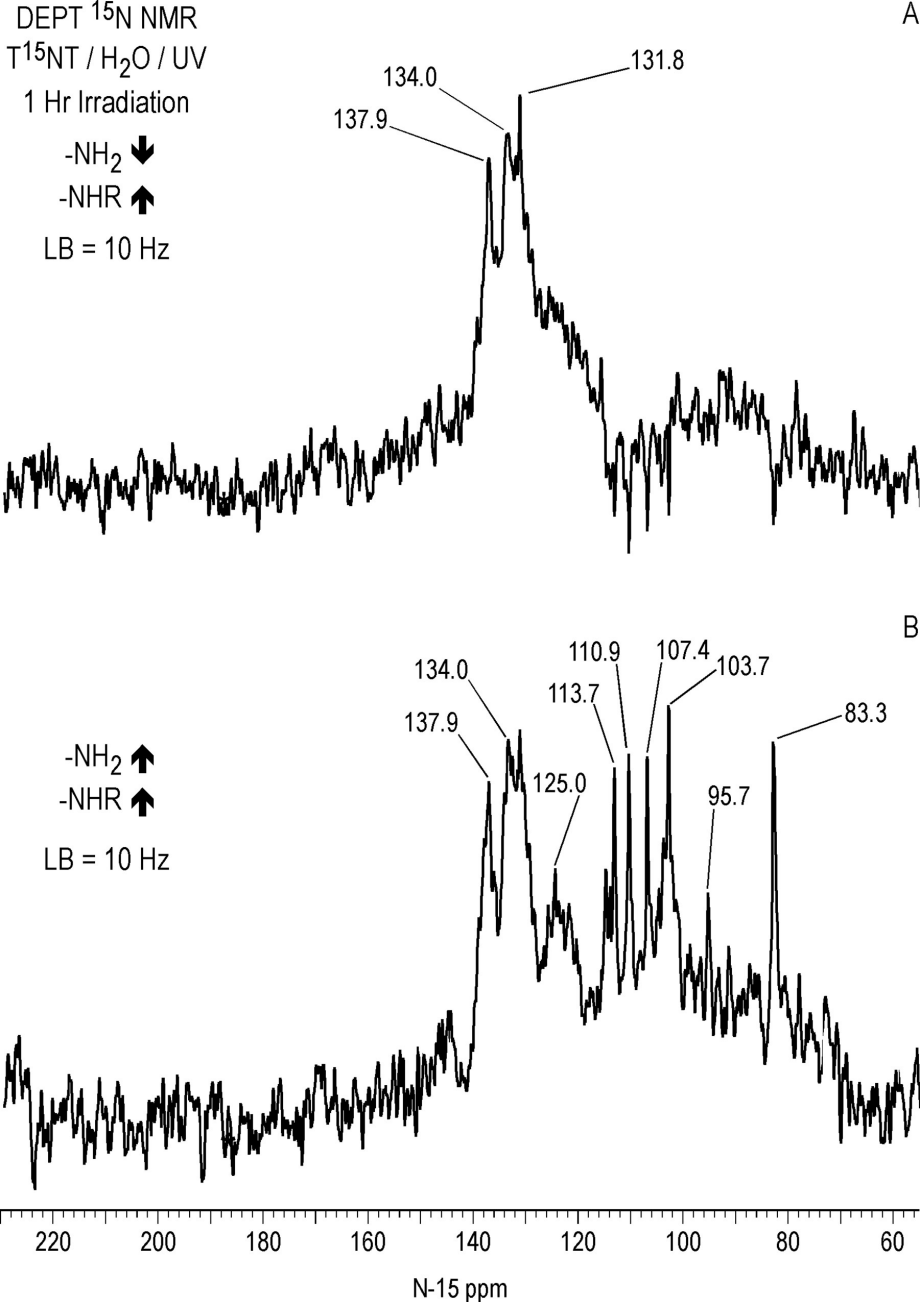
Liquid state N-15 NMR DEPT spectra of T^15^NT subjected to lamp irradiation in deionized water. A. Nitrogens bonded to two protons in negative phase and nitrogens bonded to one nitrogen in positive phase. B. All nitrogens bonded to protons in positive phase. LB = line broadening.

**Fig 14:**
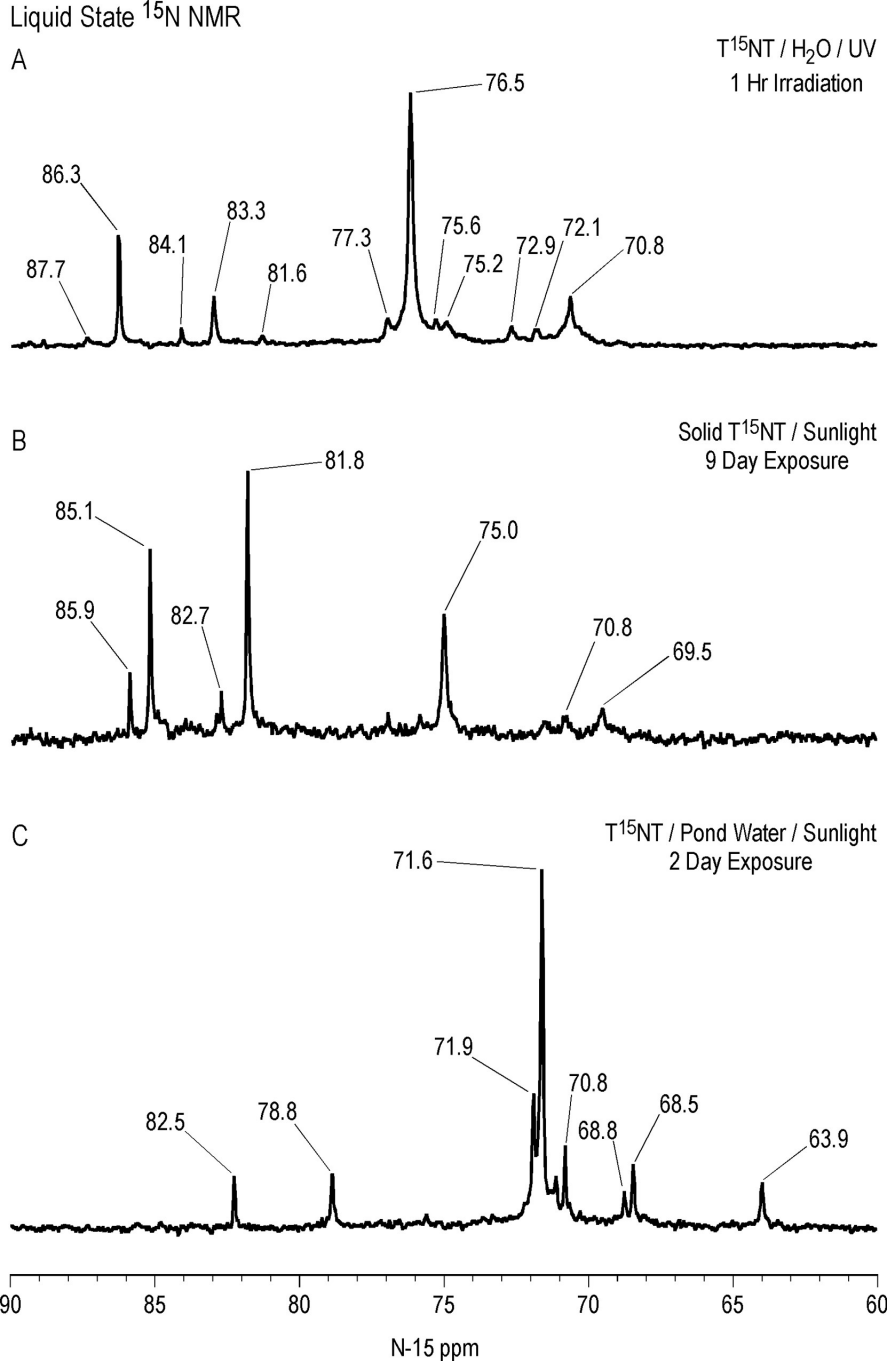
Liquid state ACOUSTIC N-15 NMR spectra of aromatic amine region of irradiated T^15^NT. Line broadening = 1 Hz. Spectra phased 180°. A. 1 hour lamp irradiation in deionized water. B. 9 day sunlight irradiation in the solid state. C. 2 day sunlight irradiation in pond water.

**Fig 15:**
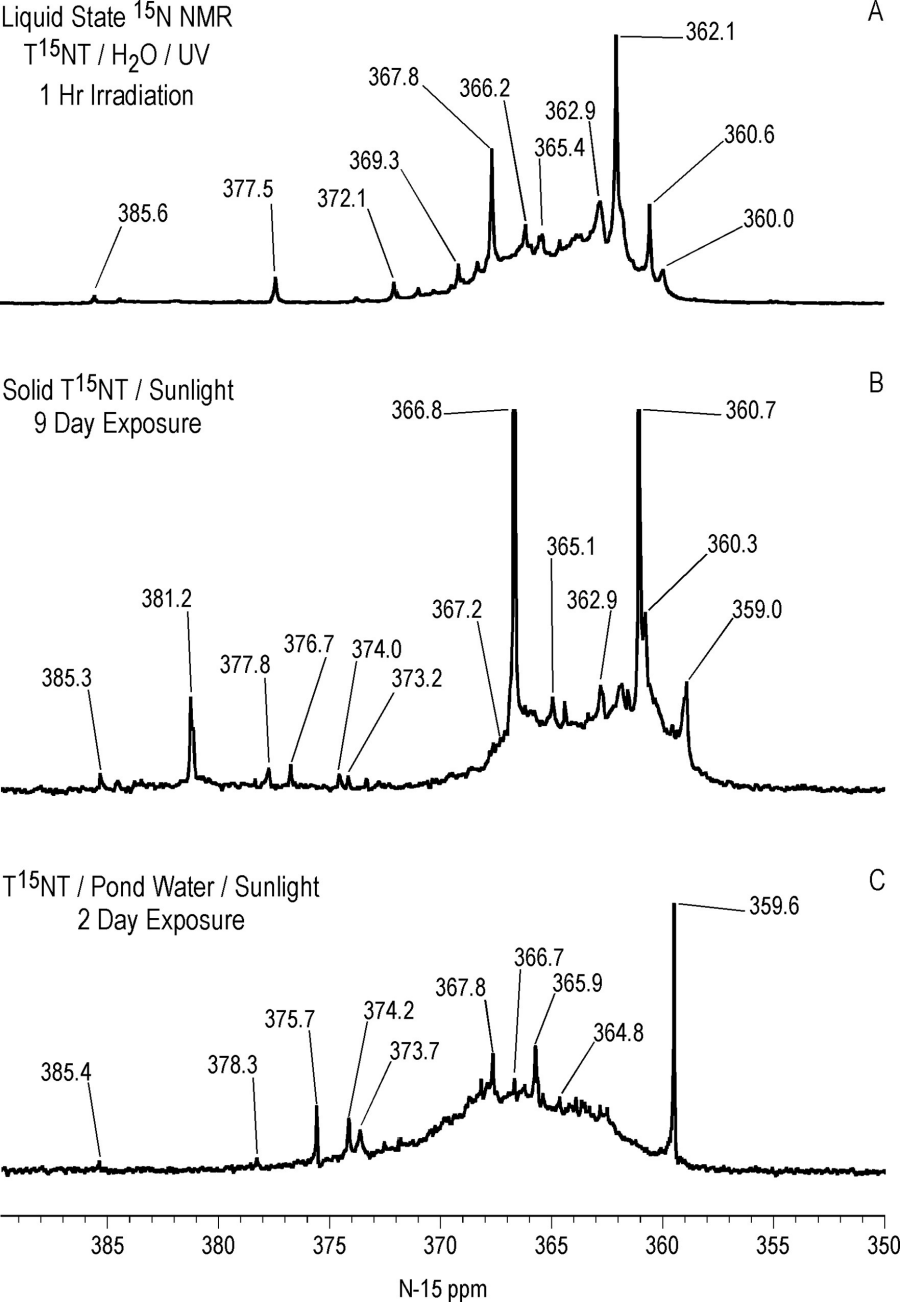
Liquid state N-15 NMR spectra of nitro region of irradiated T^15^NT. Line broadening = 1.0 Hz. A. 1 hour lamp irradiation in deionized water, IGD spectrum. B. 9 day sunlight irradiation in the solid state, ACOUSTIC spectrum. C. 2 day sunlight irradiation in pond water, IGD spectrum.

**Fig 16:**
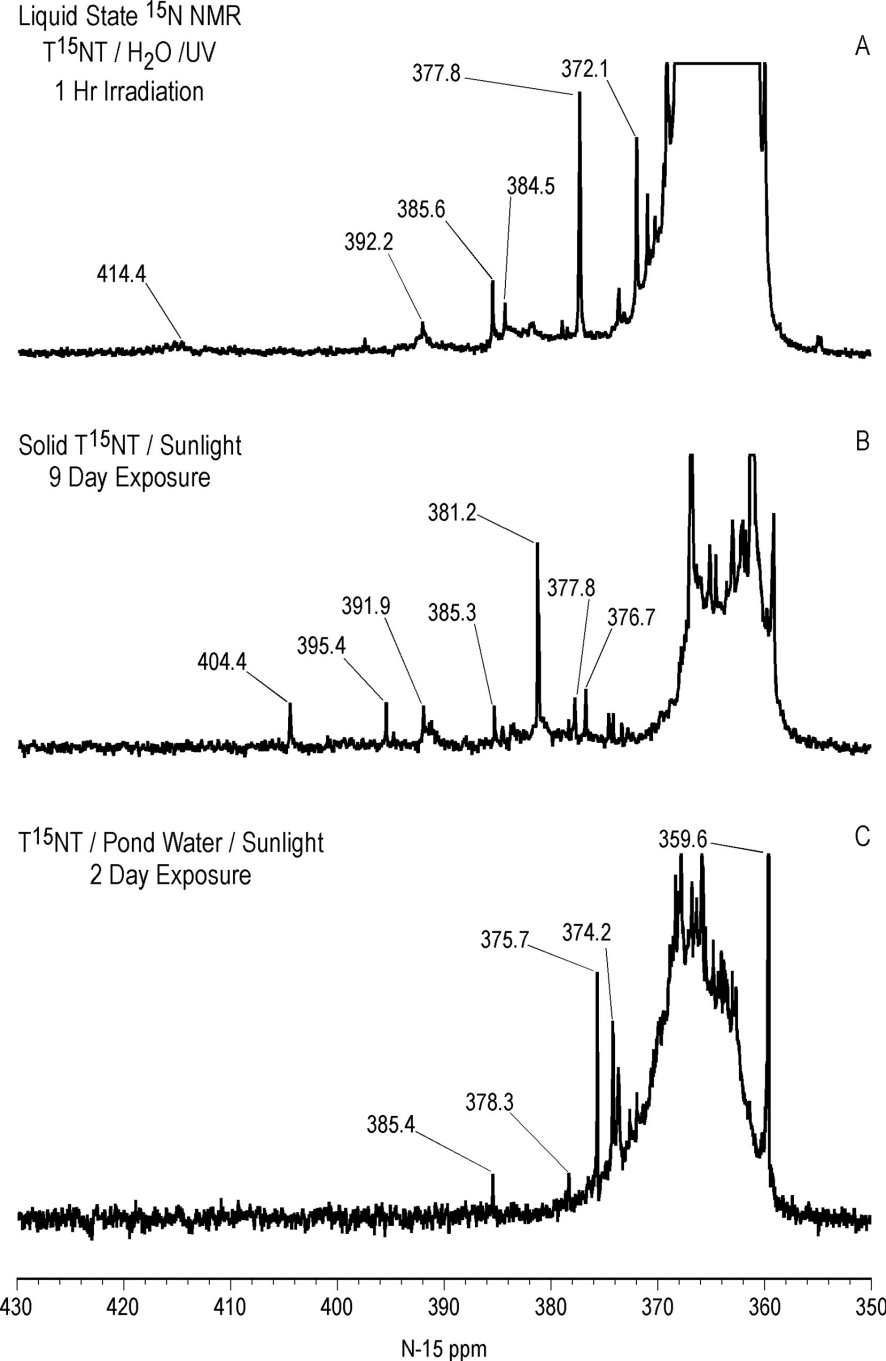
Liquid state N-15 NMR spectra of nitro region of irradiated T^15^NT. Vertical scale expansion. Line broadening = 1.0 Hz. A. 1 hour lamp irradiation in deionized water, IGD spectrum. B. 9 day sunlight irradiation in the solid state, ACOUSTIC spectrum. C. 2 day sunlight irradiation in pond water, IGD spectrum.

Liquid state ^15^N NMR spectra of the 1 hour photolysate in deionized water are shown in Figs 11–16 and S5 Fig. (Liquid state IGD spectra of 1 and 16 hour photolysates from unfiltered irradiation (S6 and S7 Figs) are discussed in S1 Text.) The liquid state ^15^N NMR chemical shifts in dmso-d_6_ for the nitro groups of T^15^NT in the 2,6 and in the 4 positions are 366.7 and 361.1 ppm, respectively (Fig 11A). The liquid state IGD ^15^N spectrum of the one hour photolysate confirms the multicomponent nature of the product mixture and illustrates the photochemical transformation of the nitro groups into a variety of other functionalities, as well as mineralization to ammonium, evidenced by the peak at 23.0 ppm (Fig 11B). Under the acidic conditions of this reaction, nitrite released from the TNT is unstable and either goes on to react as nitrous acid with other transformation products, or undergoes disproportionation to nitric oxide and nitrate. Nitrite is therefore not observed in the spectrum. This is in contrast to the IGD spectrum of the pond water photolysate, where nitrite is observed at 614.8 ppm, a reflection of the slightly alkaline conditions of this irradiation in which nitrite is stable (Fig 11C). The major sets of peaks in the spectrum of the one hour photolysate in deionized water (Fig 11B) correspond to 1° aromatic amine and 1° aminoquinone (50 to 100 ppm), amide and phenylhydroxylamine (100 to 160 ppm), azoxy and imine (290 to 350 ppm), nitro, benzaldoxime, and benzisoxazole (350 to 430 ppm), nitrosophenol (390 to 430 ppm), and azo (490 to 530 ppm) nitrogens (Table 3). The ^15^N NMR chemical shift literature is limited for some of these compound classes, and the assignments must also take into consideration the overlap of chemical shifts among the compound classes (Figs 6–8). Nitrogen-15 chemical shifts for aromatic amine and nitro groups of specific compounds are listed in Tables 4 and 5, respectively. The argument of Burlinson and Kaplan [21, 34, 36, 37] that the insoluble brown residue from TNT photolysis in distilled water consists of azo and azoxy oligomers is supported by the presence of these nitrogens in the spectrum.

**Table 3:** Assignments for N-15 NMR Spectra.

Chem Shift Range, ppm	Assignment
530–490	Azo
480–448	Azo-hydrazone (= N-)
430–390	Nitrosophenol Benzofurazan Azo-hydrazone (= N-)
390–350	Nitro Benzaldoxime Benzisoxazole Azoxy
350–290	Azoxy Aldimine Indazole (= N-) Nitrone Diazonium (N2)
290–230	Nitrile Nitrone Diazonium (N1) Azo-hydrazone (>N-H)
220–180	Imidazole Indazole (>N-H)
180–160	Oxiziridine Indazole (>N-H)
160–120	Benzanilide Phenylhydroxylamine
120–100	1° Amide
100–80	Diphenylamine Hydrazobenzene 1° Aminoquinone
80–50	Aromatic Amine

**Table 4:** N-15 NMR chemical shifts of aromatic amine nitrogens ^a^.

Compound	^15^N Chemical Shift
2-amino–4,6-dinitrobenzoic acid	74.2
2-amino-4-nitrobenzoic acid (4-nitroanthranilic acid)	74.1
4-amino-2,6-dinitrobenzoic acid	73.5
2-amino-4,6-dinitrotoluene	70.6
3,5-dinitroaniline	69.3
4-amino-2,6-dinitrotoluene	66.9
2-methyl-3-nitroaniline	63.5
2-methyl-5-nitroanaline	63.4
2,4-diamino-6-nitrotoluene	62.4, 59.8
2,6-diamino-4-nitrotoluene	62.0
4-methyl-3-nitroaniline	61.1
2,4,6-triaminotoluene	58.3, 55.2
2,6-diaminotoluene	57.7
2,4-diaminotoluene	57.2, 56.3
2-amino-4-nitrophenol	51.8

^a^ Determined in this laboratory in dmso-d_6_.

**Table 5:** Liquid State N-15 NMR chemical shifts of nitro and benzaldoxime nitrogens^a^.

Compound	^15^N Chemical Shift of Nitro or Benzaldoxime
2-methyl-3-nitroaniline	379.8
4-methyl-3-nitroaniline	377.9
2-methyl-5-nitroaniline	372.8
2-amino-4,6-dinitrotoluene	373.8(6) 367.2(4)
4-amino-2,6-dinitrotoluene	373.5
2,6-dinitrotoluene	372.4
2-amino-4-nitrophenol	370.4
2,4-dinitrotoluene	370.3 (2) 365.9 (4)
2-amino-4-nitrobenzoic acid (4-nitroanthranilic acid)	370.0
2-amino-4,6-dinitrobenzoic acid	369.1 (6) 363.2 (4)
4-amino-2,6-dinitrobenzoic acid	367.4
2,4-dinitrobenzoic acid	367.1 (2) 363.7 (4)
2,4,6-trinitrotoluene (acetone)	366.8 (2,6) 360.7 (4)
2,4,6-trinitrotoluene (dmso)	366.7 (2,6) 361.1(4)
2,4-dinitrophenol	366.4 (2) 365.8 (4)
3,5-dinitroaniline	365.8
1,3-dinitrobenzene	364.3
2,4,6-trinitrobenzaldehyde	361.1 (2,6) 359.6 (4)
2,6-dinitrobenzaldehyde	365.6
1,3,5-trinitrobenzene	360.9
2,4,6-trinitrobenzoic acid ^b^	361.9 (2,6) 360.2 (4)
2,6-dinitrobenzaldehyde oxime ^c^	376.2

^a^ Determined in this laboratory in dmso-d_6_ unless otherwise noted.

^b^ In dmso-d_6_, Ref (73).

^c^ Oxime nitrogen from reaction of ^15^N-labelled hydroxylamine hydrochloride with 2,6- dinitrobenzaldehyde.

The horizontal expansion of the ACOUSTIC ^15^N spectrum from 60 to 90 ppm (Fig 14A) shows a number of discreet peaks, including those at 70.8, 72.1, 72.9, 75.2, 75.6, 76.5, 77.3, 81.6, 83.3, 86.3, and 87.7 ppm. The chemical shift range for primary aromatic amines is approximately 50 to 77 ppm. For standards of primary aromatic amine derivatives of TNT and dinitrotoluenes that were commercially available, the most downfield ^15^N chemical shift measured was 74.2 ppm, for 2-amino-4,6-dinitrobenzoic acid (Table 4). The peaks with chemical shifts from 80–90 ppm then cannot definitively be assigned as primary aromatic amines, from the limited chemical shift data available, and as nitrogens shown to be bonded to two protons in the next section, may possibly correspond to 1° aminoquinone structures.

In comparing the IGD and DEPT spectra of the one hour photolysate (Fig 12), it becomes apparent from the DEPT spectrum that there is continuum of resonances from approximately 70 to 160 ppm. The DEPT spectrum of Fig 13A shows all nitrogens directly bonded to protons in positive phase. These are further resolved in the DEPT spectra of Fig 13B, which indicates the peaks from 70 to 120 ppm are primarily nitrogens bonded to two protons (1° aromatic amines, 1° aminoquinones, and 1° amides) and the peaks from 120 to 160 ppm nitrogens bonded to one proton (2° amides and phenylhydroxylamines). Some of the aromatic amine peaks (e.g. 70.8 and 76.5 ppm) present in the IGD spectrum are attenuated or not visible in the DEPT spectra (Fig 12), indicating that these nitrogens are exchanging too rapidly with protons for polarization transfer to occur.

Peaks from approximately 90 to 120 ppm in the DEPT spectra are assumed to be 1° amides, although some 1° aminoquinone nitrogens can occur in this region (Figs 7 and 8). The DEPT spectra exhibit discrete resonances at 95.7, 103.7, 107.4, 110.9, and 113.7 ppm. Primary amides could arise from both photochemical and non-photochemical Beckmann rearrangements of aldoximes, whereas 1° aminoquinones could arise from condensation of ammonia with quinones, although the former reaction is favored at alkaline rather than acidic pH. (As discussed further on, the DEPT ^15^N NMR spectrum of the photolysate from sunlight irradiation of TNT in the solid state, where greater S/N was obtained, showed a few nitrogen peaks singly bonded to protons around 84–87 ppm.)

The chemical shifts of phenylhydroxylamines and 2° amides overlap in the region from 130 to 140 ppm (Figs 7 and 8), which defines the peak maximum for the nitrogens singly bonded to protons (Fig 13). Although not previously reported as photodegradation products, phenylhydroxylamines have been detected as intermediates in the anaerobic microbial reduction of TNT and dinitrotoluenes [78, 79], and are presumed to be intermediates in the photochemical transformation of TNT into aromatic amine, azoxy, and 2° amide compounds. As discussed in the introduction, a plausible pathway to 2° amides is the photochemical rearrangement of nitrones formed from condensation of phenylhydroxylamines with benzaldehydes (Fig 3). Although phenylhydroxylamines and 2° amides cannot be resolved from one another in the ^15^N NMR, based on the susceptibility of phenylhydroxylamines to undergo further reactions or transformations, it would be reasonable to assign the major peaks at ~ 130 ppm as predominantly 2° amides, with some possible contribution from phenylhydroxylamines. For convenience, this peak will be referred to as the 2° amide/PHA nitrogens.

Several unidentified discrete peaks occur in the region from approximately 160 to 290 ppm of the IGD spectrum, including those at 180.2, 187.4, 205.5, 234.3, and 280.0 ppm (Fig 12B and S5 Fig). Possible assignments are the >N-H nitrogens of indazoles (180.2 & 187.4 ppm), imidazole, pyrazole, amidine, or imidate nitrogens (205.5 ppm), N1 nitrogens of diazonium ions or >N- nitrogens of azo-hydrazones (234.3 ppm), and nitrone nitrogens (280.0 ppm).

The low intensity peak at 310.0 ppm corresponds to N_2_ gas (Fig 12B), which most likely arises from aryl diazonium ions that were stable in the freeze dried powder but underwent subsequent decomposition after re-dissolution in dmso-d_6_ for the NMR acquisition. The N_2_ peak then constitutes supporting evidence for the occurrence of nitrosation reactions under the conditions of this irradiation. Nitrogen gas peaks were also observed in liquid state ^15^N NMR spectra of NOM nitrosated with Na^15^NO_2_, and NOM irradiated with unfiltered UV light in the presence of Na^15^NO_3_ [59, 80]. Nitrogen gas can also form from the decomposition of hydroxylamine, but the decomposition is not favored at the pH of this irradiation [81].

The nitrogens from approximately 320 to 340 ppm (Fig 12B), with several discreet peaks, including those at 322.7, 324.9, 326.7, and 330.0 ppm (S5 Fig), are assigned primarily to azoxy groups. Standards for TNT-derived azoxy compounds in quantities sufficient for NMR analysis are not commercially available, however liquid state chemical shifts of the major azoxy products formed during Caro acid oxidation of 2-amino-^15^N-4,6-dinitrotoluene and 4-amino ^15^N-2,6-dinitrotoluene were detected in the range from 320 to 342 ppm. The Schiff base derivatives from condensation of aromatic amines with benzaldehydes (aldimines) may also contribute to the region from 320 to 340 ppm (Fig 7). Nitrogens in the # 2 position of indazole structures (= N-) occur in this region as well (Fig 7).

Azoxybenzenes are known to undergo photochemical rearrangements to hydroxy-azo compounds (photo-Wallach rearrangement), in cases where there is a free ortho position in the ring constituent more distant from the N-O function (Fig 4). In this rearrangement, the oxygen atom of the azoxy group migrates to the ortho position of the aryl group more distant from the N-O function [63, 82]. It is conceivable that azoxybenzenes arising from 4-nitroso or 4-phenylhydroxylamino derivatives of TNT could be susceptible to this rearrangement. Hydroxyazo compounds in turn undergo an azo-hydrazone tautomeric equilibrium (Fig 6). The ^15^N NMR chemical shifts correspond to the equilibium position and range from ~303 to 179 ppm for the >N- nitrogen and ~448 to 373 ppm for the = N- nitrogen, respectively (Fig 8). An example is Sudan I dye (1-phenylazo-2-naphthol), which has chemical shifts of 236 ppm and 394 ppm for these nitrogens in the solid state (Fig 6).

The major peak from 350 to 380 ppm corresponds mainly to the residual nitro groups in the photochemical transformation products (Fig 12B), but benzisoxazole, benzaldoxime, and azoxy nitrogens also occur in this region (Figs 6 and 8). A number of discrete peaks are visible in the horizontal expansion of the nitro region (Figs 15A and 16A), including the peak at 377.8 ppm assumed to be nitrate ion. The peaks at 392.2 and 414.8 ppm on the downfield shoulder of the residual nitro peak are in the chemical shift range characteristic of nitrosophenol nitrogens (tautomeric equilibrium between quinone monoxime and nitrosophenol nitrogens; Fig 2). The nitrosophenol nitrogens may correspond to intermediate nitroso reduction products with hydroxyl groups in substitution patterns that would conform to this equilibrium, or from nitrosation of phenolic degradation products of the TNT. Alternative assignments for these peaks would be benzofurazan nitrogens or the -N = nitrogens involved in an azo-hydrazone tautomerism (Fig 6).

Azo nitrogens occur from approximately 500 to 540 ppm in Fig 12B. A discreet peak at 511.1 ppm among the broad band of azo resonances indicates a major degradation product, possibly the 2,2'-dicarboxylic-2,3',5,5'-tetranitro-azobenzene identified by Burlinson [34]. The azo compounds presumably arise primarily from condensation of aromatic amines and nitroso groups, but as discussed previously, alternative pathways such as the photochemical reduction or rearrangement of azoxy compounds, and diazonium coupling with phenols, may be considererd.

Identification of specific photodegradation products based upon the aromatic amine and nitro group chemical shifts in the ^15^N NMR spectra is limited by the chemical shift data available (Tables 4 and 5). 2,4,6-Trinitrobenzoic acid can be tentatively identified from the discrete resonances at 360.0 and 362.1 ppm in the nitro region (Fig 15A).

Integration of the IGD ^15^N NMR spectrum indicates that nitrogens are approximately distributed as 5% Azo, 71% Nitro/Benzaldoxime/Benzisoxazole, 8% Azoxy, 10% Amide, and 6% Aromatic Amine in the photolysate.

#### Solid state ^15^N NMR

**Fig 17:**
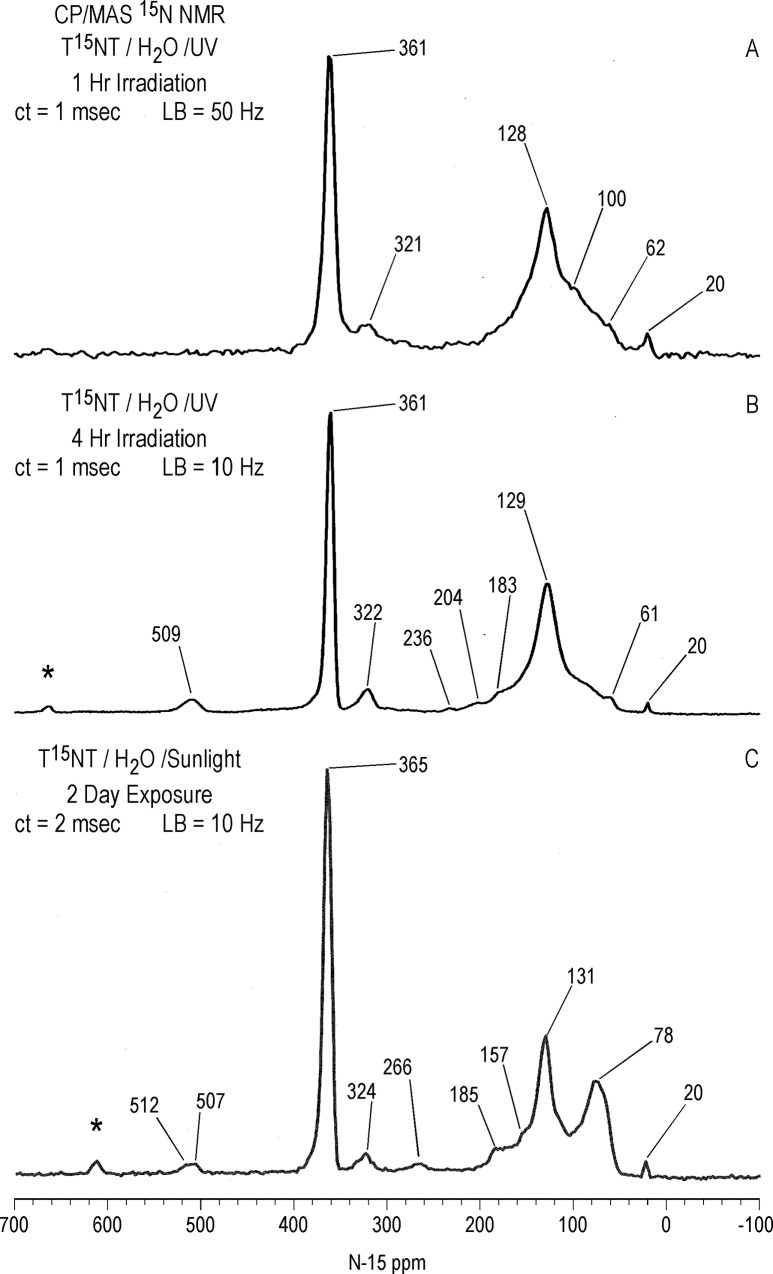
Solid state CP/MAS N-15 NMR spectra of T^15^NT. A 1 hour lamp irradiation in deionized water. B. 4 hour lamp irradiation in deionized water. C. 2 day sunlight irradiation in deionized water. ct = contact time. LB = line broadening. SS = spinning speed. Asterisk = spinning side band.

As in the case with ^13^C, there is a significant difference in resolution between the liquid and solid state ^15^N NMR spectra of the 1 hour photolysate in deionized water (Fig 17A). The 2,4- and 6-nitrogens of the T^15^NT standard are not resolved in the solid state but appear as a single peak at 366 ppm. The solid state CP/MAS ^15^N NMR spectrum of the 1 hour photolysate exhibits a broad band from about 50 to 220 ppm, with the maximum at 128 ppm corresponding to the 2° amide/PHA nitrogens (Fig 17A). Peaks corresponding to ammonium (20 ppm), azoxy (321ppm), and nitro (361ppm) nitrogens are also visible in spectrum. Of note, the azo nitrogens present in the liquid state spectrum from 500 to 540 ppm (Fig 11B) are not observed in the solid state spectrum of the 1 hour photolysate. This may result from a relative insensitivity of azo nitrogens to the CP experiment, but confirmation by more detailed analyses of spin dynamics is required. Azo nitrogens are observed in the ^15^N NMR spectrum of the 4 hour photolysate (Fig 17B), suggesting an increase in azo group formation from 1 to 4 hours. With the greater S/N ratio attained for this spectrum, very low intensity peaks at 183 (indazole), 204 (imidazole, pyrazole, amidine, or imidate), and 236 ppm (diazonium or azo-hydrazone) become visible.

Judging from the ratio of nitro nitrogens to other nitrogens, the degree of photochemical transformation of the nitro groups of T^15^NT in deionized water from 2 days of sunlight exposure is of a comparable order of magnitude to the lamp irradiations (Fig 17C). Here again there is a broad peak from about 50 to 200 ppm, however within this region aromatic amine nitrogens (78 ppm) are more prominent and are well resolved from the 2° amide/PHA nitrogens. Ammonium (20 ppm), indazole (185 ppm), azoxy (324 ppm), and azo (507 & 512 ppm) peaks are also present. Of note is the peak at 266 ppm, which correlates with benzonitrile nitrogens (Fig 7).

#### Transformation versus mineralization

The mass yield from a replicate of the TNT solution (4.90 X 10^-4^M) subjected to the one hour Pyrex-filtered lamp irradiation in deionized water was 93%, including bicarbonate, nitrate, and ammonium, which were not separated or quantified from the organic transformation products. As the NMR spectra indicated that the concentrations of these inorganic constituents were minor, the bulk of the TNT has undergone transformation and not mineralization. Under these experimental conditions then loss of volatiles in the form of N_2_, NH_3_, CO_2_, or nitrogen oxides did not greatly exceed uptake of oxygen.

### Irradiation of 4-amino-^15^N-2,6-dinitrotoluene (4ADNT) and 2-amino-^15^N-4,6-dinitrotoluene (2ADNT) in deionized water

**Fig 18:**
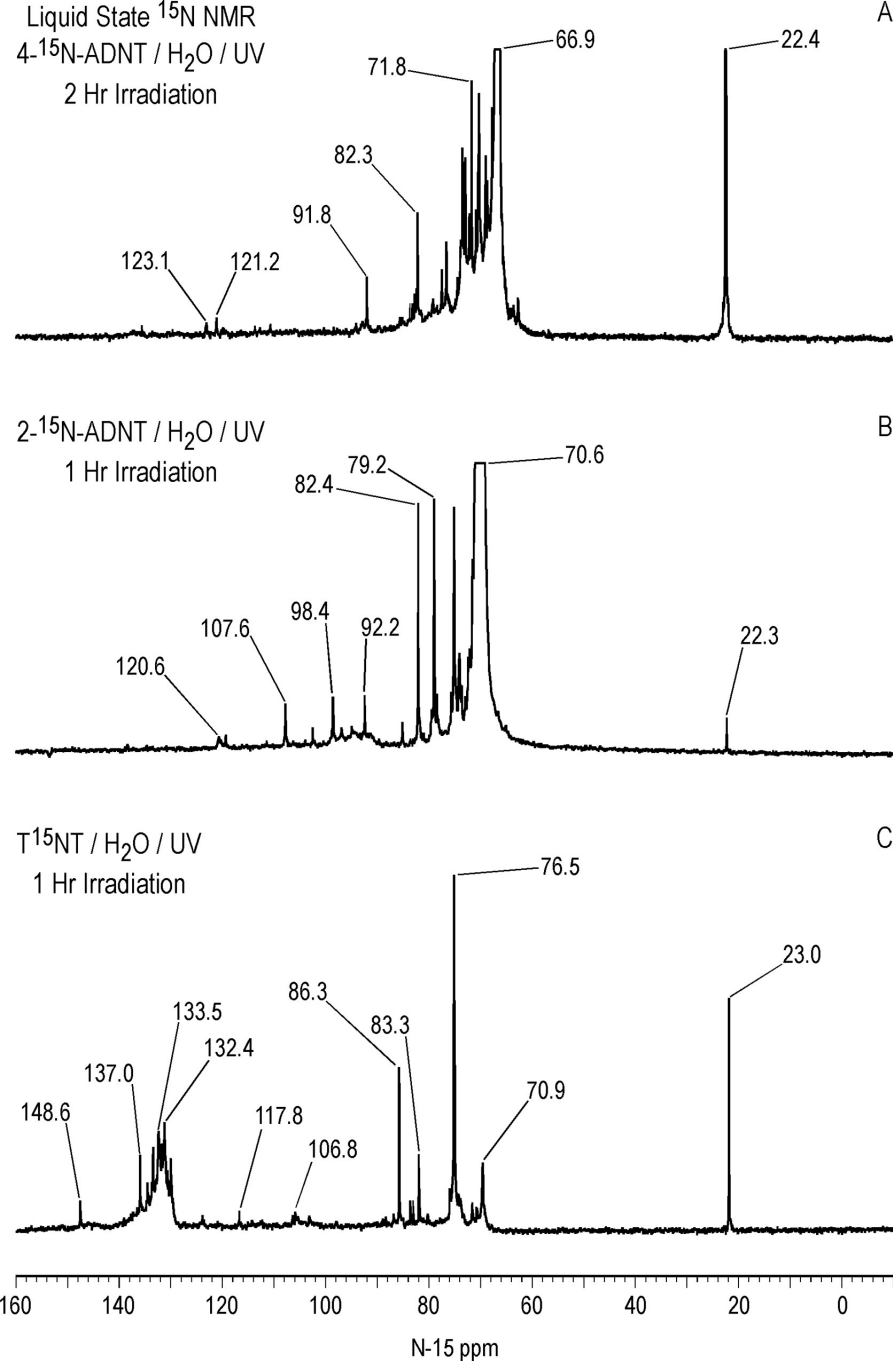
Liquid state N-15 NMR spectra of 4-^15^N-amino-2,6-dinitrotoluene, 2-^15^N-amino-4,6-dinitrotoluene, and T^15^NT subjected to lamp irradiation in deionized water. Line broadening = 1.0Hz. Spectra phased 180°. A. Continuous decoupled spectrum. B. Continuous decoupled spectrum. C. ACOUSTIC spectrum.

**Fig 19:**
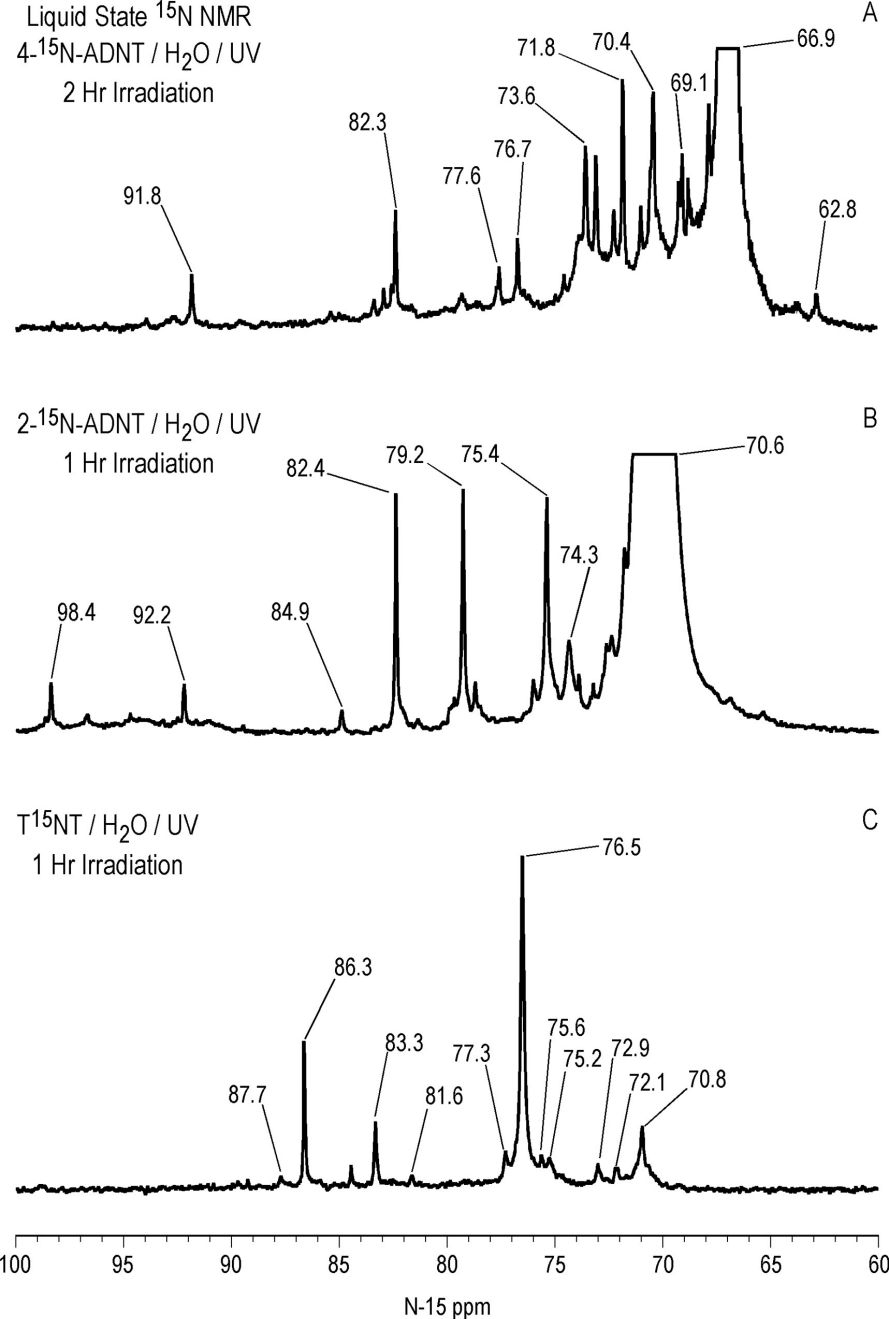
Liquid state N-15 NMR spectra of 4-^15^N-amino-2,6-dinitrotoluene, 2-^15^N-amino-4,6-dinitrotoluene, and T^15^NT subjected to lamp irradiation in deionized water. Horizontal and vertical scale expansion. Line broadening = 1.0 Hz. Spectra phased 180°. A. Continuous decoupled spectrum. B. Continuous decoupled spectrum. C. ACOUSTIC spectrum.

Liquid state ^15^N NMR spectra (continuous decoupled) of 4ADNT and 2ADNT, both labeled with ^15^N in the amine position only, subjected to 1 and 2 hour irradiations in deionized water respectively, are compared to the 1 hour T^15^NT photolysate (ACOUSTIC spectrum) in Figs 18 and 19. The spectra provide insight into the fate of the amine groups upon photolysis and the reaction pathway to the nitrogens assigned as 2° amide/PHA’s.

The region of the 4ADNT and 2ADNT photolysate spectra where peaks are visible without vertical expansion is approximately 20 to 130 ppm. The major peaks in the 4ADNT and 2ADNT spectra correspond to the parent amine nitrogens at 66.9 and 70.6 ppm, respectively.

There are a number of additional aromatic amine peaks, including those at 69.1, 70.4, 71.8, 73.6, 76.7, and 77.6 ppm in the 4ADNT photolysate, and those at 74.3, 75.4, and 79.2 ppm in the 2ADNT photolysate (Fig 19). Peaks from about 80 to 120 ppm may include 1° amide and 1° aminoquinone nitrogens.

The spectra suggest that 4ADNT and 2ADNT are photochemically transformed to new aromatic amine and other nitrogen compounds through a combination of condensation reactions and changes in the substitution patterns of the aromatic rings as outlined for the TNT. The presence of the ammonium peak at 22.4 ppm in the 4ADNT photolysate indicates that ammonia can arise directly via deamination of the parent 4ADNT or transformation products. This is an alternative pathway to the photochemical release of nitrite from the nitroaromatic compounds followed by reduction of the nitrite to ammonia via hydroxylamine. Schmelling and Gray had previously concluded that deamination of aromatic amines is a pathway to ammonium during photodegradation of TNT [52]. The 2ADNT photolysate also exhibits an ammonium peak at 22.3 ppm, but at lower concentration.

Comparing the three spectra, the most notable feature of the 4ADNT and 2ADNT photolysates is the absence of the peak from 130 to 140 ppm assigned as 2° amide/PHA nitrogens in the T^15^NT photolysate (Fig 18). This supports, but does not prove, the assignments and proposed formation pathways for these nitrogens. In other words, the ^15^N label is already in the form of the reduced aromatic amine in 4ADNT and 2ADNT. There are no ^15^N-labeled nitro or nitroso groups available to undergo reduction to phenylhydroxylamines, and therefore no ^15^N-labeled phenylhydroxylamines available for condensation with benzaldehydes to form nitrones that rearrange to benzanilides.

Vertical expansion of the downfield region of the 2ADNT spectrum revealed the naturally abundant nitro peaks of the unreacted standard at 367.2 and 373.8 ppm (S8 Fig), indicating the possibility that some of the very low intensity peaks in the spectra of the 2ADNT and 4ADNT photolysates could originate from the unlabeled nitro peaks. This may be the case with the peaks at 121.2 and 123.1 ppm in the 4ADNT and 120.6 ppm in the 2ADNT photolysates (Fig 18) for example, which possibly represent 1° amides. Additionally, upon vertical expansion, the 4ADNT photolysate spectrum contained low intensity peaks from 294 to 327 ppm, in the approximate range of azoxy, imine, nitrone, and indazole (N2) nitrogens, and 208 to 236 ppm, in the range of diazonium and azo-hydrazone nitrogens (S8 Fig). Vertical expansion of the 2ADNT photolysate spectrum also showed a low intensity, broad peak from 285 to 315 ppm (S8 Fig), which could include the 4,6-dinitroindazole reported by Burlinson and Glover [37].

### Sunlight irradiation of TNT in the solid state

#### ^13^C NMR

**Fig 20:**
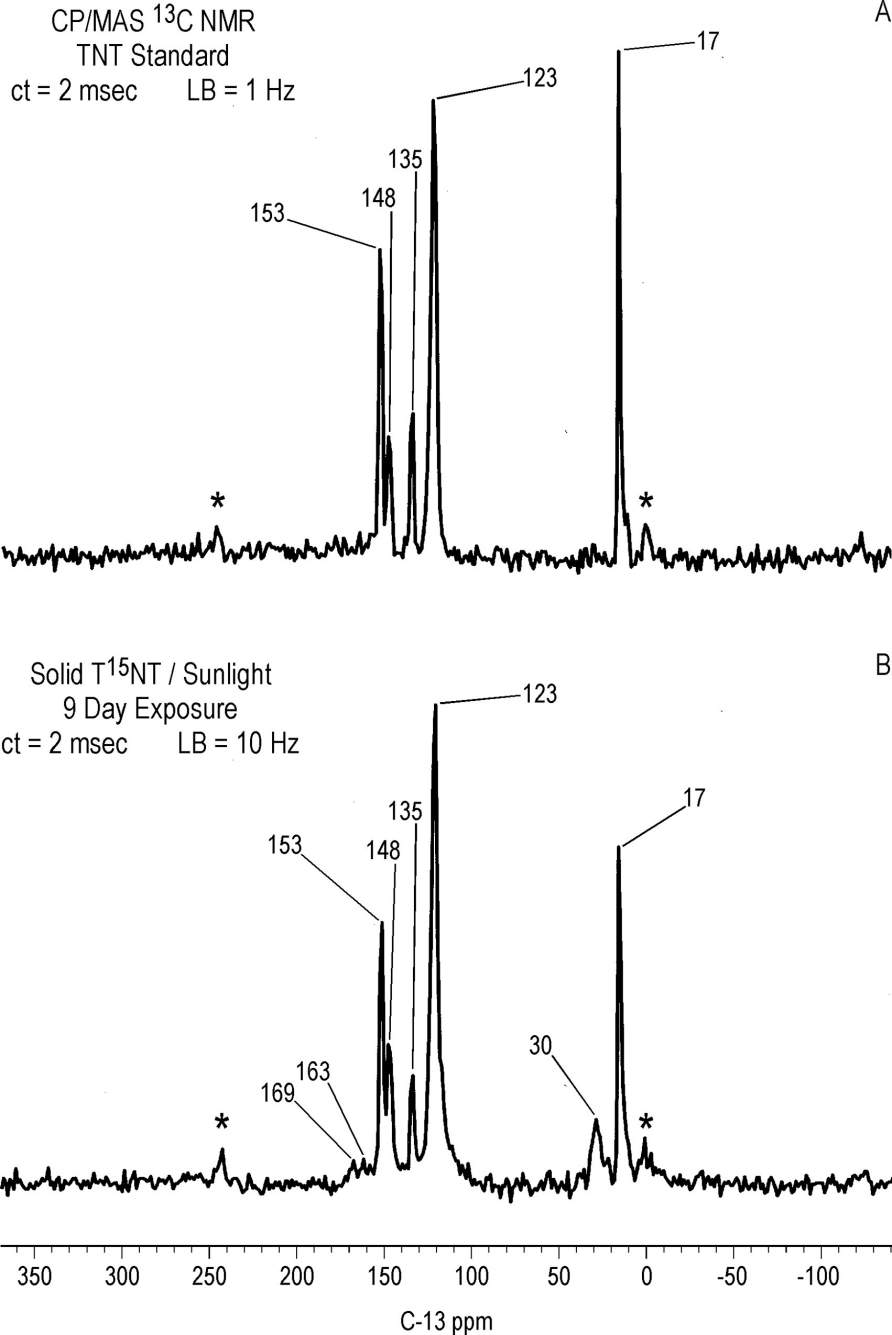
Solid state CP/MAS C-13 NMR spectra of T^15^NT. A. Standard. B. Solid T^15^NT subjected to sunlight irradiation. Spinning speed = 6 KHz. ct = contact time. LB = line broadening. Asterisk = spinning side band.

In general the NMR spectra of the T^15^NT exposed to sunlight in the solid state show similarities to the spectra of the photolysates from deionized water in terms of functional groups identified. The liquid state ^13^C NMR spectrum (Fig 9D) shows prominent peaks at 187.8, 147.7, 134.2 and 124.6 ppm, denoted by the triangles, corresponding to 2,4,6-trinitrobenzaldehyde, which is a significant transformation product in the photolysate. Carboxylic acid/amide peaks are present from 159 to 163 ppm, as well as the peaks from about 100 to 118 ppm also observed in the photolysate from irradiation in deionized water (Fig 9B). As with the latter case, there is no evidence for the alcohol carbon peak of 2,4,6-trinitrobenzylic alcohol. Unidentified peaks occur at 66.2 and 99.6 ppm. There is a minor methylene carbon peak at 32.3 ppm, while the methyl carbon to the parent TNT is not visible. The solid state spectrum (Fig 20B) in contrast does show the methyl carbon to the parent TNT at 17 ppm. The only direct evidence for transformation products in the solid state spectrum is the methylene carbon peak at 30 ppm, and the very minor carboxyl/amide peaks at ~160 to 170 ppm, signals barely above the noise. The aldehyde carbon of 2,4,6-trinitrobenzaldehyde again is not detected in the solid state experiment.

#### ^15^N NMR

**Fig 21:**
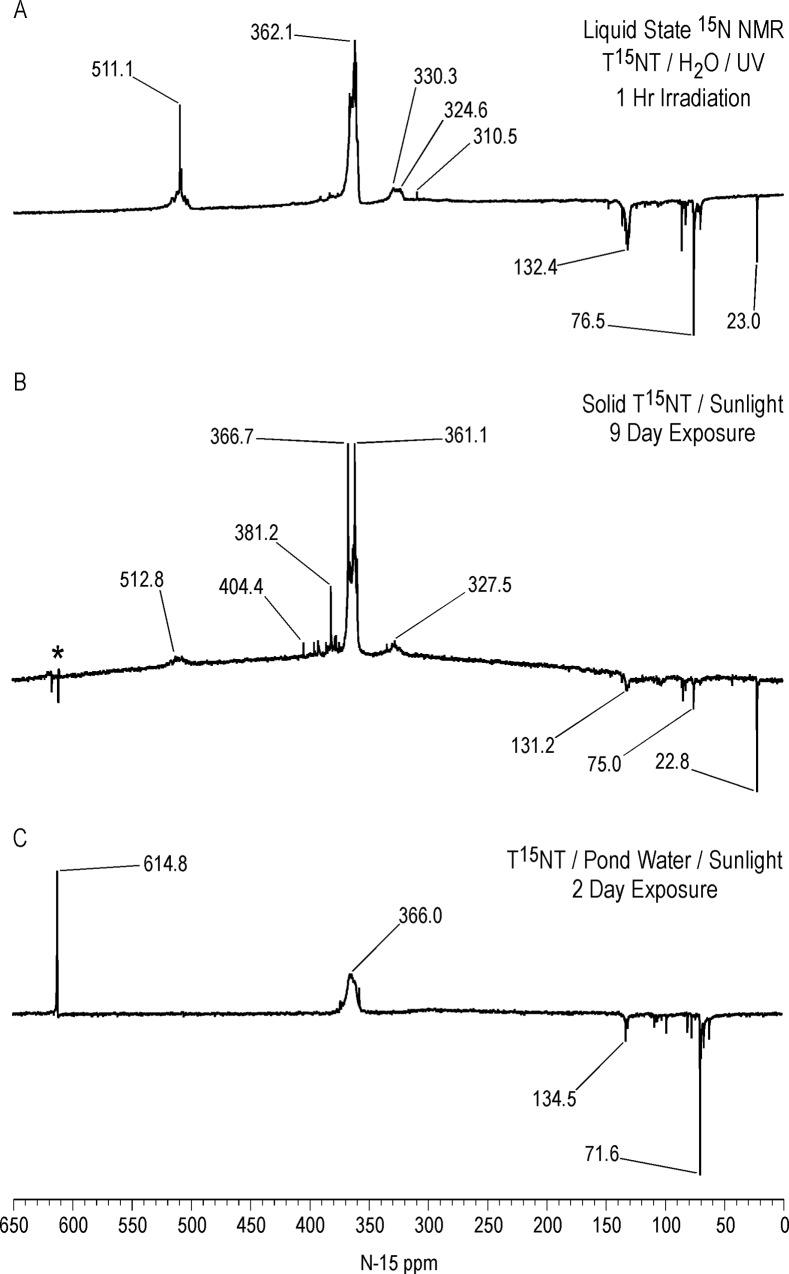
Liquid state ACOUSTIC N-15 NMR spectra of T^15^NT. A. Lamp irradiation in deionized water. B. Sunlight irradiation in the solid state. C. Sunlight irradiation in pond water. Line broadening = 3 Hz.

**Fig 22:**
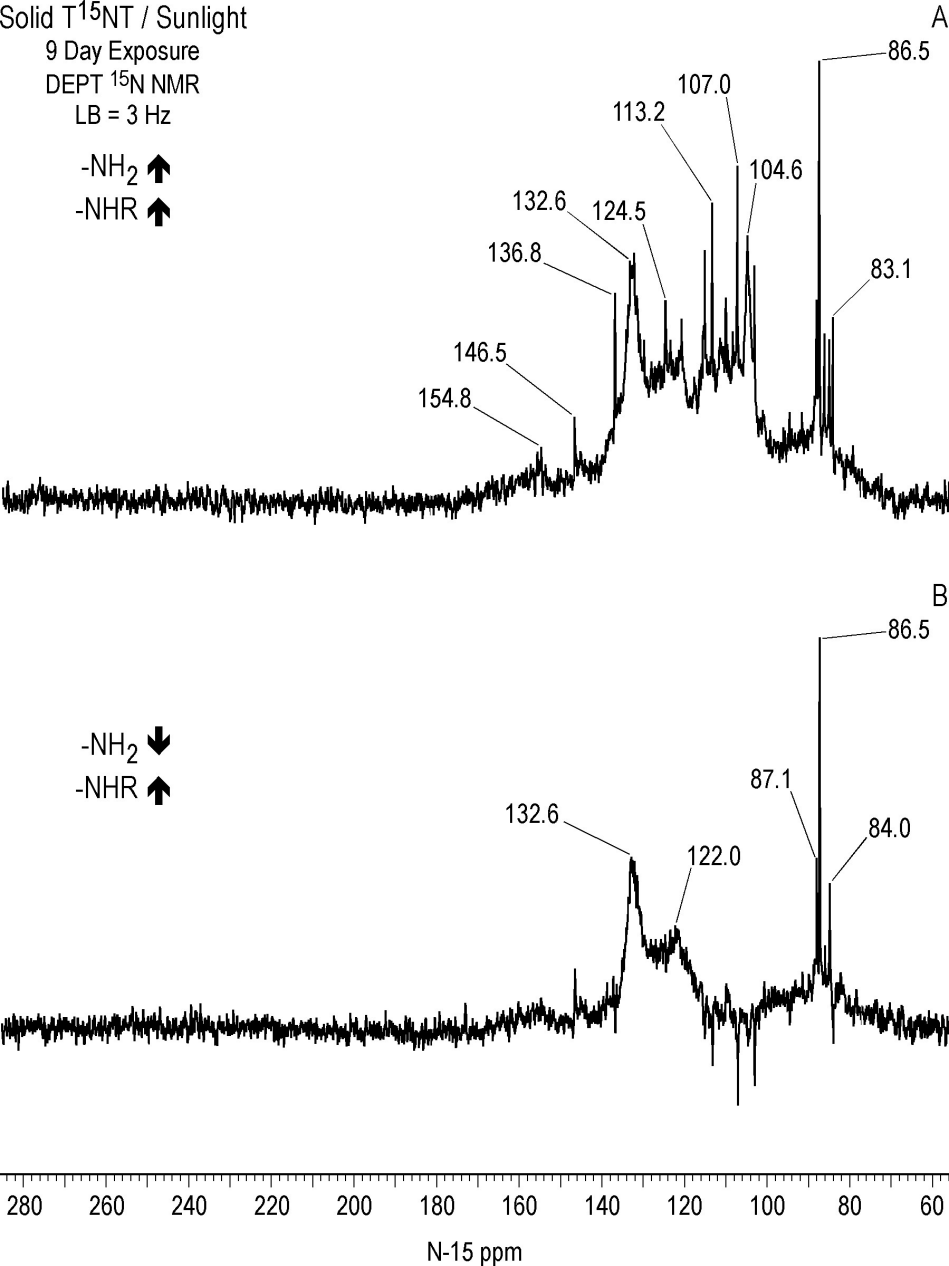
Liquid state N-15 NMR DEPT spectra of solid T^15^NT subjected to sunlight irradiation. A. All nitrogens bonded to protons in positive phase. B. Nitrogens bonded to two protons in negative phase and nitrogens bonded to one nitrogen in positive phase. LB = line broadening.

**Fig 23:**
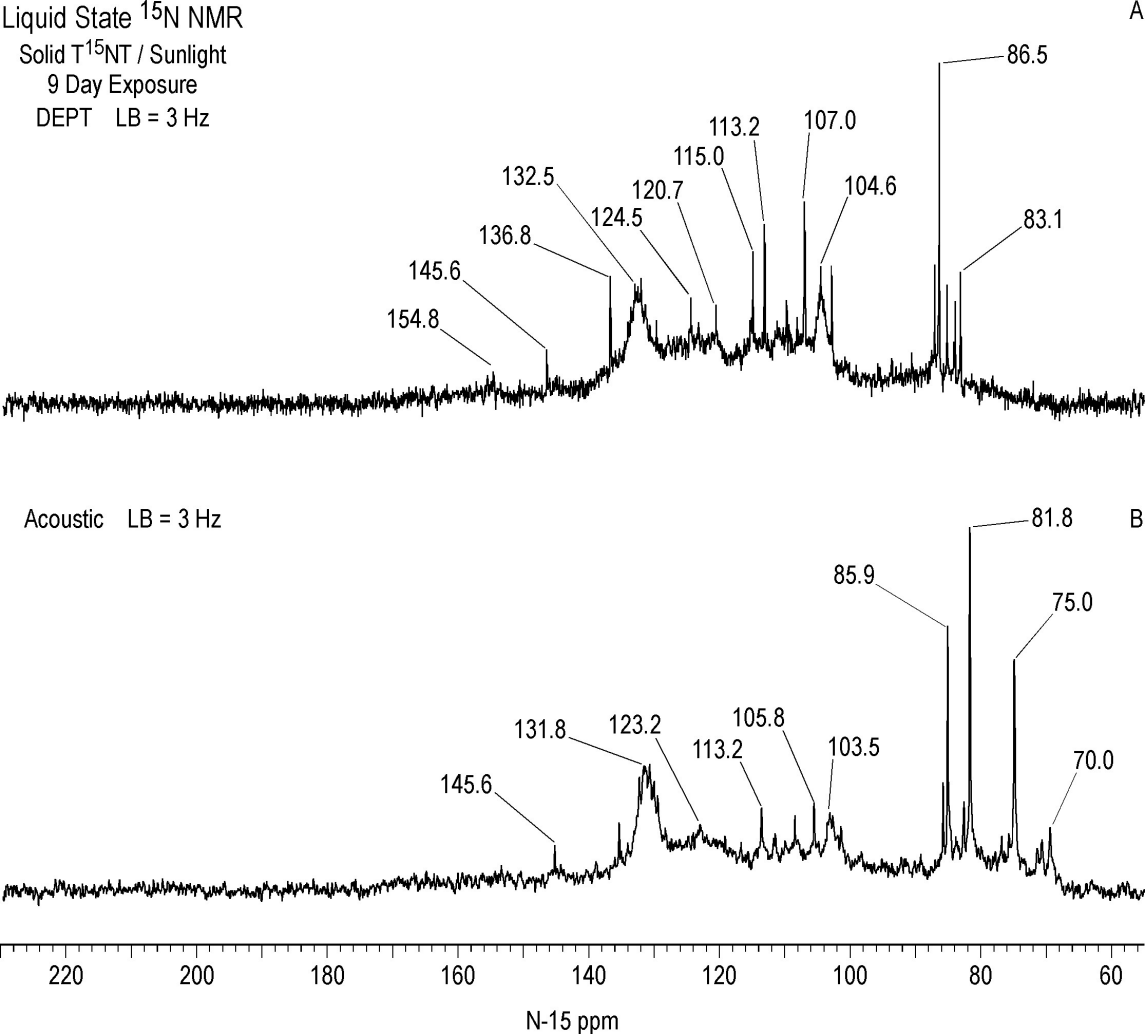
Liquid state N-15 NMR spectra of solid T^15^NT subjected to sunlight irradiation. A. DEPT spectrum showing all nitrogens bonded to protons. B ACOUSTIC spectrum phased 180°. LB = line broadening.

For liquid state ^15^N NMR, an IGD spectrum was not obtained, and so an ACOUSTIC spectrum is shown in Fig 21B along with the comparable spectra of the photolysates from irradiation in deionized water and pond water (Fig 21). Inversion of peaks is due to negative NOE. The photolysate of T^15^NT as a solid shows peaks corresponding to ammonium (22.8 ppm), aromatic amine (75.0 ppm), 2° amide/PHA (131.2 ppm), azoxy (315 to 340 ppm), residual nitro (350 to 390 ppm), nitrosophenol (390 to 410 ppm), and azo nitrogens (495 to 520 ppm). These are the same functional groups present in the photolysate from the one hour lamp irradiation in deionized water. The nitro peaks to the parent T^15^NT are present at 361.1 and 366.7 ppm. The DEPT spectrum showing all nitrogens bonded to protons in positive phase again reveals a continuum of resonances from about 70 to 160 ppm (Fig 22A). Individual peaks occur at 83.1, 84.0, 86.5, 87.1, 104.6, 107.0, 113.2, and 115.0 ppm. Further analysis again shows that in general nitrogens from about 60 to 120 ppm are bonded to two protons while nitrogens from 120 to 170 ppm are bonded to one proton (Fig 22B). The significant exception is the cluster of peaks at 84.0, 86.5, and 87.1 ppm, which are revealed to be nitrogens bonded to one proton, and are in the chemical shift range of diphenylamine and hydrazobenzene (hydrazine) nitrogens (Figs 7 and 8). Diphenylamines have not been previously reported as photodegradation products of TNT, but have been detected as microbial degradation products that result from condensation of phenylhydroxylamines with the Meisenheimer dihydride complex of TNT [83]. Diphenylamines are also known to form from coupling between aniline and nitrobenzene compounds and between aniline and azobenzene compounds [84–86]. Hydrazobenzenes could result from reduction of azobenzenes or free radical coupling of aromatic amines. Finally, the aromatic amine peaks at 70.0 and 75.0 ppm in the ACOUSTIC spectrum are not observed in the DEPT spectra (Fig 23), reproducing the observation made in the comparable spectra of the TNT photolysate from 1 hour of irradiation in deionized water, that some aromatic amine protons exchange too rapidly for detection by polarization transfer.

Comparison of the expanded aromatic amine and nitro regions of the solid and aqueous phase photolysates does indicate differences in the individual degradation products as a function of the reaction conditions employed (Figs 14–16). In the solid state photolysate, 2-amino-4,6-dinitrotoluene can be tentatively identified from the aromatic amine and nitro peaks at 70.8, 367.2, and 374.0 ppm (Figs 14B and 15B).

**Fig 24:**
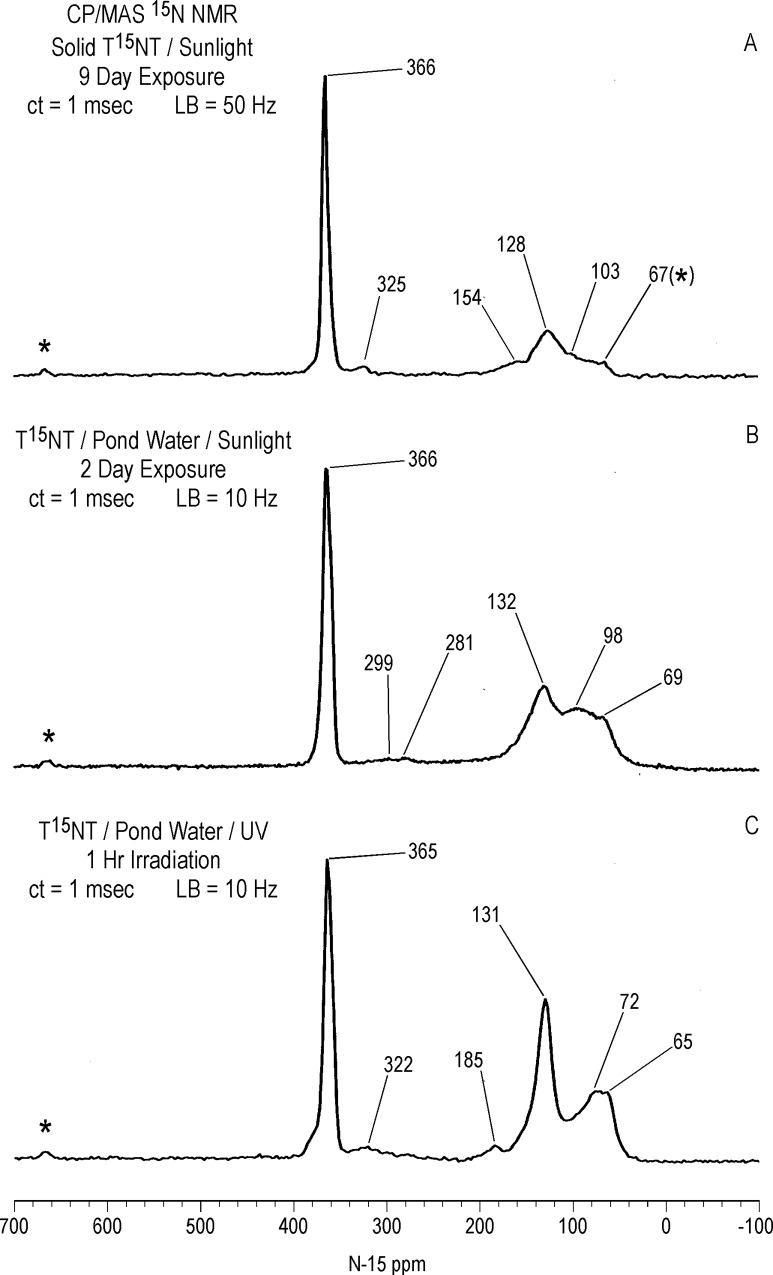
Solid state CP/MAS N-15 NMR spectra of T^15^NT. A. Sunlight irradiation in the solid state. B. Sunlight irradiation in pond water. C. Lamp irradiation in pond water. ct = contact time. LB = line broadening. Spinning speed = 6 KHz. Asterisk = spinning side band.

The solid state CP/MAS ^15^N NMR spectrum of the solid phase photolysate shows a broad band from approximately 50 to 220 ppm, with peak maximum at 128 ppm in the 2° amide/PHA region, an azoxy peak at 325 ppm, and the residual nitro peak at 366 ppm (Fig 24A). The azo and ammonium nitrogens visible in the liquid state ACOUSTIC spectrum are not observed in the solid state spectrum. The ratio of nitro nitrogens to other nitrogens in the solid state spectrum is higher compared to the corresponding spectra of the photolysates from irradiation in deionized water, indicating a lesser degree of transformation.

Although light irradiance was not quantified or normalized in these experiments, in general, the lower amount of degradation of TNT in the solid compared to the aqueous phase, suggested by both the CP/MAS ^13^C and ^15^N spectra (Figs 20B and 24A), and the concentration of parent T^15^NT in the ACOUSTIC spectrum (Fig 15B), is consistent with the observation that photodegradation occurs at the surface of TNT particles [49]. It is interesting to note that molecular formulas attributable to 2° amide or PHA structures were not identified in the mass spectrometric analyses of Kunz et al [49]. On the other hand, 2,4,6-trinitrobenzyl alcohol was detected by DART-TOF/MS but not the NMR analyses here, illustrating the complementarity of both spectroscopic techniques.

### Sunlight and lamp irradiation of T^15^NT in pond water

#### ^13^C NMR

T^15^NT was exposed to both sunlight and lamp irradiation in the pond water (DOC = 3 mg C/L; pH = 8.3). A liquid state ^13^C NMR spectrum was recorded on the sunlight photolysate and shows the aldehyde carbon peak to 2,4,6-trinitrobenzaldehyde at 188.2 ppm, a cluster of peaks centered at 148.2 ppm derived from the C_2,6_ and C_4_ carbons, several peaks from 95.5 to 116.5 ppm, and methylene carbon at 28.9 ppm (Fig 9C). At the line broadening of 1.0 Hz applied to the FID, broad peaks due to the naturally occurring NOM from the pond water are subsumed in the baseline of the spectrum. This accounts for the broadening of the carboxyl/amide peak at 169.2 ppm compared to the other spectra in Fig 9. Solid state ^13^C NMR spectra are shown in S9 Fig (see S2 Text).

#### ^15^N NMR

**Fig 25:**
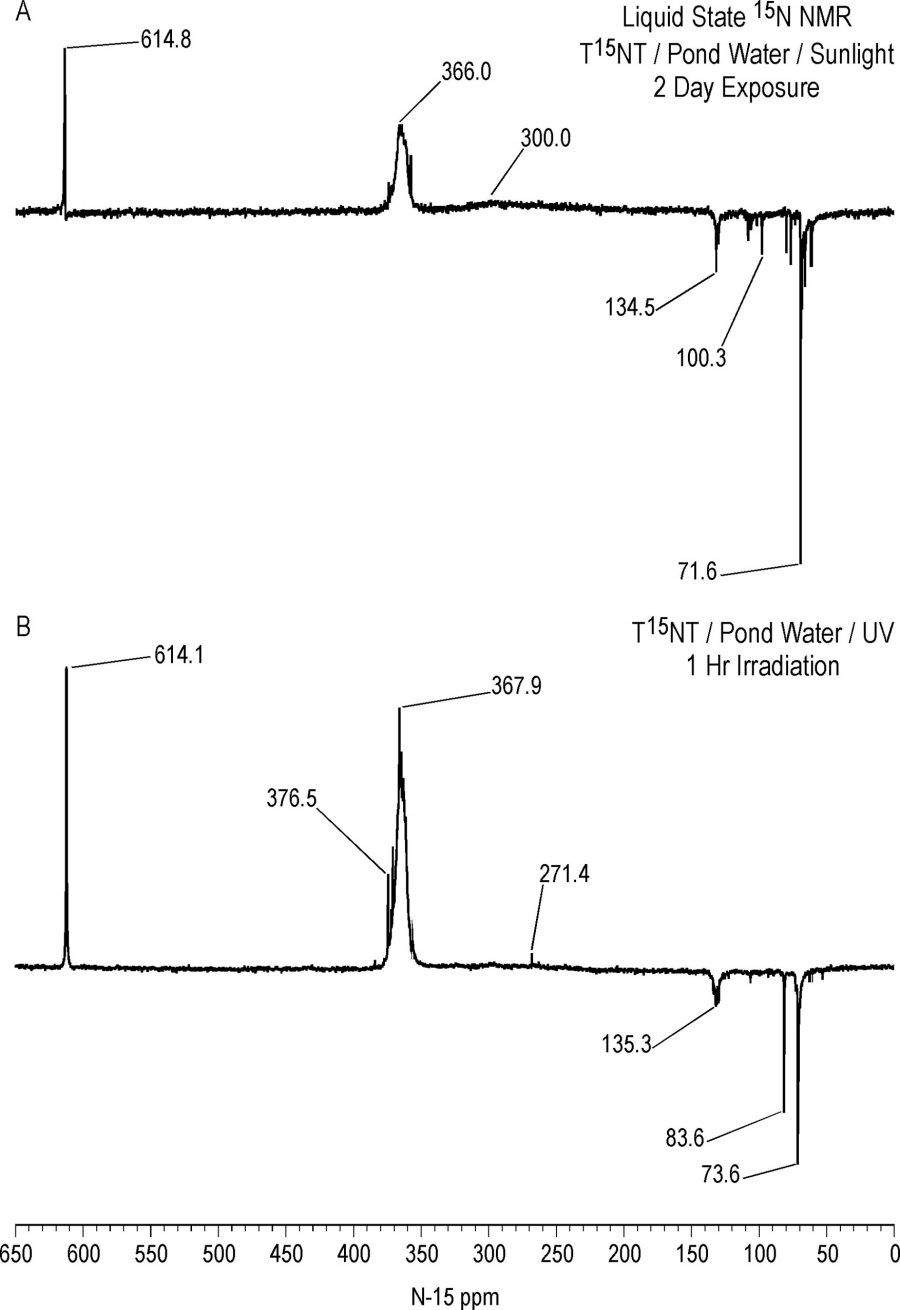
Liquid state N-15 NMR spectra of T^15^NT. LB = 3.0 Hz. A. Sunlight irradiation in pond water, ACOUSTIC spectrum. B. Lamp irradiation in pond water, continuous decoupled spectrum.

Liquid state IGD and DEPT ^15^N NMR spectra of the sunlight photolysate are shown in Fig 11C and S10 Fig, respectively, while ACOUSTIC and continuous decoupled spectra of the photolysates from sunlight and lamp irradiations are compared in Fig 25. Nitrite (614.8 ppm) is observed in the spectra of both photolysates whereas ammonium is not (Fig 25). Ammonia may be lost through volatilization, or recondensation with either the degradation products or the background NOM, processes favored at the slightly alkaline pH of the irradiation. All three liquid state spectra show peaks from 60 to 170 ppm in the region containing aromatic amine, 1° amide, and 2° amide/PHA nitrogens, as well as the residual nitro groups. A very low intensity broad peak is barely visible at 304.5 ppm in the IGD spectrum (Fig 11C) and 300.0 ppm in the continuous decoupled spectrum (Fig 25A) of the sunlight photolysate. These peaks are unassigned but occur in the region of pyridine- or indazole (N2)-like nitrogens. They are reminiscent of the peaks detected in this region in spectra of the 2ADNT and 4ADNT photolysates (S8 Fig). The most notable feature of the IGD and continuous decoupled spectra of the pond water photolysates is the absence of well defined azoxy and azo nitrogen peaks. Possible matches for 3,5-dinitroaniline (peaks at 68.8 and 365.9 ppm) and 2ADNT (peaks at 70.8, 366.7, and 374.2 ppm) are evident in Figs 14 and 15.

Azo nitrogens likewise are not observed in the CP/MAS ^15^N NMR spectra of either pond water photolysate, whereas a low intensity azoxy peak is present at 322 ppm in the lamp photolysate but not the sunlight photolysate (Fig 24B and 24C). It can therefore be concluded that condensation reactions via azoxy and azo nitrogen group formation are inhibited in the pond water, compared to solid phase sunlight irradiation, and aqueous phase irradiation in deionized water. Similar to the liquid state spectra, the CP/MAS spectra do show the major peaks from approximately 50 to 220 ppm corresponding to aromatic amine, 1° amide, and 2° amide/PHA nitrogens, the latter again the peaks of maximum intensity in this region. The residual nitro nitrogens are present at 366 ppm. Of note are the resolved peak at 185 ppm (possibly indazole) in the lamp photolysate, and the low intensity broad peaks at 281 (possibly nitrone) and 299 ppm (pyridine-like) in the sunlight photolysate. Nitrite peaks observed in the liquid state spectra are not observed in the solid state spectra. Consistent with the liquid state spectra, ammonium peaks are not observed in the solid state spectra. To determine if the inhibition of azo and azoxy formation in the pond water could be attributed to the alkaline pH or the presence of NOM, experiments were conducted to control for these factors, including irradiations in the presence of NOM and in pH 8 buffer, and a dark control for alkaline hydrolysis.

### Effect of NOM on photolysis of T^15^NT in the aqueous phase

**Fig 26:**
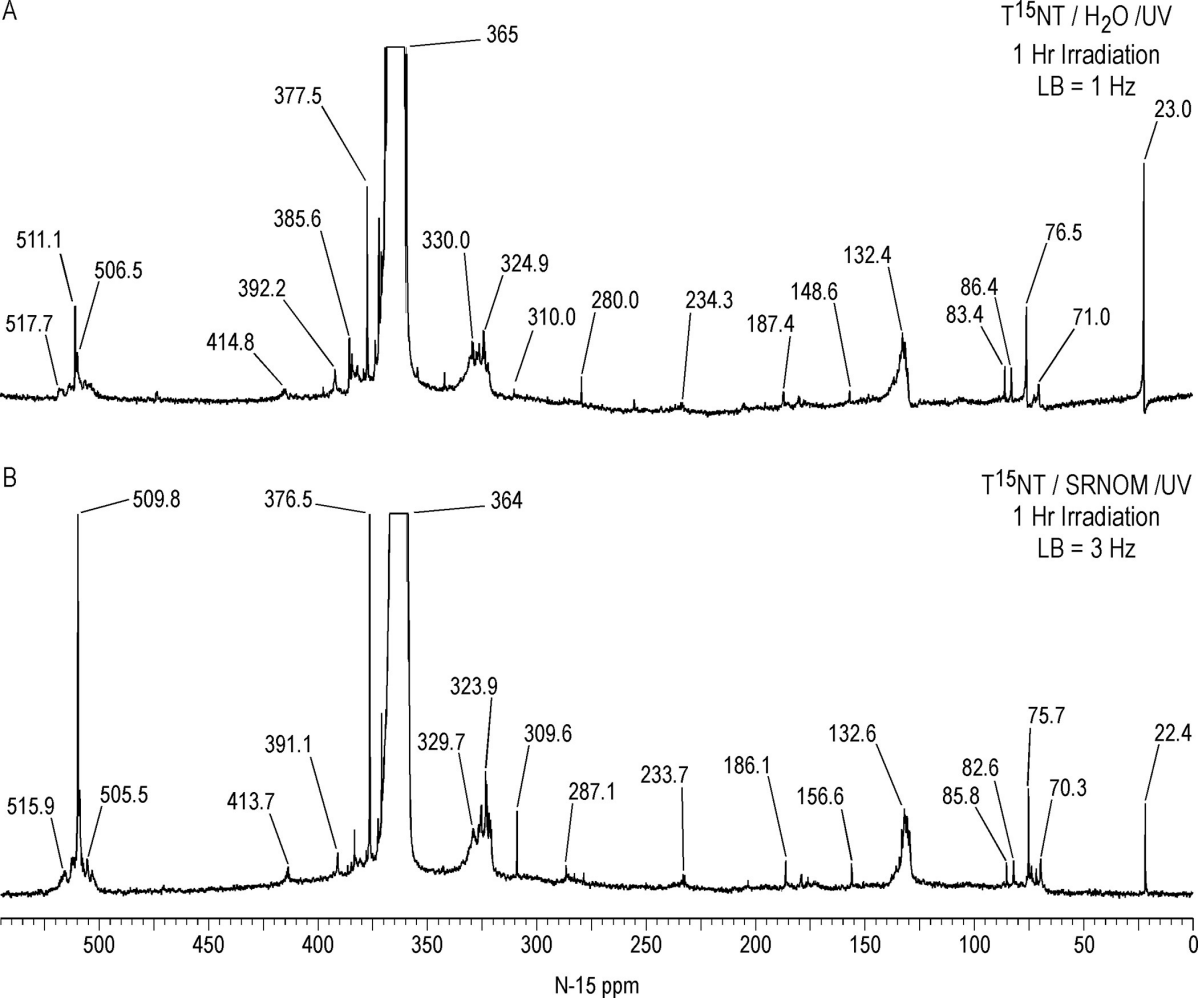
Liquid state IGD N-15 NMR spectra of T^15^NT. A. Lamp irradiation in deionized water. Lamp irradiation in deionized water with Suwannee River NOM added. LB = line broadening.

T^15^NT was irradiated in the presence of Suwannee River NOM (SRNOM) at a concentration of 27.8 mg C/L NOM, the initial pH of 3.7 dropping to a final value of 3.2. Liquid state IGD ^15^N NMR spectra of the 1 hour photolysates of T^15^NT in deionized water and in the presence of SRNOM, both irradiated with the lamp at similar concentrations of the explosive (111 mg/L), are compared in Fig 26. The spectra are remarkably similar, showing comparable distributions of aromatic amine, 2° amide/PHA, azoxy, nitro, nitrosophenol, and azo nitrogens, among other functional groups. Thus, at the pH and concentrations of this experiment, the NOM does not appear to affect the distribution of transformation products, and does not inhibit condensation via azo and azoxy formation. A synergistic effect of NOM with alkaline conditions in terms of kinetics and product distribution cannot be ruled out however. A notable feature in the spectrum of the T^15^NT/SRNOM photolysate is the signal intensity of the N_2_ gas peak at 309.6 ppm.

### Aqueous phase irradiation of TNT in pH 8 buffer solution

**Fig 27:**
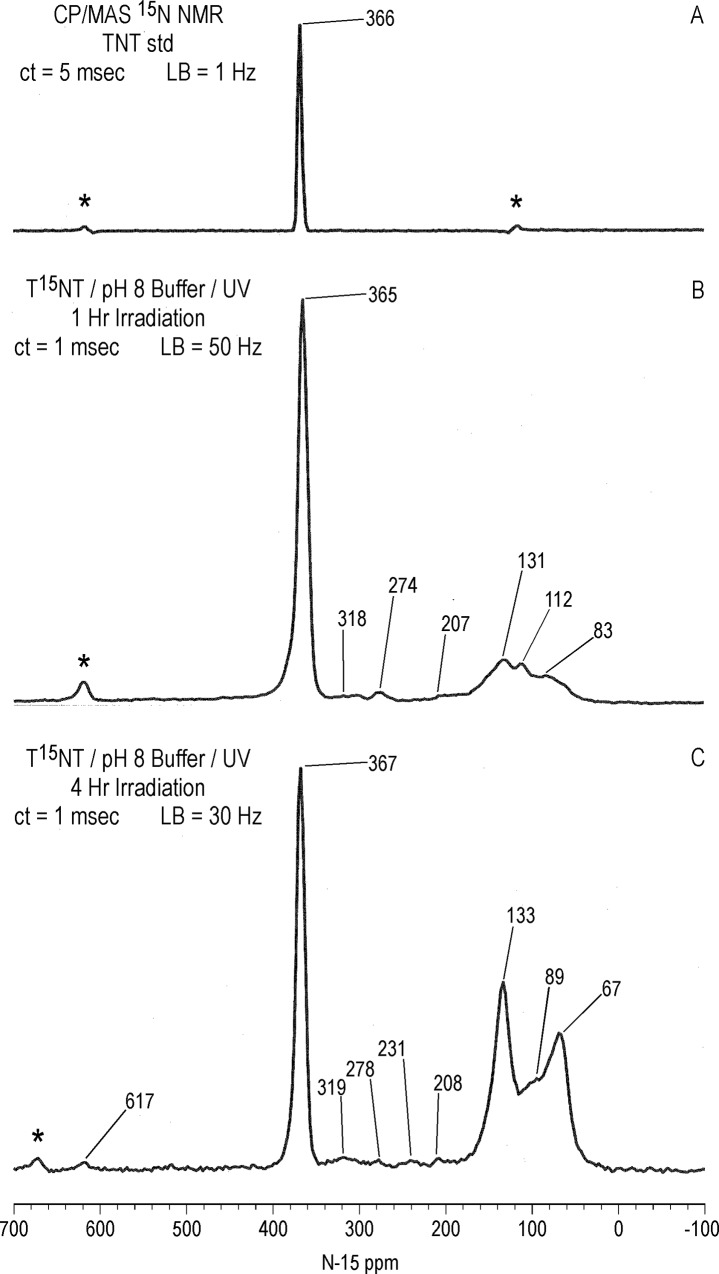
Solid state CP/MAS N-15 NMR spectra of T^15^NT. A. Standard. B. 1 hour lamp irradiation in pH 8 buffer solution. C. 4 hour lamp irradiation in pH 8 buffer solution. ct = contact time. LB = line broadening. SS = spinning speed. Asterisk = spinning side band.

Lamp irradiations were performed on solutions of T^15^NT dissolved in pH 8 buffer for periods of 1 and 4 hours. Solid state ^15^N and ^13^C NMR spectra were recorded on the freeze dried powders containing the photolysate and buffer salts (Fig 27 and S11 Fig, respectively). In these experiments, because complete separation of the transformation products from the buffer salts was impractical, liquid state ^15^N analyses, which thusfar have been shown advantageous for detection of azo nitrogens, were not performed. The CP/MAS ^15^N NMR spectra do reveal that azoxy nitrogens are detectable in the 4 hour, but not the 1 hour photolysate, in the former case only a very low intensity peak at 319 ppm (Fig 27). Azo nitrogens are not observed in either photolysate. The spectra otherwise are consistent with the solid state ^15^N NMR spectra of the T^15^NT photolysates from the deionized water, pond water, and solid state matrices in that they exhibit the broad band from 50 to 220 ppm containing aromatic amine and amide nitrogens, with peak maxima at 131 to 133 ppm corresponding to the 2° amide/PHA nitrogens. The ratio of nitro to other nitrogen peak intensities decreases from 1 to 4 hours, consistent with an increase in transformation products with time. Both spectra have shoulders at 207–208 ppm (possibly imidazole, pyrazole, amidine, or imidate nitrogens) and low intensity peaks at 274 to 278 ppm (possibly nitrones). There is also a low intensity peak at 231 ppm in the 4 hour photolysate, possibly diazonium or azo-hydrazone nitrogens. The intensity of the aromatic amine peak at 67 ppm in the 4 hour photolysate is notable. A nitrite peak is visible at 617 ppm in the 4 hour photolysate. (As the spectrum of the 1 hour photolysate was recorded with a spinning speed of 5 KHz, if present, nitrite would overlap with the downfield first order spinning sideband to the nitro peak.) Ammonium peaks are not observed in either spectrum. Although less satisfactory than liquid state ^15^N NMR analysis, solid state analysis confirms that formation of azo and azoxy compounds is inhibited at alkaline compared to acidic pH.

### Dark control for alkaline hydrolysis

**Fig 28:**
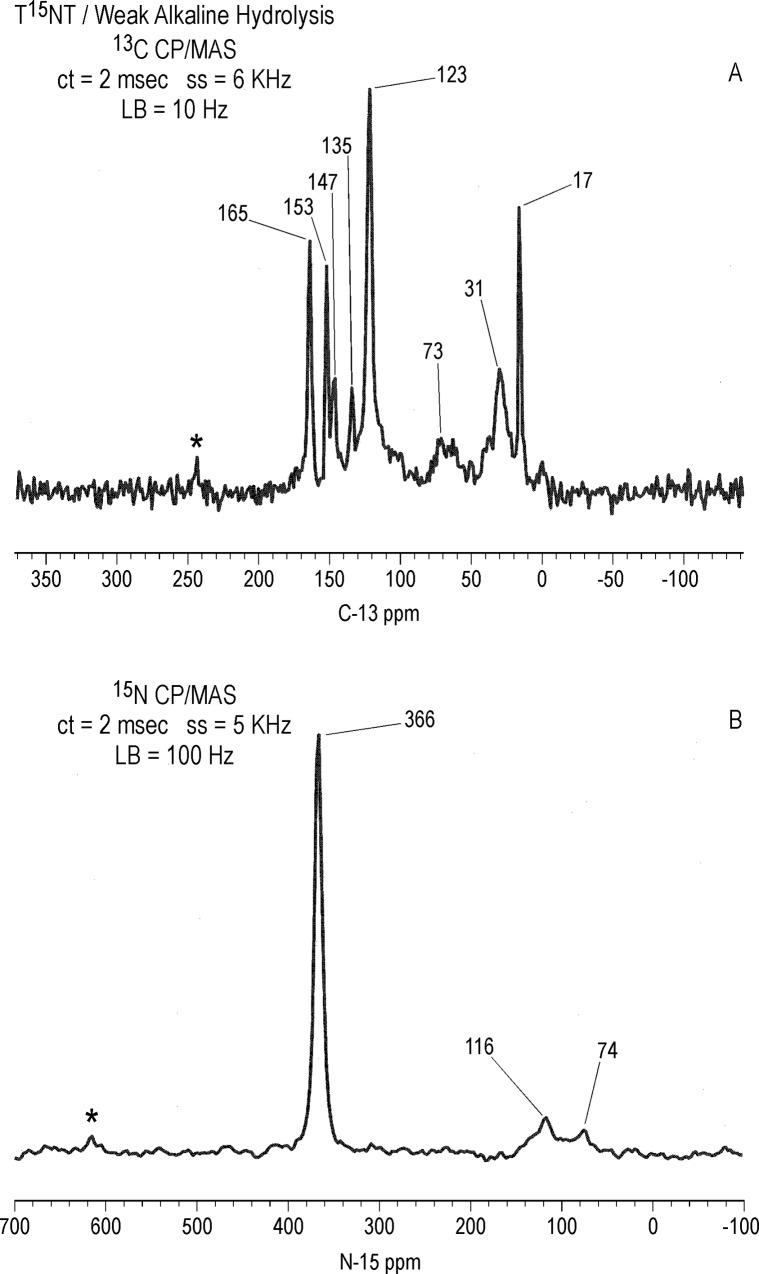
Solid state CP/MAS spectra of T^15^NT subjected to weak alkaline hydrolysis as dark control reaction. A. C-13 NMR. B. N-15 NMR. ct = contact time. LB = line broadening. SS = spinning speed. Asterisk = spinning side band.

T^15^NT was subjected to a dark control experiment to determine the extent of transformation under conditions of weak alkaline hydrolysis. The initial pH of 9.0 dropped to 7.7 over the course of the 8 day treatment. The length of exposure of the T^15^NT to alkaline conditions was 4 times that of the sunlight irradiation in pond water, 192 times the lamp irradiation in pond water, 192 times the 1 hour lamp irradiation in pH 8 buffer, and 48 times the 4 hour lamp irradiation in pH 8 buffer. The solid state CP/MAS ^13^C NMR spectrum shows methylene carbons at 31 ppm, O-alkyl carbons at 73 ppm, and a bicarbonate and/or carboxyl peak at 165 ppm (Fig 28A). The methylene carbons again may be attributable to structures arising from nucleophilic addition of the 2,4,6-trinitrobenzyl anion to the C_3_ carbon of a second TNT molecule. The CP/MAS ^15^N NMR spectrum (Fig 28B) shows low intensity peaks at 74 ppm (1° aromatic amine or 2° anilinohydroquinone) and 116 ppm (1° amide or 2° anilinoquinone)[87]. Considering the excess length of exposure to alkaline conditions of the dark control over the irradiation experiments, and the relatively minor degree of transformation in the dark, especially with respect to nitrogen, it is unlikely that the transformation of TNT during irradiation in the pond water and in the pH 8 buffer that can be attributed to hydrolysis alone is significant. The intensities of the peaks corresponding to transformation products are much less than in previously reported spectra of T^15^NT treated with alkali at initial pH of approximately 12, where the objective of the hydrolysis was remedial destruction of the TNT [87].

From this and the previous experiments then, the inhibition of azo and azoxy formation in the pond water under both sunlight and lamp irradiation can most likely be attributed to an effect of pH and not NOM. This effect would also likely be relevant in the case of leaking munitions in shallow marine environments of alkaline pH. Compared to conditions at neutrality or above, nitrite released from TNT at acidic pH presumably has a greater potential for reincorporation into the transformation products via nitrosation reactions, including formation of aromatic nitroso groups that have the potential to condense with aromatic amines or phenylhydroxylamines to form the azo and azoxy ccompounds. This may be a contributing circumstance to the relative inhibition at alkaline pH, but a further mechanistic investigation is required.

### Aqueous phase irradiation of 2,6-dinitrotoluene in deionized water

Liquid state ^13^C and solid state CP/MAS ^15^N NMR spectra were recorded on the photolysates from 4.5 hour unfiltered and 4 hour filtered lamp irradiations of unlabeled 2,6-DNT in deionized water. The irradiation times of these initial experiments were chosen based upon color formation judged sufficient for detection of transformation products.

**Fig 29:**
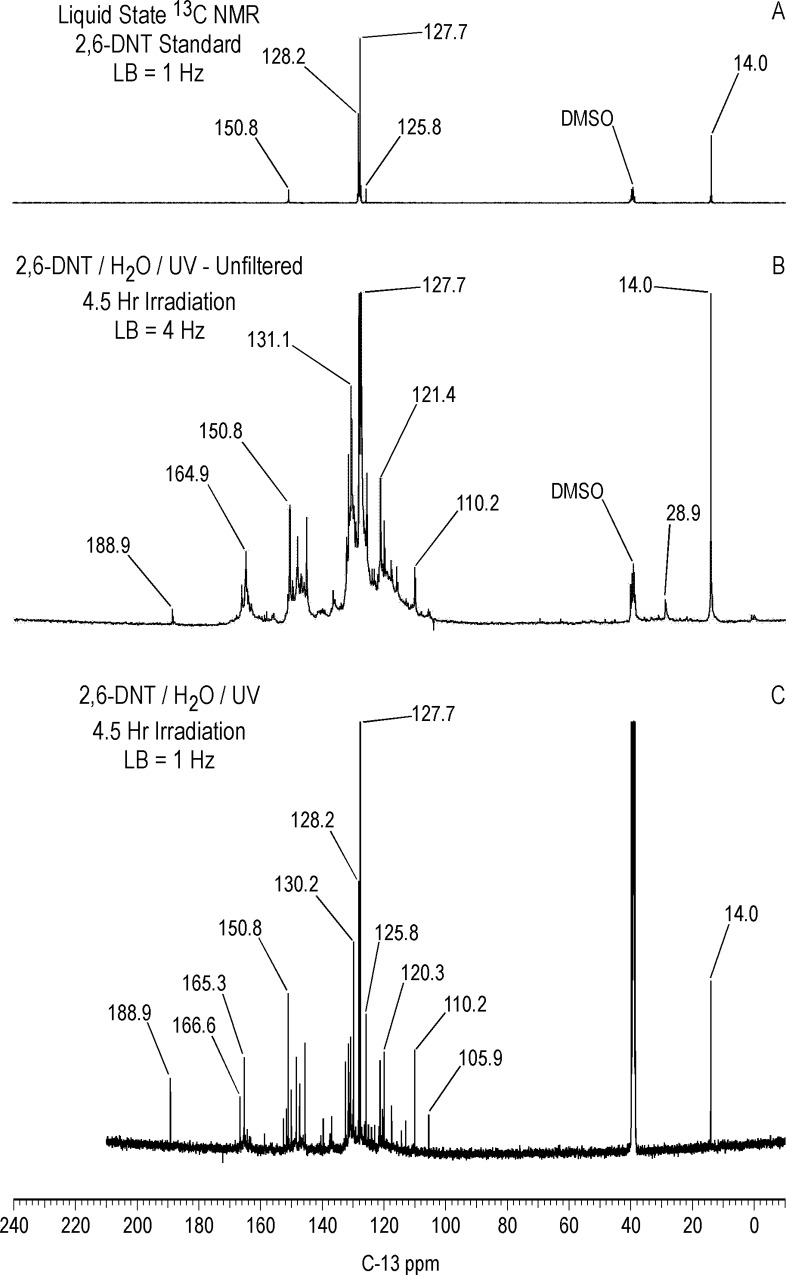
Liquid state continuous decoupled C-13 NMR spectra of 2,6-DNT (2,6-dinitrotoluene; unlabeled). A. Standard. B. Unfiltered lamp irradiation in deionized water. C. Pyrex-filtered lamp irradiation in deionized water. LB = line broadening.

Assignments for the liquid state ^13^C NMR spectrum of the 2,6-DNT standard in dmso-d_6_ are: C_1_ = 128.2 ppm; C_2&6_ = 150.8 ppm; C_3&5_ = 127.7 ppm; C_4_ = 125.8 ppm; methyl = 14.0 ppm (Fig 29A). The liquid state ^13^C NMR spectrum from the unfiltered irradiation shows the aldehyde carbon to 2,6-dinitrobenzaldehyde at 188.9 ppm, carboxyl/amide carbons from 160 to 170 ppm, and a series of unassigned peaks from 100 to 125 ppm. A methylene carbon peak is present at 28.9 ppm, and residual methyl carbons at 14.0 ppm (Fig 29B). The spectrum from the filtered irradiation also shows the aldehyde carbon to 2,6-dinitrobenzaldehyde at 188.9 ppm, carboxyl/amide carbons from 160 to 170 ppm, and peaks from the residual TNT at 14.0, 125.8, 127.7, 128.2, and 150.8 ppm (Fig 29C). The spectrum does not show evidence for the alcohol carbon to 2,6-dinitrobenzylic alcohol that would occur at approximately 55.5 ppm.

**Fig 30:**
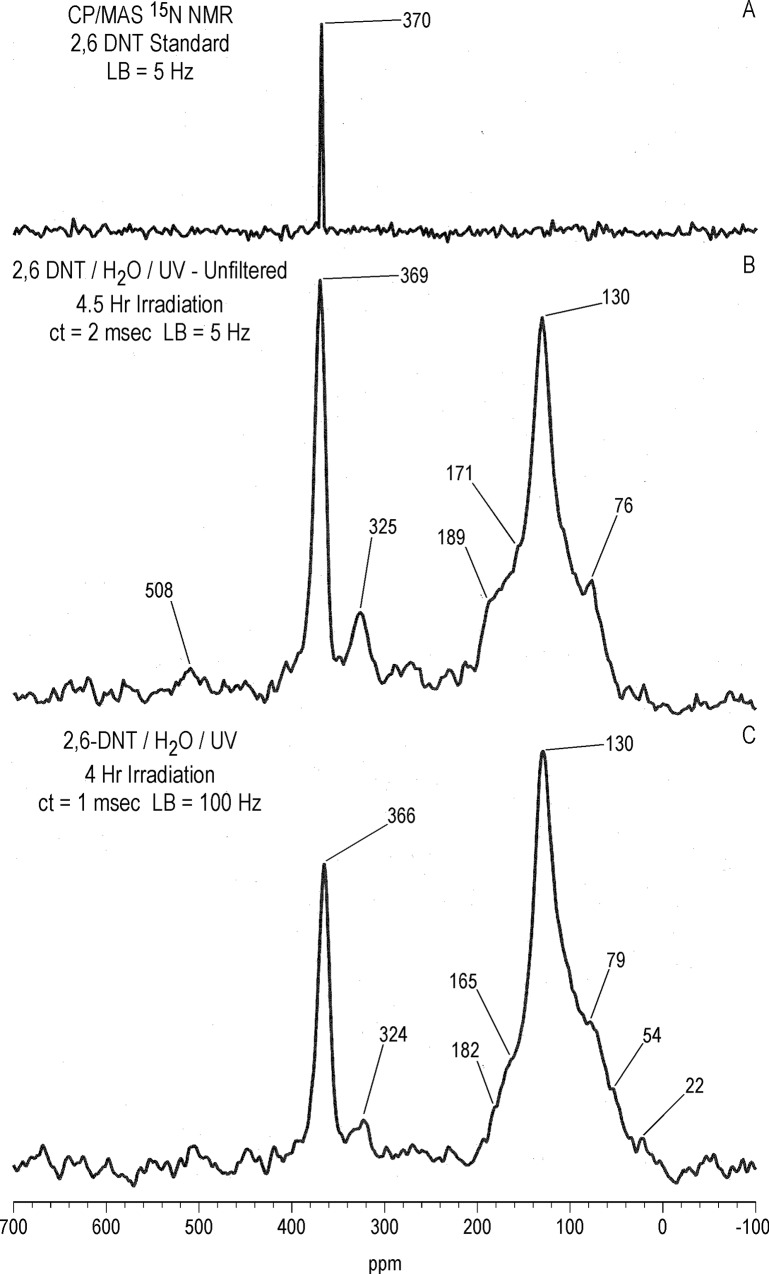
Solid state CP/MAS N-15 NMR spectra of 2,6-DNT (unlabeled). A. Standard. B. Unfiltered lamp irradiation in deiononized water. C. Pyrex-filtered lamp irradiation in deionized water. ct = contact time. LB = line broadening. SS = spinning speed.

^15^N NMR analyses of the 2,6DNT photolysates were limited to the solid state CP/MAS experiment as the ^15^N-labeled compound is unavailable commercially. The nitro peak of the standard occurs at 370 ppm (Fig 30A). The solid state CP/MAS ^15^N NMR spectrum from the unfiltered irradiation (Fig 30B) shows the familiar broad band from 50 to 220 ppm with maximum at 130 ppm in the 2° amide/PHA region along with a resolved aromatic amine peak at 76 ppm, and residual nitro peak at 369 ppm. There is a well resolved azoxy peak at 325 ppm and a weak azo peak at 508 ppm. A resolved ammonium peak is not visible in this spectrum.

The solid state CP/MAS ^15^N NMR spectrum from the Pyrex–filtered irradiation (Fig 30C) shows the familiar broad band from 50 to 220 ppm with peak maximum at 130 ppm in the 2° amide/PHA region. The 2,6DNT photolysate also shows an ammonium peak at 22 ppm, an azoxy peak at 324 ppm, and the residual nitro peak at 366 ppm. Azo nitrogens are not observed at the signal to noise level achieved for this spectrum. The ^15^N NMR spectra of the photolysates from the filtered and unfiltered irradiations were recorded with contact times of 1-msec and 2-msec, respectively, and so are not directly comparable in terms of peak intensities.

These analyses indicate general similarities in the transformation pathways of 2,6DNT and TNT in deionized water, consistent with the literature [34], and again highlighting the potential significance of benzanilide formation as a condensation pathway. It would seem reasonable to extrapolate from the TNT analyses and predict that sunlight irradiation of 2,6-DNT in the solid phase produces classes of degradation products similar to those detected in deionized water, and that azoxy- and azo- formation is inhibited during irradiation of 2,6-DNT in aqueous solution at pH above neutrality.

## Discussion

### Transformation products and reaction chemistry

The starting point for this study was the application of ^13^C and ^15^N NMR to the experiments of Burlinson and Kaplan [21, 33, 34, 36, 37]. Of the classes of TNT phototransformation products they identified (Table 1), the NMR spectra provide evidence for aldehyde, carboxylic acid, aromatic amine, benzanilide, nitrile, azoxy, nitro, and azo compounds. Because their ^15^N NMR chemical shifts overlap with nitro groups, the benzisoxazole and benzaldoxime compounds cannot be directly identified from the ^15^N NMR spectra. However, as precursors to nitriles and primary amides, the formation of benzaldoxime compounds during irradiation can be inferred. A main prediction of Burlinson and Kaplan was that the insoluble reddish-brown residue formed from irradiation of TNT in distilled water was comprised of oligomers of azoxy and azo compounds. The detection of clusters of azoxy and azo resonances in the ^15^N NMR spectra of the TNT photolysates from deionized water are consistent with this pathway of dimerization and oligomerization, with the caveat that imine and azoxy nitrogens overlap, and the extent of imine formation via condensation of aromatic amines with benzaldehydes cannot be determined directly from the ^15^N NMR. In addition to providing evidence for phototransformation products not identified by other analytical techniques before, such as primary amide, diphenylamine, hydrazobenzene, 1° aminoquinone, and nitrosophenol compounds (summarized in Table 6), the NMR analyses reveal more numerous constituents in each of the aromatic amine, benzanilide, nitrile, azoxy and azo compound classes than previously realized. In particular, the potential significance of benzanilide formation as a dimerization mechanism has not been previously recognized. The relative contribution of phenylhydroxylamines to the 2° amide/PHA peaks is uncertain, but if PHAs can be confirmed, then their occurrence as stable phototransformation products would likewise be new information. The ^13^C NMR spectra also provide possible evidence for condensation reactions via nucleophilic addition of the 2,4,6-trinitrobenzyl anion to the C_3_ carbon of TNT or stilbene formation. Azo-hydrazone, nitrone, diazonium, and oxaziridine compounds all have ^15^N NMR chemical shifts that coincide with peaks in the ^15^N NMR spectra, and may be considered plausible phototransformation products as well, although the latter two are generally considered reactive intermediates. The limited analyses of 2,6-dinitrotoluene irradiated in deionized water suggest similar photolysis pathways and classes of transformation products.

**Table 6:** Previously unreported or underestimated TNT phototransformation products detected by N-15 NMR.

Transformation Product	Matrix	Figure	Peaks
Diphenylamine or Hydrazobenzene	Solid TNT/Sunlight/9 day	22B	84.0, 86.5, 87.1
1° Aminoquinone	TNT/H_2_O/UV/1 hr	13	83.5, 95.7
	Solid TNT/Sunlight/9 day	22A	80–100
	TNT/Pond Water/Sunlight/2 day	S10	82.5, 87.6, 94.6
1° Amide	TNT/H_2_O/UV/1 hr	13	102–115
	Solid TNT/Sunlight	22	102–115
	TNT/Pond Water/Sunlight/2 day	S10	102.5, 108.4, 109.2, 113.1
	TNT/H_2_O/UV-unfil/1 hr	S7A	103.4
	TNT/H_2_O/UV-unfil/16 hr	S7B	105.9
Phenylhydroxylamine or Benzanilide	TNT/H_2_O/UV/1 hr	11B	132.4
	TNT/H_2_O/UV-unfil/1 hr	S6A	131.0
	Solid TNT/Sunlight/9 day	21B	131.2
	TNT/Pond Water/Sunlight/2 day	11C	134.5
	TNT/H_2_O/SRNOM/UV 1 hr	26B	132.6
	TNT/pH 8 Buffer/ 1 hr	27B	131
Indazole (>NH-)	TNT/H_2_O/UV/1 hr	12B, S5	180.2, 187.4
	TNT/H_2_O/UV/ 4 hr	17B	183
	TNT/H_2_O/Sunlight/2 day	17C	185
	TNT/Pond Water/UV/1 hr	24C	185
Diazonium (N1) or Azo-hydrazone (PhNH-)	TNT/H_2_O/UV/1 hr	12B, S5	234.3
	TNT/H_2_O/UV/ 4hr	17B	236
	TNT/pH 8 Buffer/ 4hr	27C	231
Benzonitrile	TNT/H_2_O/Sunlight/2 day	17C	266
	TNT/H_2_O/UV-unfil/16 hr	S7B	267.4
Nitrone	TNT/H_2_O/UV/1 hr	12B, S5	280.0
	TNT/Pond Water/Sunlight/2 day	24B	281
N_2_	TNT/H_2_O/UV/1 hr	12	310.0
	TNT/H_2_O/SRNOM/UV 1 hr	26	309.6
Nitrosophenol	TNT/H_2_O/UV/1 hr	16A	392.2, 414.4
	Solid TNT/Sunlight/9 day	16B	391.9, 395.4, 404.4

This study indicates that a further understanding of TNT photochemistry requires significantly more research in terms of identification of structures and elucidation of reaction mechanisms. Priorities would include confirmation of, and resolution between, benzanilide and phenylhydroxylamine structures, and a more detailed mechanistic explanation for the inhibition of azoxy and azo formation at alkaline pH. Additional needs include a further refinement of the effects of reaction time, concentration, pH, and the role of NOM throughout a range of pH, the role of hydroxyl radical, nitric oxide, and nitrogen dioxide in the transformation reactions, and synthesis of key transformation products for NMR chemical shift determination and mass spectrometric analysis. ESR analyses would also provide insight into the role of free radical catalyzed transformation reactions.

In terms of the matrix effects examined here, the distribution of transformation products from sunlight irradiation of TNT in the solid state most closely resembles the distribution from both sunlight and lamp irradiation in deionized water. The most significant difference is the inhibition of azo and azoxy formation observed at alkaline pH in the pond water and pH 8 buffer. One of the most important matrix effects thus appears to lie within the dimerization and oligomerization pathways. If confirmed, benzanilide formation would comprise a dimerization mechanism important in all matrices.

### Practical aspects of NMR

The analyses presented here serve as a case study illustrating the complementary use of liquid and solid state NMR for ^13^C and ^15^N nuclei in a multicomponent mixture containing monomers and oligomers of aromatic compounds, and the varying ability of individual pulse sequences within liquid state ^15^N NMR for detecting particular nitrogens. Practical observations are summarized here. In general, the superior resolution attainable in liquid state analyses provides critical information not available in the CP experiment due to line broadening mechanisms in the solid state. Examples include the detection of discrete peaks attributable to the aldehyde carbon of 2,4,6-trinitrobenzaldehyde, nitrate and N_2_ gas nitrogens, and the numerous aromatic amine, amide, and nitro nitrogens in the liquid state spectra. Detection of nitrite in the liquid but not the solid state ^15^N NMR spectra of the pond water irradiations is another example. This is due in part due to the broad linewidth of nitrite in the solid state [87]. Both azo and azoxy nitrogens are more readily detected by liquid than solid state ^15^N NMR. Conversely, in a couple of instances the CP experiment shows peaks absent in the liquid state spectra, or significantly enhances signals over the liquid state experiments. For example, the residual methyl carbon to TNT was detected in solid but not liquid state ^13^C spectra of the sunlight irradiations of TNT in the solid state and in pond water (Figs 11 and 20, and S9 Fig). The CP experiment significantly enhanced the methylene carbons at approximately 30 ppm, allowing the formation of these to be monitored as a function of irradiation time or as a component in precipitate formation. An obvious advantage of solid state NMR is the ability to directly analyze photolysates in a complex matrix such as the pond water, and detect nitrogens in product mixtures from unlabeled substrates such as the 2,6DNT.

Within liquid state ^15^N NMR, the polarization transfer experiment enables the visualization of signals not apparent from the single pulse experiments. For example, the DEPT spectrum of the TNT subjected to the lamp irradiation in deionized water shows significant enhancement of signals from 90 to 130 ppm over the single pulse experiment, including discrete resonances at 103.7, 107.4, 110.9, and 113.7 ppm not visible in the single pulse experiment (Figs 12 and 13). On the other hand, some aromatic amine resonances present in spectra from single pulse experiments, such as those at 70.8 and 76.5 ppm from the lamp irradiation of TNT in deionized water (Fig 12) and 70.0 and 75.0 ppm from the sunlight irradiation of solid TNT (Fig 23), are not detected in DEPT spectra because of exchange.

## Supporting information

S1 Table Carbon-13 NMR chemical shifts of monomeric TNT, 2,6DNT, and 2,4DNT transformation products. (DOCX)

S1 Text Unfiltered Irradiation of T^15^NT in deionized water. (DOCX)

S2 Text Solid State CP/MAS ^13^C NMR Spectra of Pond Water Solids and Photolysates of T^15^NT in Pond Water. (DOCX)

S1 Fig Structures of reported TNT photodegradation products. (TIF)

S2 Fig Pathway of TNT photodegradation according to Spanggord (ref. 53). (TIF)

S3 Fig Liquid state continuous decoupled C-13 NMR spectrum of T^15^NT subjected to lamp irradiation in deionized water. Horizontal Expansion. Line Broadening = 1.0 Hz.
(TIF)

S4 Fig Solid state CP/MAS C-13 NMR spectra. A. Precipitate fraction of TNT subjected to 1-hour lamp irradiation in deionized water. B. T^15^NT subjected to 1 hour of unfiltered lamp irradiation in deionized water. ct = contact time. LB = line broadening. SS = spinning speed. Asterisk = spinning sideband. Double asterisk = contaminant.
(TIF)

S5 Fig Liquid state IGD N-15 NMR spectra of T^15^NT subjected to lamp irradiation in deionized water for 1 hour. Horizontal and vertical scale expansions.
(TIF)

S6 Fig Liquid state IGD N-15 NMR spectra of T^15^NT. On scale. A. 1-hour unfiltered lamp irradiation in deionized water. B. 16-hour unfiltered lamp irradiation in deionized water. LB = line broadening.
(TIF)

S7 Fig Liquid state IGD N-15 NMR spectra of T^15^NT. Vertical scale expansion. A. 1-hour unfiltered lamp irradiation in deionized water. B. 16-hour unfiltered lamp irradiation in deionized water. LB = line broadening.
(TIF)

S8 Fig Liquid state continuous decoupled N-15 NMR spectra.of lamp irradiated 4-^15^N-amino-2,6-dinitrotoluene (2 hour irradiation) and 2-^15^N-amino-4,6-dinitrotoluene (1 hour irradiation) in deionized water. Vertical and horizontal scale expansion. LB = line broadening.
(TIF)

S9 Fig Solid state CP/MAS C-13 NMR spectra. A. Pond water solids. B. Sunlight irradiation of T^15^NT in pond water. C. Lamp irradiation of T^15^NT in pond water. ct = contact time. LB = line broadening. SS = spinning speed. Asterisk = spinning sideband.
(TIF)

S10 Fig Liquid state N-15 NMR DEPT spectrum of T^15^NT subjected to sunlight irradiation in pond water. Nitrogens bonded to two protons in negative phase and nitrogens bonded to one nitrogen in positive phase. LB = line broadening.
(TIF)

S11 Fig Solid state CP/MAS C-13 NMR spectra of T^15^NT. A. Standard. B. 1-hour lamp irradiation in pH 8 buffer solution. C. 4-hour lamp irradiation in pH 8 buffer solution. ct = contact time. LB = line broadening. SS = spinning speed. Asterisk = spinning sideband. Double asterisk = contaminant.
(TIF)
