# Supplementary material for: 13C and 15N NMR identification of product compound classes from aqueous and solid phase photodegradation of 2,4,6-trinitrotoluene
Source: PLoS One. 2019 Oct 22;14(10):e0224112. doi: 10.1371/journal.pone.0224112 (PMC6804990; doi:10.1371/journal.pone.0224112)
Supplement: S1 Table — (DOCX) [file pone.0224112.s001.docx]

Table S1. Carbon-13 NMR chemical shifts of monomeric TNT, 2,6DNT, and 2,4DNT transformation products.

| Compound | Cα | C1 | C2 | C3 | C4 | C5 | C6 | C3a | C7 | C7a |
| --- | --- | --- | --- | --- | --- | --- | --- | --- | --- | --- |
| 2,4,6-trinitro  toluene ^a^ | 15.0 | 132.9 | 150.8 | 122.5 | 145.6 | 122.5 | 150.8 |  |  |  |
| 2,4,6-trinitro-benzylic alcohol ^b^ | 55.5 | 137.2 | 149.2 | 123.3 | 146.1 | 123.3 | 149.2 |  |  |  |
| 2,4,6-trinitro-benzaldehyde ^a^ | 187.9 | 134.2 | 148.1 | 124.6 | 147.6 | 124.6 | 148.1 |  |  |  |
| 2,4,6-trinitro-benzoic acid ^a^ | 162.2 | 129.6 | 146.6 | 124.9 | 147.4 | 124.9 | 146.6 |  |  |  |
| 2-amino-4,6-dinitrotoluene ^a^ | 13.0 | 120.6 | 150.0 | 109.9 | 146.0 | 104.8 | 151.1 |  |  |  |
| 4-amino-2,6-dinitrotoluene ^a^ | 13.1 | 110.3 | 151.8 | 111.8 | 148.4 | 111.8 | 151.8 |  |  |  |
| 4-hydroxyl-amino-2,6-dinitrotoluene ^b^ | 15.1 | 121.9 | 148.0 | 115.5 | 149.6 | 115.5 | 148.0 |  |  |  |
| 2,4,6-trinitro-benzamide ^b^ | 168.0 | 117.2 | 147.9 | 126.2 | 145.8 | 126.2 | 147.9 |  |  |  |
| 2,4,6-trinitro-  Benzonitrile ^b^ | 115.8 | 108.8 | 150.1 | 126.8 | 151.8 | 126.8 | 150.1 |  |  |  |
| 2,4-dinitro-toluene ^c^ | 18.3 | 139.5 | 150.2 | 116.9 | 145.8 | 129.9 | 130.8 |  |  |  |
| 2,4-dinitro-benzylic alcohol ^d^ | 61.8 | 145.8 | 145.8 | 121.1 | 145.8 | 129.1 | 129.1 |  |  |  |
| 2,4-dinitro-benzaldehyde ^d^ | 187.3 | 137.1 | 150.3 | 120.7 | 150.3 | 130.4 | 130.4 |  |  |  |
| 2,4-dinitro-benzoic acid ^a^ | 164.9 | 132.8 | 147.9 | 119.5 | 148.7 | 128.0 | 131.5 |  |  |  |
| 2,6-dinitro-toluene ^a^ | 14.0 | 125.8 | 150.8 | 127.7 | 128.2 | 127.7 | 150.8 |  |  |  |
| 2,6-dinitro-benzylic alcohol ^b^ | 55.5 | 131.1 | 148.3 | 131.2 | 126.9 | 131.2 | 148.3 |  |  |  |
| 2,6-dinitro  benzaldehyde ^a^ | 188.9 | 130.1 | 147.2 | 130.0 | 132.7 | 130.0 | 147.2 |  |  |  |
| 2,6-dinitro-benzoic acid ^a^ | 163.4 | 125.4 | 146.4 | 130.0 | 131.9 | 130.0 | 146.4 |  |  |  |
| 2-amino-4,6-dinitrobenzoic acid ^a^ | 165.3 | 114.2 | 151.0 | 111.3 | 150.1 | 104.2 | 148.6 |  |  |  |
| 4-amino-2,6-dinitrobenzoic acid ^a^ | 163.9 | 109.8 | 148.9 | 111.2 | 151.3 | 111.2 | 148.9 |  |  |  |
| 3,5-dinitro-aniline ^e^ |  | 1501.2 | 112.3 | 148.7 | 103.6 | 148.7 | 112.3 |  |  |  |
| 3,5-dinitro-phenol ^e^ |  | 159.1 | 115.8 | 148.7 | 108.5 | 148.7 | 115.8 |  |  |  |
| 1,3,5-trinitro-benzene^a^ |  | 148.3 | 123.9 | 148.3 | 123.9 | 148.3 | 123.9 |  |  |  |

| Compound | Cα | C1 | C2 | C3 | C4 | C5 | C6 | C3a | C7 | C7a |
| --- | --- | --- | --- | --- | --- | --- | --- | --- | --- | --- |
| 1,3-dinitro-benzene ^e^ |  | 148.0 | 118.5 | 148.0 | 129.3 | 131.6 | 129.3 |  |  |  |
| 4,6-dinitro-1,2-benzisoxazole ^b^ |  |  |  | 147.1 | 145.1 | 113.2 | 151.4 | 123.4 | 111.1 | 166.7 |
| 4,6-dinitro-2,1-benzisoxazole ^b^ |  |  |  | 152.4 | 149.3 | 120.0 | 135.8 | 100.5 | 113.7 | 162.0 |

^a^ Recorded in this laboratory in DMSO-d_6_

^b^ Predicted from Chemdraw

^c^ Aldrich NMR Library, in CDCl_3_

^d^ Recorded in this laboratory in the solid state

^e^ Aldrich NMR Library, in DMSO-d_6_
