# Supplementary material for: 13C and 15N NMR identification of product compound classes from aqueous and solid phase photodegradation of 2,4,6-trinitrotoluene
Source: PLoS One. 2019 Oct 22;14(10):e0224112. doi: 10.1371/journal.pone.0224112 (PMC6804990; doi:10.1371/journal.pone.0224112)
Supplement: S1 Text — (DOCX) [file pone.0224112.s002.docx]

**S1 Text**

**Unfiltered Irradiation of T^15^NT in Deionized Water.**

Unfiltered UV irradiation in conjunction with advanced oxidation catalysts has been studied for destruction of TNT in contaminated water. An understanding of how degradation proceeds without catalysts is desirable as a control reaction in this context. It is therefore of interest to compare spectra of the photolysates generated with and without Pyrex-filtering of the medium pressure lamp. As light absorption of TNT is strongest at approximately 235 nm, removal of the Pyrex filter would be expected to enhance transformation and degradation.

The liquid state ^15^N NMR spectra of the filtered (Fig 11B) and unfiltered 1 hour photolysates (S6A Fig) show general similarities in the distributions and concentrations of transformation products, but with fewer discreet resonances in the unfiltered photolysate. A number of changes are visible in the spectra of the photolysates from 1 to 16 hours of unfiltered irradiation (S6-S7 Fig). The ratio of nitro to other nitrogen peaks decreases, indicating an overall transformation of nitro groups to other nitrogen functionalities, including photochemical mineralisation to ammonium (22.1 ppm). There is a significant increase in the intensity of peaks in the region from 50 to 180 ppm, encompassing aromatic amine, 1° amide, and 2° amide/PHA nitrogens, along with the appearance of discrete peaks at 70.4, 75.8, 102.2, and 105.7 ppm. There is also an increase in the peaks at 251.3 and 267.5 ppm, the latter encompassing benzonitriles. Azo nitrogens decrease from 1 to 16 hours. There is a loss in intensity and broadening out of the azoxy peak at 323.3 ppm, with concomitant upfield shift of the peak maximum to 314.8 ppm. This suggests degradation or transformation of azoxy groups, and the possible formation of nitrogens such as indazoles, nitrones, or imines.
