# Supplementary material for: 13C and 15N NMR identification of product compound classes from aqueous and solid phase photodegradation of 2,4,6-trinitrotoluene
Source: PLoS One. 2019 Oct 22;14(10):e0224112. doi: 10.1371/journal.pone.0224112 (PMC6804990; doi:10.1371/journal.pone.0224112)
Supplement: S2 Text — (DOCX) [file pone.0224112.s003.docx]

**S2 Text**

**Solid State CP/MAS ^13^C NMR Spectra of Pond Water Solids and Photolysates of T^15^NT in Pond Water.**

The control solid state CP/MAS ^13^C NMR spectrum of the pond water solids shows bicarbonate, the major peak at 168 ppm, and broad peaks of lower intensity characteristic of aquatic NOM: 30 ppm, alkyl carbons; 73 ppm, O-alkyl, including carbohydrate carbons; 101 ppm, anomeric and protonated aromatic carbons; 129 ppm, aromatic carbons; 177 ppm, carboxyl carbons (S9A Fig). The contribution from this background DIC and DOC complicates interpretation of the CP/MAS ^13^C NMR spectra of the photolysates from sunlight and lamp irradiations of T^15^NT in the pond water (S9B-C Fig). Nevertheless, the spectrum of the sunlight photolysate shows peaks at 124, 135, and 149 ppm that are derived from the TNT, as well as the residual methyl carbons at 15 ppm, the latter of which was not visible in the corresponding liquid state spectrum of Fig 9C. The peak at 168 ppm corresponding to the bicarbonate from the pond water obscures any carboxyl or amide carbons that may be present from the photochemical transformation of the TNT. The CP/MAS spectrum of the photolysate from the 1 hour lamp irradiation shows the same peaks as in the sunlight photolysate, but with different ratios of peak intensities (S9C Fig). The TNT derived peaks at 149, 124, and 15 ppm are notably present.
