# Supplementary figures and images for: 13C and 15N NMR identification of product compound classes from aqueous and solid phase photodegradation of 2,4,6-trinitrotoluene
Source: PLoS One. 2019 Oct 22;14(10):e0224112. doi: 10.1371/journal.pone.0224112 (PMC6804990; doi:10.1371/journal.pone.0224112)

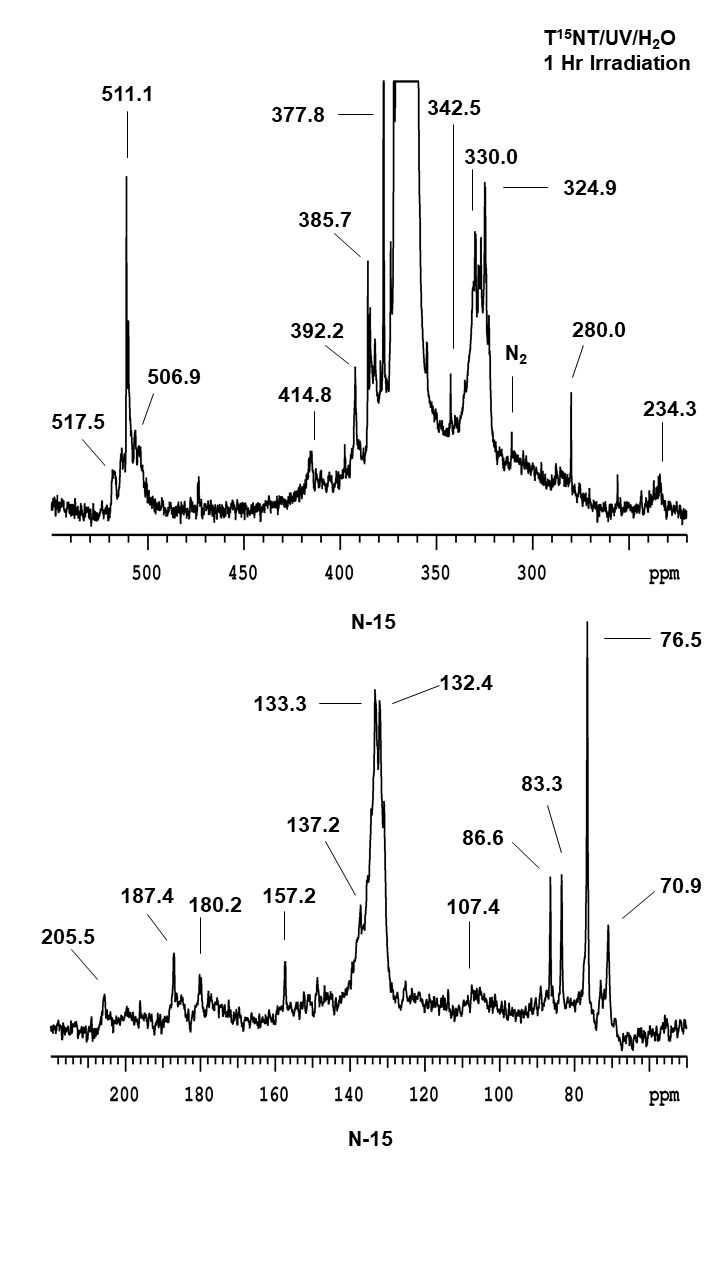

Supplement: S5 Fig — Horizontal and vertical scale expansions. (TIF) [file pone.0224112.s008.tif]
